# The protein architecture in *Bacteria* and *Archaea* identifies a set of promiscuous and ancient domains

**DOI:** 10.1371/journal.pone.0226604

**Published:** 2019-12-19

**Authors:** Rafael Hernandez-Guerrero, Edgardo Galán-Vásquez, Ernesto Pérez-Rueda

**Affiliations:** 1 Instituto de Investigaciones en Matemáticas Aplicadas y en Sistemas, Universidad Nacional Autónoma de México, Unidad Académica Yucatán, Mérida, Yucatán, México; 2 Departamento de Ingeniería de Sistemas Computacionales y Automatización, Instituto de Investigaciones en Matemáticas Aplicadas y en Sistemas, Ciudad Universitaria, Universidad Nacional Autónoma de México, Ciudad de México, México; 3 Centro de Genómica y Bioinformática, Facultad de Ciencias, Universidad Mayor, Santiago, Chile; Griffith University, AUSTRALIA

## Abstract

In this work, we describe a systematic comparative genomic analysis of promiscuous domains in genomes of *Bacteria* and *Archaea*. A quantitative measure of domain promiscuity, the weighted domain architecture score (WDAS), was used and applied to 1317 domains in 1320 genomes of *Bacteria* and *Archaea*. A functional analysis associated with the WDAS per genome showed that 18 of 50 functional categories were identified as significantly enriched in the promiscuous domains; in particular, small-molecule binding domains, transferases domains, DNA binding domains (transcription factors), and signal transduction domains were identified as promiscuous. In contrast, non-promiscuous domains were identified as associated with 6 of 50 functional categories, and the category Function unknown was enriched. In addition, the WDASs of 52 domains correlated with genome size, i.e., WDAS values decreased as the genome size increased, suggesting that the number of combinations at larger domains increases, including domains in the superfamilies Winged helix-turn-helix and P-loop-containing nucleoside triphosphate hydrolases. Finally, based on classification of the domains according to their ancestry, we determined that the set of 52 promiscuous domains are also ancient and abundant among all the genomes, in contrast to the non-promiscuous domains. In summary, we consider that the association between these two classes of protein domains (promiscuous and non-promiscuous) provides bacterial and archaeal cells with the ability to respond to diverse environmental challenges.

## Introduction

Since Wetlaufer [1] described that consecutive residues in polypeptide chains tend to fold into more or less compact modules called domains, it has been generally accepted that domains are the protein evolutionary modules, and modular reuse has been demonstrated in all domains of life [2]. This modularity might be advantageous to the organisms, allowing signaling proteins to expand their regulatory linkages and may elicit a broader range of control mechanisms either via modular combinations or through modulation of inter-modular linkages [3].

Therefore, proteins are modular, and it has been suggested that large proteins contain multiple domains, either with the same or with different structural folds [4]. In this regard, comparative analyses of proteins have revealed a substantial fraction of multidomain proteins in eukaryotic organisms [5, 6]. Similar conclusions have been reached through the analysis of domain combinations, namely, that more complex organisms display greater proportions of domain combinations and duplications [7, 8].

Recently, some of these domain combinations have been identified as stable during evolution, whereas others have been highly mobile [9]. Accordingly, domains are defined as promiscuous if they are combined with many other domains, suggesting that such combination of domains with different structures and functions is a major source of diversity and modulation of molecular functionality. A second group of proteins whose domain architecture is conserved has been identified, and this group contains domains with few associations, or monolithic domains. A recent analysis showed that the increase in domain combinations of 19 families of DNA-binding transcription factors (TFs) correlated with the complexity of the organisms studied; however, it is uncertain if this correlation can be extended to additional domains [10] or is a particular observation for proteins devoted to gene regulation.

Therefore, in this work we evaluated the tendency of protein domains to combine with other domains, i.e., domain promiscuity, in proteins of bacterial and archaeal genomes. We assigned domains based on a Superfamily hidden Markov models (HMM), and a quantitative measure of domain promiscuity, the weighted domain architecture score (WDAS), was used to analyze 1319 non-redundant bacterial and archaeal genomes. We further attempted to correlate this measure with function, genome size and protein fold ancestrality.

## Materials and methods

### Prokaryotic genomes

A total of 5321 genomes corresponding to *Bacteria* and *Archaea* domains were downloaded from the NCBI server and filtered to exclude redundancy, using the criteria described in [11]. We performed comparative analyses using the open reading frames (ORFs) that encode predicted proteins in all organisms. Hence, a total of 1214 bacteria; and 105 archaeal non-redundant genomes were considered for this analysis.

### Superfamily domain assignments

The predicted protein sequences (the proteome) in all organisms were scrutinized to assess their domain organization by using the Superfamily database assignments [12]. A library of 1659 Superfamily HMMs was compared to the protein sequences by using the script hmmscan.pl (HMMer 3.1b2 [13]) provided by Superfamily (v. 2015) that considers the following parameters: -E 10 -Z 15438 -hmmscan -threads 4. To generate consistent assignments from the output files, we ran the script ass3.pl -t n -f 4 -e 0.0001 (this e-value is considered as conservative default).

### Evaluation of domain architecture diversity

The WDAS, a measure of domain architecture diversity, was computed as described elsewhere [14]. The WDAS considers the proteins containing a domain and the total number of proteins per genome via the inverse abundance frequency (IAF) statistic, as follows:

$$IAF(d)={log}_{2}\frac{Pt}{Pd}$$

where *P*_*t*_ is the total proteins per genome and *P*_*d*_ is the number of proteins with domain *d*.

To determine the diversity of architectures associated with a specific domain, the inverse variability (IV) is obtained from the inverse of the number of distinct partner domain families at the N- and C-terminal sides adjacent to a domain. The definition of the IV of a domain, *d*, is:

$$IV(d)=\frac{1}{fd}$$

where *f*_*d*_ is the number of different domain families adjacent to domain *d*.

Finally, the WDAS of a domain is the product of the IAF and the IV:

WDAS=IAF×IV

The associations between the WDAS and genome size (measured in ORFs) were binned in 13 intervals without overlaps, with a width of 836 ORFs, as previously described [10]. In brief, the number of windows was calculated by using the Sturges’s formula: k = 1+ log2N where *k* is the number of equal classes and N the number of data, rounding to the nearest integer, the *k* value. Thus, the width of classes was determined with the equation: c = R/k where R = high value–low value (Genome size in ORFs). Values resulting from the application of the above formulas were k = 13 and c = 836, thus 13 windows without overlaps were used with a width of 836 ORFs [10].

### Promiscuity identification

In order to define the threshold to separate a domain as promiscuous and non-promiscuous, we computed an accumulative plot for the number of enriched functions associated to promiscuous and non-promiscuous domains at different scores of WDASs. In a posterior step, both datasets were adjusted with polynomial regression (S1 Fig). From this plot we found an increasing between the first, second and third intervals, that corresponds to values between 0 and 3.0 of WDAS. The numbers of enriched functions decays when a value of 0 to 4.0 was consider. Therefore, a WDAS of 3.0 could be considered as a threshold to identify promiscuous and non-promiscuous domains. This found is consistent, when the number of total domains per interval was consider, i.e. the number of domains exhibited a slight increase when values between 0 and 3.0 of WDAS were considered and that represents the 13.69% of the total of domains (S1 Fig). In summary, domains with a WDAS between 0 and 3.0 were defined as promiscuous.

### Functional enrichment

For each genome, two lists of protein domains were considered, using a threshold of ≤2.7 for promiscuous and ≥2.8 for non-promiscuous domains. An enrichment analysis using a hypergeometric test was conducted on these lists, considering 50 different functional categories obtained from the Superfamily (SUPFAM) and Structural Classification of Protein (SCOP) databases. Such a distribution describes the probability of finding *x* domains associated with a particular category in a list of interest *k*, from a set of *N* domains, with *m* domains associated with the same category. The P-values were corrected for multiple testing with Benjamini-Hochberger method to decreases the false discovery rate, and a statistical significance at P-value of <0.05 was set.

### Ancestrality of protein domains

To evaluate the protein domains ancestrality, superfamily assignments were associated to the phylogenomic tree of Protein Fold Families values previously described [15]. In brief, Caetano-Anolles et al. [15] considered a PSI-BLAST comparisons between PDB and genome sequences [16]; and the usage and sharing of protein folds was characterized with the: fold occurrence (*G*_*ij*_), average genome occurrence (*Ḡ*_*i*_), and fraction of genomes harboring a fold (*f*_*i*_). *G*_*ij*_ defines how often a protein fold (*i*) occurs in a given proteome (*j*). *Ḡ*_*i*_ represents averages of *G*_*ij*_ values. *Ḡ*_*i*_ and *f*_*i*_ measure the extent of fold sharing within each domain or combination of domains. Values were converted into linearly ordered multistate characters and normalized using an arbitrary scale (generally 0–20). Cumulative frequency plots were used to illustrate the accumulation of folds belonging to a protein class along a phylogenetic tree, and it is a function of distance in nodes from the root. These plots can be considered time plots of lineages [17] with a time axis defined in relative units.

## Results and discussion

### Distribution of domains according to superfamily assignments

It has been previously described that the increase in complexity of the domain organization of proteins would substantially contribute to the evolution of organismal complexity [2, 18]. In order to gain insights into protein architecture among prokaryotes, 1214 bacterial and 105 archaeal non-redundant genomes were analyzed in terms of their repertoire of protein domains. A coverage of assignment of 75.8% per genome, with some genomes with coverage of more than 90%, such as the gammaproteobacterium “*Candidatus Evansia muelleri*” (GCF_000953435.1), with coverage of more than 95% and representing the organism with the highest proportion of proteins with domain assignments. This result is consistent with the assignment percentage of proteins that were assigned with at least one *Superfamily* domain for Eukaryotes, Archaea, Bacteria and Viruses [12]. The genomes with the minor coverage of domain assignments corresponded to four mycoplasma genomes: *Mycoplasma haemofelis* Ohio2 (GCF_000186985.1), a hemoplasma species with a coverage of less than 30% and *M*. *suis* str. Illinois (GCF_000179035.2), *M*. *ovis* str. Michigan (GCF_000508245.1), and *Spiroplasma kunkelii* CR2-3x (GCF_001274875.1), with coverages of 33.4%, 33.6%, and 38.2%, respectively, suggesting that a large diversity of novel protein domains remains to be identified in those genomes and must be further explored (S1 Table).

We evaluated all bacterial and archaeal proteins in terms of their domain organization, identifying that 46.7% of proteins contain one domain, 31.3% contain two domains, 11.2% have three domains, and 10.6% contain more than four domains. It is evident from this distribution that more than 53% are multidomain proteins. The proportion of multidomain proteins follows a linear distribution, where large genomes contain a large proportion of two-, three-, or four-domains proteins, suggesting a positive correlation (R^2^ = 0.955) between the abundance of multidomain proteins and the number of proteins per genome (S2 Fig and S1 Table), when a non-linear least squared to fit the dataset was used.

**Fig 1:**
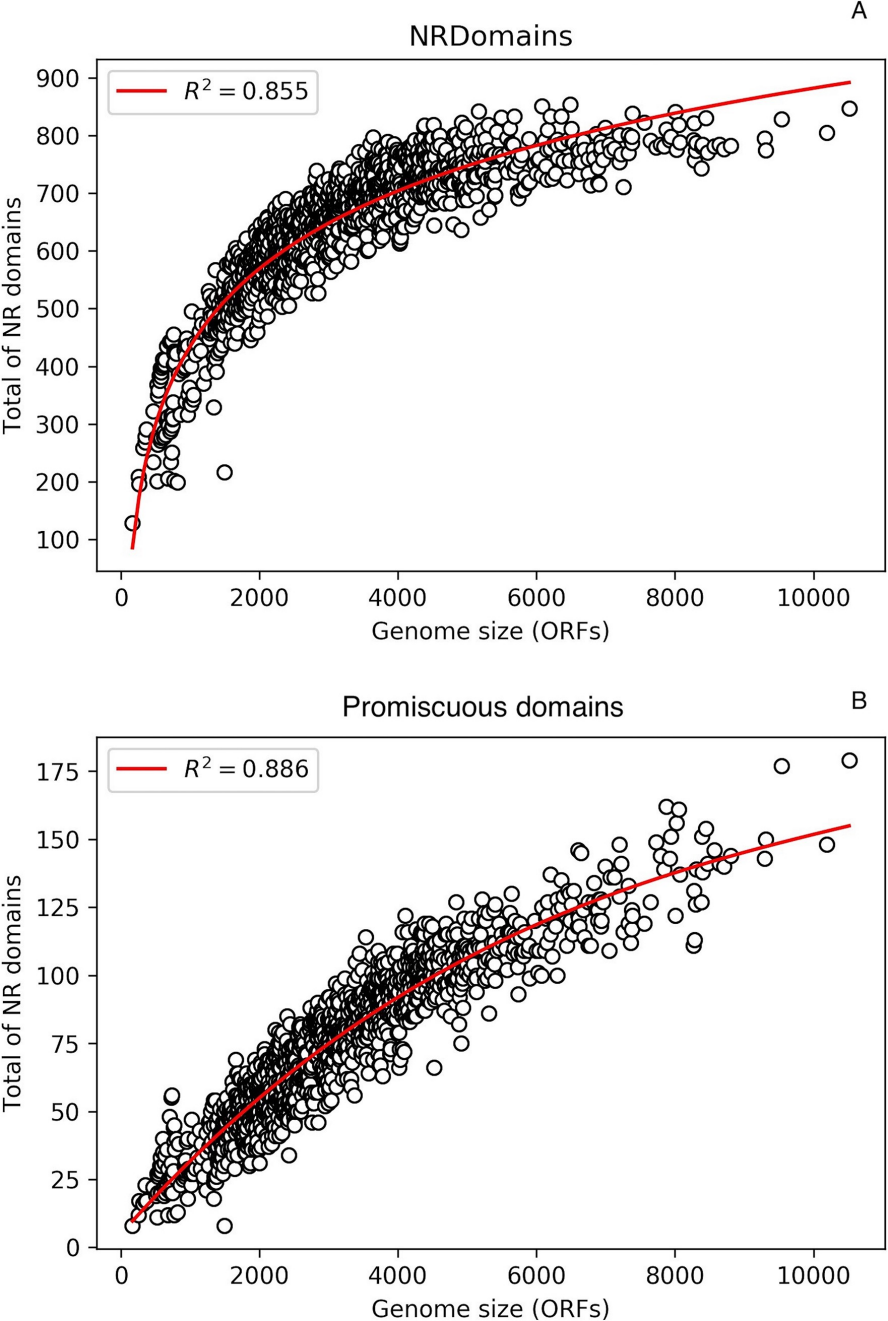
**The total number of non-redundant domains follows a logarithmic distribution (A), whereas promiscuous domains follow a polynomial distribution (B).** On the X-axis is the genome size (in ORFs), and on the Y-axis is the total number non-redundant (NR) domains identified. A non-linear least squared to fit the dataset was used.

In this regard, domains are present in various combinations in multidomain proteins. Thus, in this work, promiscuous domains were defined as domains that reside in many different combinations, i.e. combined with many other domains, a property that suggests a high degree of flexibility to combining with other different domains. Hence, to determine the diversity of structural domains associated with the bacterial and archaeal genomes, we evaluated the repertoire of all non-redundant domains per genome. As shown in Fig 1, organisms with reduced genomes (less than 2000 ORFs) contained a low proportion of non-redundant domains (less than 500 different protein domains) with an increase in organisms with more than 2000 ORFs (around 700 protein domains); however, the maximum number of different domains reached a plateau of around 800 different protein domains in organisms with more than 5000 ORFs. Therefore, small organisms exhibit a low diversity of different structural domains, suggesting that superfamilies at large genomes have increased their repertoire mainly via diverse duplication events followed by evolutionary divergence, as it has been previously suggested in eukarya [19]. In this manner, organisms could recycle protein domains to increase their repertoire and respond to diverse environmental challenges.

### Protein architecture provides functional diversity to bacterial and archaeal genomes

Based on the previous result and considering the hypothesis of domain recycling, we evaluated the protein architectures of the 1317 superfamilies. To this end, the complete set of proteins in 1320 bacterial and archaeal genomes was analyzed in terms of structural domain composition, according to the formula described in methods, with the WDAS, a measure of domain promiscuity, determined and plotted as a function of the genome size (see S1 File). Based on the WDAS, we defined that values closer to 0 represent promiscuity (diversity of their protein domain architectures), and higher values suggest no diversity in the protein architecture and, consequently these protein domains must be considered non-promiscuous, or monolithic.

To evaluate the set contribution of promiscuous and non-promiscuous domains to the total repertoire of domains associated with all the genomes, those domains with a WDAS between 0 and 3.0 (See methods) were considered promiscuous. Based on this definition, we found that an average less than 100 different domains per genome that corresponds to around 11.34% of the total protein domains per genome can be considered promiscuous (Fig 1), and species containing the highest proportions of this class of domains are also associated with large bacterial genomes, such as *Archangium gephyr* (GCF_001027285.1_ASM102728v1) and *Sorangium cellulosum* So0157-2 (GCF_000418325.1_ASM41832v1), among others. This finding is interesting because it shows that a low proportion of domains are associated with the highest number of architectural combinations, compared to non-promiscuous domains.

### Functional association of promiscuous domains

**Fig 2:**
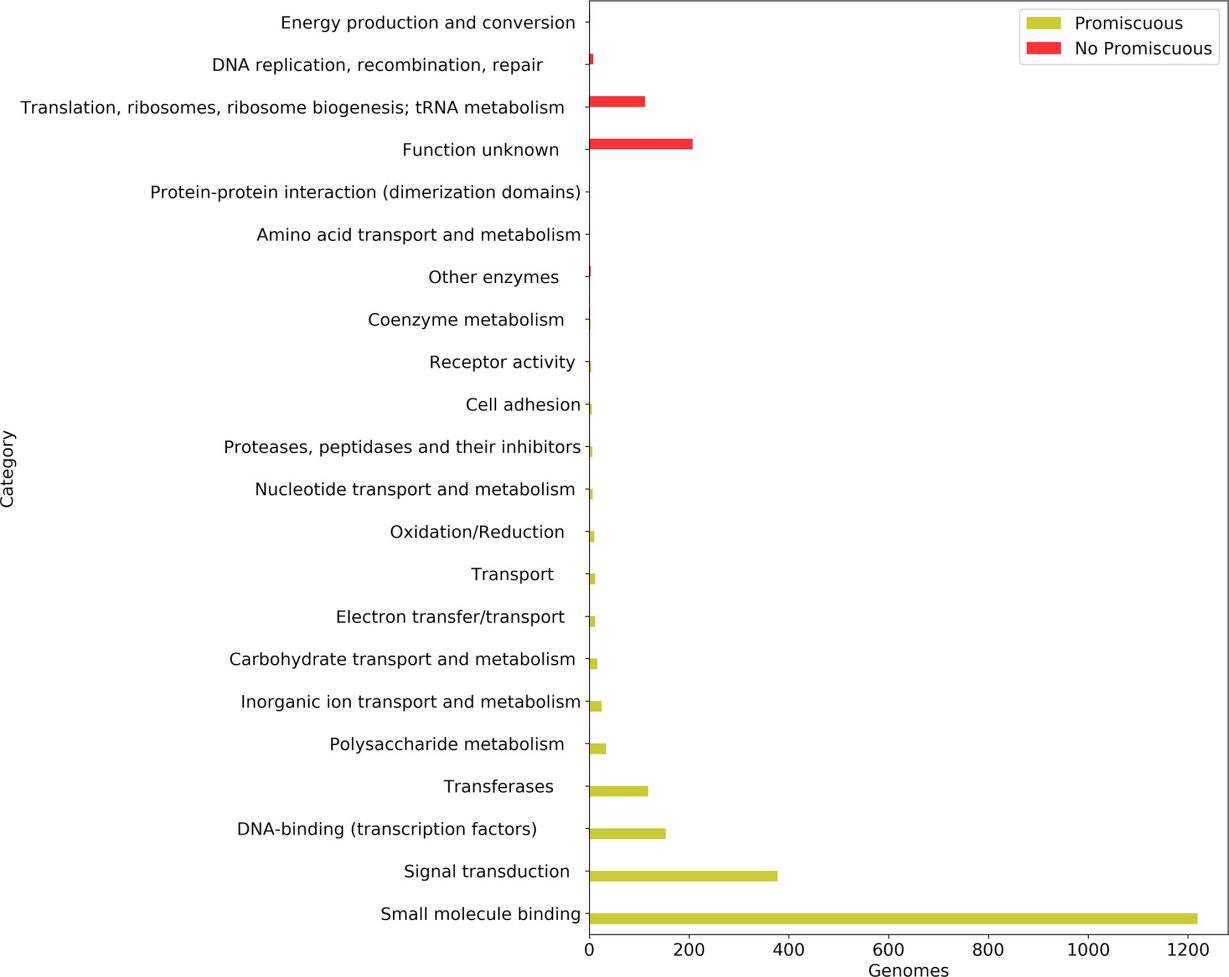
Functional enrichment analysis on promiscuous and non-promiscuous domains per genome. On the X-axis is the total number of genomes with an enriched function, and on the Y-axis are the functional categories.

Based on the definition of promiscuity, it was found that the proportion of promiscuous domains represents less than 10% of all non-redundant domains per genome, suggesting that a small fraction of the total repertoire of domains is determinant for the diversity of combinations of architectural identified so far. Therefore, in order to determine the preferential functions of promiscuous and non-promiscuos domains per genome, both datasets were evaluated, considering the functional annotations deposited in the SUPFAM and SCOP databases. In these databases, 50 different functional groups have been determined, and they are classified in eight broad categories (General; Information; Metabolism; Processes_EC, Processes_IC, and Regulation; Other; and N_A). From this analysis, 18 functional categories were identified as significantly enriched in the promiscuous domains in at least one genome, and in particular the Small-molecule binding domains were identified as enriched in 1219 genomes, whereas Signal transduction was enriched in 377 genomes and DNA-binding (transcription) and transferases were enriched in 153 and 118 genomes, respectively. Finally, the other 14 functional categories identified as enriched in minor proportions, such as Polysaccharide metabolism and Inorganic ion transport and metabolism and, were associated with 34 and 25 genomes, respectively (Fig 2).

In contrast, non-promiscuous domains were associated with 6 functional categories, with Unknown function, Translation, ribosomes, ribosome biogenesis; tRNA metabolism and DNA replication, recombination, repair, the most enriched. These functions, associated to the non-promiscuous domains were identified in 207, 112 and 8 genomes, respectively. Therefore, functional associations were different between promiscuous and non-promiscuous domains, suggesting that bias to DNA binding, Transferases, Small-molecule binding and Signal transduction functions are specifically associated with promiscuous domains. Fig 2.

### Protein architecture as a function of genome size

In our second approach, we considered the domains as a function of their correlation to genome size, where the correlation between a WDAS for a given domain and the genome size (number of ORFs) could be positive or negative and a set of uncorrelated domains could be identified. To this end, R-values were calculated and two large groups were defined: those negatively correlated and those with no correlation. Therefore, we were interested in the set of negatively correlated domains (R-values between -1 and -0.4), because they showed WDAS values decreasing as a function of genome size, i.e., protein domains are less promiscuous in small genomes and they are more promiscuous in large genomes. In addition, only protein domains identified in more than 60% of the total genomes or 770 different genomes were considered in this analysis, because we were interested in those domains with a wide genomic distribution for our analysis (See S1 File).

From the complete set of 1317 protein domains analyzed, 52 protein domains had negative correlation values (R-values between -1 to -0.4), i.e., the number of architectures associated with them increased as a function of genome size (Table 1). In this category were, the protein domains primarily associated with small-molecule binding, such as (SSF:56176) FAD-binding domain, (SSF:52518) Thiamine diphosphate-binding fold (THDP-binding), (SSF:51905) FAD/NAD(P)-binding domain, (SSF:51735) NAD(P)-binding Rossmann fold domains, and (SSF:52540) P-loop–containing nucleoside triphosphate hydrolases, represented 23% of the total domains defined as promiscuous. The second group of promiscuous domains was associated with DNA-binding proteins, such as (SSF:46894) C-terminal effector domain of the bipartite response regulators, (SSF:47413) Lambda repressor-like DNA-binding domains, (SSF:46689) Homeodomain-like, and (SSF:46785) Winged helix DNA-binding domain, representing 11% of the domains associated with this functional category; and transferases (metabolism category), including (SSF:52151) FabD/lysophospholipase-like, (SSF:52317) Class I glutamine amidotransferase-like, (SSF:55729) Acyl-CoA N-acyltransferases (Nat), (SSF:53383) Pyridoxal phosphate (PLP)-dependent transferases, and (SSF: 53335) S-adenosyl-L-methionine-dependent methyltransferases, representing 10%; and finally other enzymes (metabolism) (9%), including (SSF:56235) N-terminal nucleophile aminohydrolases (Ntn hydrolases), (SSF:51338) Composite domain of metallo-dependent hydrolases, (SSF:51556) Metallo-dependent hydrolases, (SSF:56801) Acetyl-CoA synthetase-like, (SSF:53901) Thiolase-like, (SSF:56784) HAD-like, and (SSF:53474) alpha/beta-Hydrolases. Table 1.

**Table 1:** Promiscuous domains in *Bacteria* and *Archaea*.

SUPFAM ID	Function	Description	R-value	P-value	Genome distribution	Number of domains
46689	LA	Homeodomain-like	-0.447598969	2.11E-70	1206	63188
46785	LA	Winged helix DNA-binding domain	-0.424153905	2.22E-52	1306	102676
46894	LA	C-terminal effector domain of the bipartite response regulators	-0.550775615	3.17E-94	1114	22398
47336	S	ACP-like	-0.502184217	3.21E-102	872	11982
47384	T	Homodimeric domain of signal transducing histidine kinase	-0.465054433	1.88E-65	1180	30953
47413	LA	lambda repressor-like DNA-binding domains	-0.43766273	8.42E-77	1231	27826
48179	C	6-phosphogluconate dehydrogenase C-terminal domain-like	-0.511977864	4.88E-95	1280	16142
48452	RD	TPR-like	-0.50277309	1.28E-70	1218	42972
48498	K	Tetracyclin repressor-like, C-terminal domain	-0.447532579	1.91E-73	931	14919
50129	O	GroES-like	-0.538824071	9.75E-97	1116	13619
51182	EA	RmlC-like cupins	-0.496006478	4.30E-100	1201	16674
51338	RC	Composite domain of metallo-dependent hydrolases	-0.440319429	1.94E-76	1167	10765
51395	RD	FMN-linked oxidoreductases	-0.477243565	6.47E-93	1242	9334
51556	RC	Metallo-dependent hydrolases	-0.521924096	2.10E-101	1285	16908
51735	HA	NAD(P)-binding Rossmann-fold domains	-0.460691707	2.84E-70	1319	122830
51905	HA	FAD/NAD(P)-binding domain	-0.557529039	3.06E-93	1314	46246
52096	OA	ClpP/crotonase	-0.484902387	4.77E-76	1260	20743
52151	RB	FabD/lysophospholipase-like	-0.526822557	8.97E-88	967	6126
52172	T	CheY-like	-0.516686547	2.99E-78	1208	56901
52317	RB	Class I glutamine amidotransferase-like	-0.543988302	1.52E-80	1309	19566
52343	RA	Ferredoxin reductase-like, C-terminal NADP-linked domain	-0.408337767	1.52E-67	925	4940
52402	F	Adenine nucleotide alpha hydrolases-like	-0.463833236	2.48E-71	1319	24519
52518	HA	Thiamin diphosphate-binding fold (THDP-binding)	-0.405125258	5.96E-53	1311	25285
52540	HA	P-loop containing nucleoside triphosphate hydrolases	-0.524497666	4.13E-94	1319	293049
52833	RA	Thioredoxin-like	-0.466397534	2.88E-77	1307	35294
53098	F	Ribonuclease H-like	-0.436713312	2.92E-63	1317	22384
53187	OA	Zn-dependent exopeptidases	-0.458998335	1.23E-72	1295	16486
53335	RB	S-adenosyl-L-methionine-dependent methyltransferases	-0.551620835	2.47E-100	1317	60989
53383	RB	PLP-dependent transferases	-0.578930336	4.92E-122	1313	38176
53474	RC	alpha/beta-Hydrolases	-0.547861371	6.58E-114	1260	44812
53850	P	Periplasmic binding protein-like II	-0.476126611	1.73E-75	1301	65801
53901	RC	Thiolase-like	-0.550295571	1.30E-101	1268	21450
54292	RA	2Fe-2S ferredoxin-like	-0.456931722	8.47E-56	1096	8687
54427	RF	NTF2-like	-0.482112445	9.35E-87	880	9935
54593	RA	Glyoxalase/Bleomycin resistance protein/Dihydroxybiphenyl dioxygenase	-0.450965341	7.55E-79	1016	14244
54631	RF	CBS-domain	-0.411347444	1.65E-63	1283	12925
55729	RB	Acyl-CoA N-acyltransferases (Nat)	-0.456353703	1.03E-86	1250	34844
55781	T	GAF domain-like	-0.490340591	8.87E-102	1083	19207
55811	L	Nudix	-0.404505327	7.98E-71	1208	11668
55874	O	ATPase domain of HSP90 chaperone/DNA topoisomerase II/histidine kinase	-0.496149089	3.81E-69	1273	50342
55961	R	Bet v1-like	-0.419276746	1.15E-66	810	8377
56059	H	Glutathione synthetase ATP-binding domain-like	-0.46214717	4.47E-50	1275	18325
56112	OB	Protein kinase-like (PK-like)	-0.438363119	1.36E-76	1206	17033
56176	HA	FAD-binding domain	-0.472306546	6.13E-70	1213	9295
56235	RC	N-terminal nucleophile aminohydrolases (Ntn hydrolases)	-0.459305139	1.50E-57	1243	10121
56784	RC	HAD-like	-0.416096509	9.25E-60	1302	23797
56801	RC	Acetyl-CoA synthetase-like	-0.547745004	1.80E-109	1107	18746
63380	H	Riboflavin synthase domain-like	-0.410258238	7.10E-70	1144	7771
82866	RF	Multidrug efflux transporter AcrB transmembrane domain	-0.432469755	9.57E-65	1211	17967
88659	TA	Sigma3 and sigma4 domains of RNA polymerase sigma factors	-0.523716391	2.02E-78	1248	20632
88946	S	Sigma2 domain of RNA polymerase sigma factors	-0.585116314	1.62E-105	1155	16024
103473	P	MFS general substrate transporter	-0.483568169	5.55E-84	1285	39310

Columns are as follows: SUPFAM ID, Function, Description, R-value (correlation of WDAS score vs. genome size); P-value; number of genomes where the domain was identified; number of genomes for which the SUPFAM domain was identified and total number of domains in the indicated superfamily.

Functional categories. General: HA (Small molecule binding), R (General or several functions), RD (Dimerization domains). Information: K (Transcription), L (DNA replication, recombination, repair). Metabolism: C (Energy production and conversion), EA (Nitrogen metabolism), F (Nucleotide transport and metabolism), RA (Oxidation/Reduction), RB (Transferases), RC (Other enzymes). Processes_IC: O (Posttranslational modification, protein turnover, chaperones); OA (Proteases, peptidases and their inhibitors), P (Inorganic ion transport and metabolism), RF (Transport). Regulation: LA (DNA-binding transcription factors), OB (Kinases and phosphatases and inhibitors), T (Signal transduction), TA (Other regulatory function). Other: S (Function unknown).

In summary, the four functional categories more abundant associated to this set of promiscuous domains are related to metabolism (other enzymes, and transferases), DNA-binding and small-molecule binding. These functional categories suggest that these structural domains could be associated with ancient and global functions that define the basic functions of cellular maintenance, signaling and regulation of gene expression, and they are influenced by the dynamics of the genome architecture in bacteria [20]; i.e. an increase in genome complexity also increases the probable combinations of structural domains. In contrast, three main functions not associated with promiscuous domains were identified: Translation (SSF:100704), metabolism (redox reactions) (SSF:119536), and other enzymes (SSF:170266). These functions were identified in a small proportion of domains compared to promiscuous domains, suggesting that they are not as highly abundant as promiscuous domains.

Although there are diverse and interesting protein superfamily domains, there are two cases to illustrate the association of promiscuity with genome size: the P-loop–containing nucleoside triphosphate hydrolases domain (P-loop; SSF:52540) and the Winged helix-turn-helix domain (SSF:46785).

**Fig 3:**
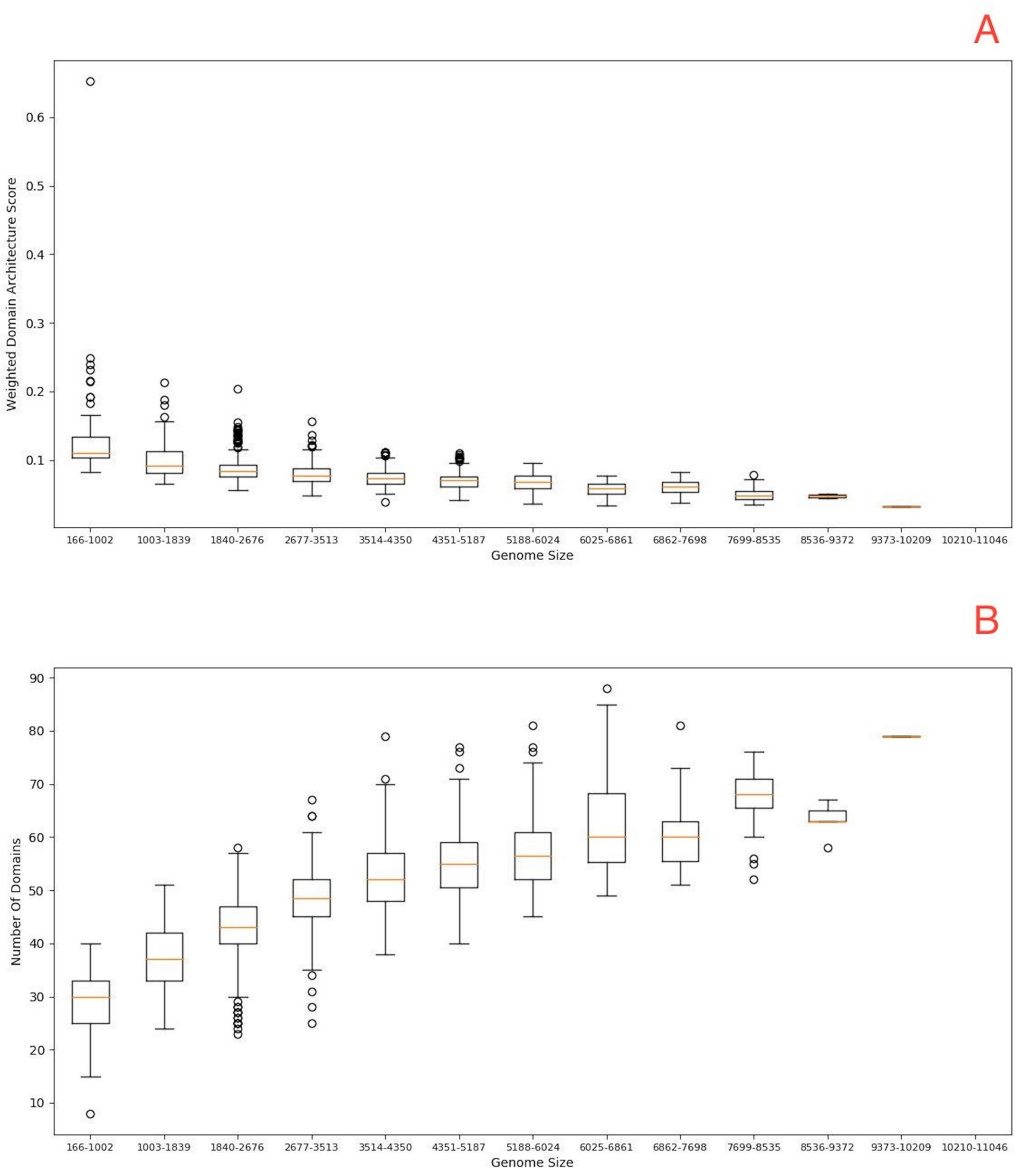
The architecture of the P-loop superfamily as a function of genome size. A) WDAS of the P- loop (R-value = -0. 52; p-value = 4.13E-94); B) number of different domains associated with the P-loop (R-value = 0.77, p-value = 4.84e-264). On the X-axis of each graph, genome size ranges are displayed in 13 windows, with a range of 836 ORFs each. On the Y-axis are the WDASs. The lines shown in the boxes are the median values. The whisker caps represent the minimum and maximum values.

The P-loop domain is the most prevalent domain of the nucleotide-binding protein folds, has been classified in the small-molecule binding functional category (HA) and represents 13.8% of the total domains identified as promiscuous. Proteins with this fold catalyze the hydrolysis of the beta-gamma phosphate bond of nucleoside triphosphate (NTP), and the energy from NTP hydrolysis is used to induce conformational changes in other molecules [21]. P-loop NTPases are characterized by two conserved sequence signatures, the Walker A motif (the P-loop proper) and Walker B motifs which bind the beta and gamma phosphate moieties of the bound NTP and a Mg^2+^ cation, respectively [22]. Thus, when we analyzed the number of different domains associated with the P-loop, we found a large number of related domains in larger genomes versus smaller genomes, suggesting that these superfamilies not only increase their association with other domains but also with different architectures (Fig 3).

**Fig 4:**
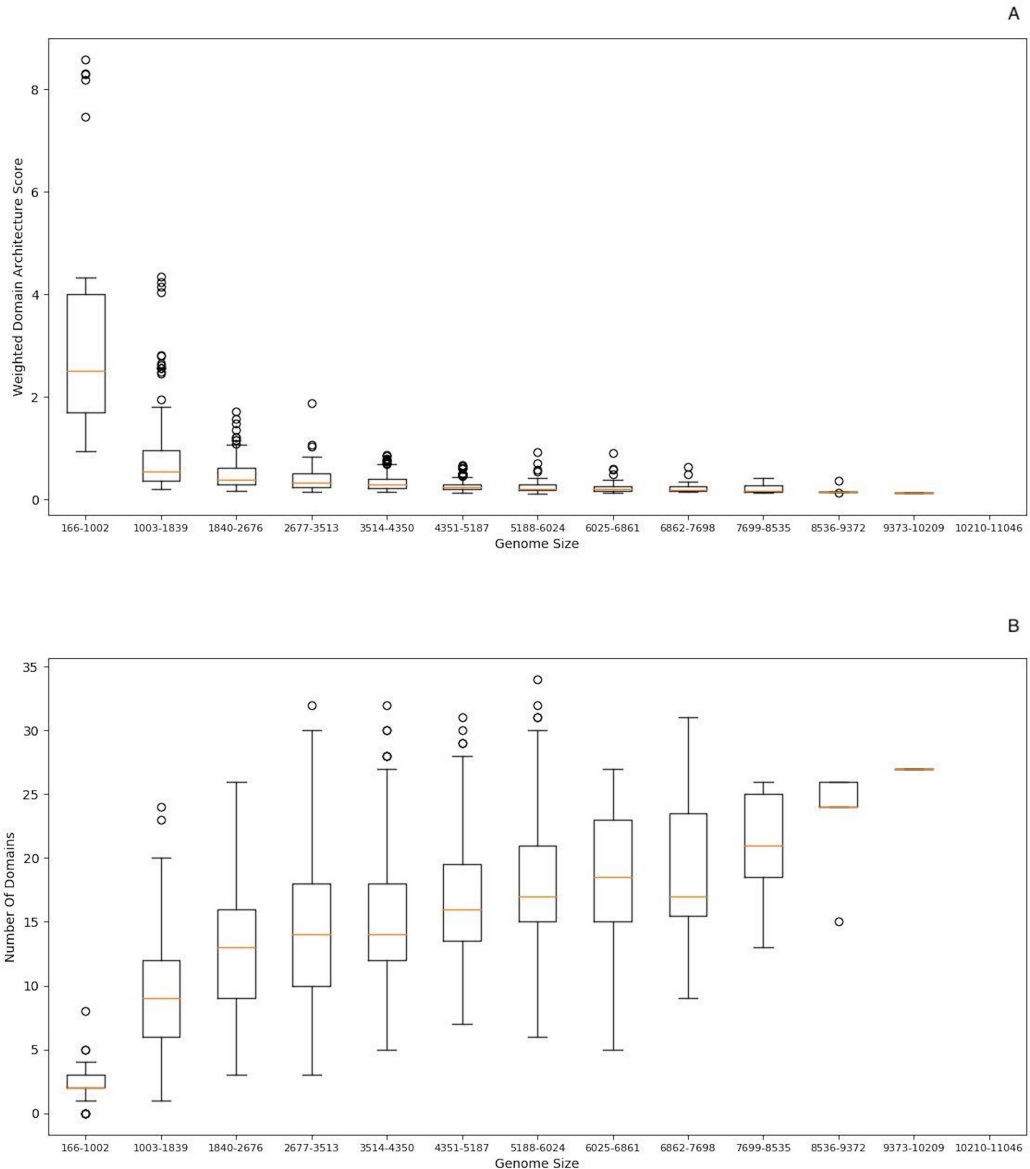
Architecture of the wHTH superfamily as a function of genome size. A) WDAS of the wHTH superfamily (R-value = -0.42; p-value = 2.22E-52); B) number of different domains associated with the wHTH (R-value = 0.545 and p-value = 3.07e-102). On the X-axes of each graph are genome size ranges, displayed in 13 windows, each with a length of 836 ORFs. On the Y-axes are the WDASs. The horizontal lines in the boxes are the median values. The whisker caps represent the minimum and maximum values.

A second representative example is associated with the Winged helix-turn-helix DNA-binding domain. These structures are characterized by the presence of a third alpha-helix and an adjacent beta-sheet and are central components for DNA binding; the “wings” are small beta-sheets [23]. The recognition helix binds as described for regular helix-turn-helix motifs (i.e., with contacts in the major groove of DNA), and the extra secondary structural elements make different DNA contacts, often with the minor groove or the backbone of DNA [24, 25]. The wHTH has been identified in almost all microorganisms, from *Bacteria* to *Archaea*, and includes diverse families, such as the Catabolite gene activator (CAP) family, in which global regulators (CRP and FNR) have been described in the bacterium *Escherichia coli* [26], the heat shock and E2F/DP TFs, and the Ets domain family, among others [25]. The WDAS associated with the wHTH decreases as a function of the genome size, where larger genomes contain a higher proportion of these proteins, with diverse architectures and a high diversity of structural domains (Fig 4).

In summary, we suggest that the set of 52 superfamilies follows trends similar to those of the P-loop and wHTH superfamilies, reinforcing the notion that these superfamilies not only increase their associations with other domains but also their architecture diversity.

### Promiscuous domains are ancient

To evaluate how promiscuity developed during evolution, we mapped the WDASs associated with all domains against the ancient approach described by [6]. This analysis is based on the hypothesis that the most promiscuous domains could be also ancient domains. Therefore, the 52 protein domains identified as promiscuous associated to the genome size and non-promiscuous were traced along with their ancestry according to the approach described by Caetano-Anolles et al. [15]. S3 Table. In brief, Caetano-Anolles et al. consider the timeline of protein domain evolution spanning ~3.8 billion years of evolution, where a score of 0 represents the origin of domains and a score of 1 represents the present day. In this way, ancestrality is defined by ancestries of protein domain constituents derived from a structural phylogenomic census [6, 15].

**Fig 5:**
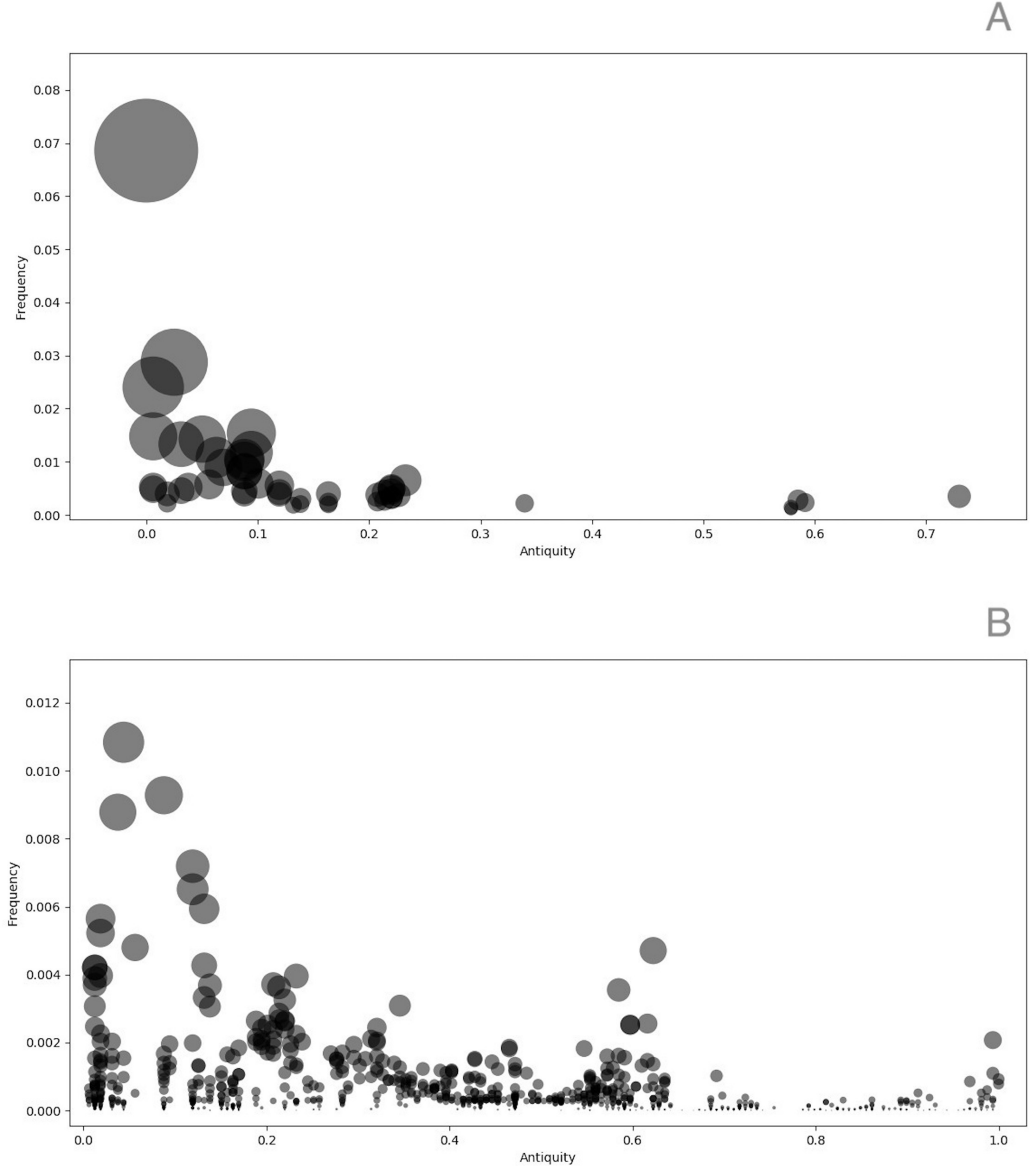
Antiquity and abundance of structural domains. A) Promiscuous and B) non-promiscuous domains. On the X-axes are antiquity assignments, i.e., how ancient each structural domain present in the universal enzymatic reactions is, as suggested by Caetano-Anolles et al [15]; a score of 0 represents an ancient event, whereas 1.0 represents recent domain emergence. On the Y-axis is the abundance (frequencies) of the superfamilies. The size of a circle represents the proportion of each domain in relation to the total protein domains.

In Fig 5, we show the frequencies of the 52 promiscuous domains that correlated with the genome size and identified in this analysis as a function of the ancestry score. From this analysis, two main results can be described: the first is that promiscuous domains are mainly associated with ancient evolutionary events (i.e., antiquity scores close to 0), with few recent emergence events (scores closer to 1) (Fig 5A). In contrast, no promiscuous domains are distributed along the whole antiquity timeline, suggesting that their emergence has been at diverse times in evolution (Fig 5B). In addition, we determined that promiscuous domains are more abundant than non-promiscuous domains. In addition, a Mann-Whitney U test was used to compare the two datasets, finding an U-value of 1064 and because the distribution is approximately normal the *Z*-Score is -10.62215 with a *p*-value of < 0.00001, being significant at *p* < .01. In other words, the difference between the promiscuous and non-promiscuous datasets is statistically significant. In summary, the promiscuous domains are abundant and ancient, such as the P-loop–containing nucleoside triphosphate hydrolases (SF:52540) and the DNA-binding domain (wHTH), because they are recurrent and most ancestral among the universal domains, in comparison to non-promiscuous domains. Therefore, the interplay of promiscuous and no-promiscuous domains determines the architecture of proteins in all the genomes.

## Conclusions

In this work, we evaluated 1317 superfamilies for their architectures, their abundance in bacterial and archaeal genomes, their functional roles and their antiquities. Our data reinforce that increased gene complexity also requires the development of mechanisms for gene regulation at the transcription level and small-molecule binding, where the P-loop and the wHTH are the most significant examples, suggesting that the interplay of these structural domains could increase the ability of an organism to recognize and respond to diverse environmental stimuli. These findings are relevant in the context of functional categories, because promiscuous domains are mainly associated with basic metabolic, signal transduction, and gene regulation functions. We also found 52 domains whose promiscuity value or WDAS depends on genome size, and this was reinforced by the number of domains associated with each of them. In summary, we consider the association between these two classes of protein domains (promiscuous and non-promiscuous) has provided, since ancestral times, bacterial and archaeal cells with the ability to respond to diverse environmental challenges.

## Supporting information

S1 Fig Distribution of enriched functions for Promiscuous and non-promiscuous domains at different thresholds. The distributions were adjusted using the function polyfit of the numpy package in python, and different degree of polynomial regression were tested to maximize R^2^. A polynomial regression of grade 4 was found as the best for both datasets.
(TIFF)

S2 Fig The total of multidomain proteins per organisms follows a lineal distribution. On the X-axis is the number of ORFs per genome, and the Y-axis shows the total of proteins with two or more domains. Each open circle denotes a genome. (R-value = 0.955, p-value < 0.0). A non-linear least squared to fit the dataset was used.
(TIFF)

S1 Table Domain assignments by SUPFAM of 1507 organisms based on the NCBI classification system. Columns correspond to: Genome ID; Total of Proteins per genome (ORFs); NRProteins_Assigned (total of proteins with one non redundant domain); Coefficient (coverage = total of NR proteins / ORFs); NRDomains; 1Domain_count; 2Domain_Count; 3Domain_Count; > = 4DomainCounter.
(TXT)

S2 Table Domain distribution per taxonomical classification. Columns correspond to: Domain; C.C.; p-value, Gen_Count; Domain_Count Function; Group_Count; Firmicutes; Proteobacteria; Euryarchaeota; Actinobacteria; Tenericutes; Chlamydiae: Chlorobi; Crenarchaeota; Fusobacteria; Bacteroidetes; Spirochaetes; Cyanobacteria; Deinococcus; Thermus; Thermotogae: Aquificae; Chloroflexi; Gemmatimonadetes: Deferribacteres; Acidobacteria; Candidatus Korarchaeota; Verrucomicrobia: Elusimicrobia; Dictyoglomi; Nitrospirae; Fibrobacteres; Synergistetes; Thermobaculum; Planctomycetes; Cloacimonetes; Chrysiogenetes: Thermodesulfobacteria; Ignavibacteriae; Caldiserica; Thaumarchaeota: Armatimonadetes; Taxonomy total distribution.
(TXT)

S3 Table Antiquity and abundance of structural domains. Columns correspond to: Domain; Function; SCOP; Description; ancient_order; Ancient_score; R-value; Total_Domains; Archaea_Domains; and Bacteria_Domains.
(TXT)

S1 File Boxplot of the architecture of the 52-superfamily domains as a function of genome size. On the X-axis of each graph, genome size ranges are displayed in 13 windows, with a range of 836 ORFs each. On the Y-axis are the WDASs. The lines shown in the boxes are the median values. The whisker caps represent the minimum and maximum values. Superfamily IDs correspond to the names in Table 1.
(ZIP)
