# Supplementary figures and images for: The protein architecture in Bacteria and Archaea identifies a set of promiscuous and ancient domains
Source: PLoS One. 2019 Dec 19;14(12):e0226604. doi: 10.1371/journal.pone.0226604 (PMC6922389; doi:10.1371/journal.pone.0226604)

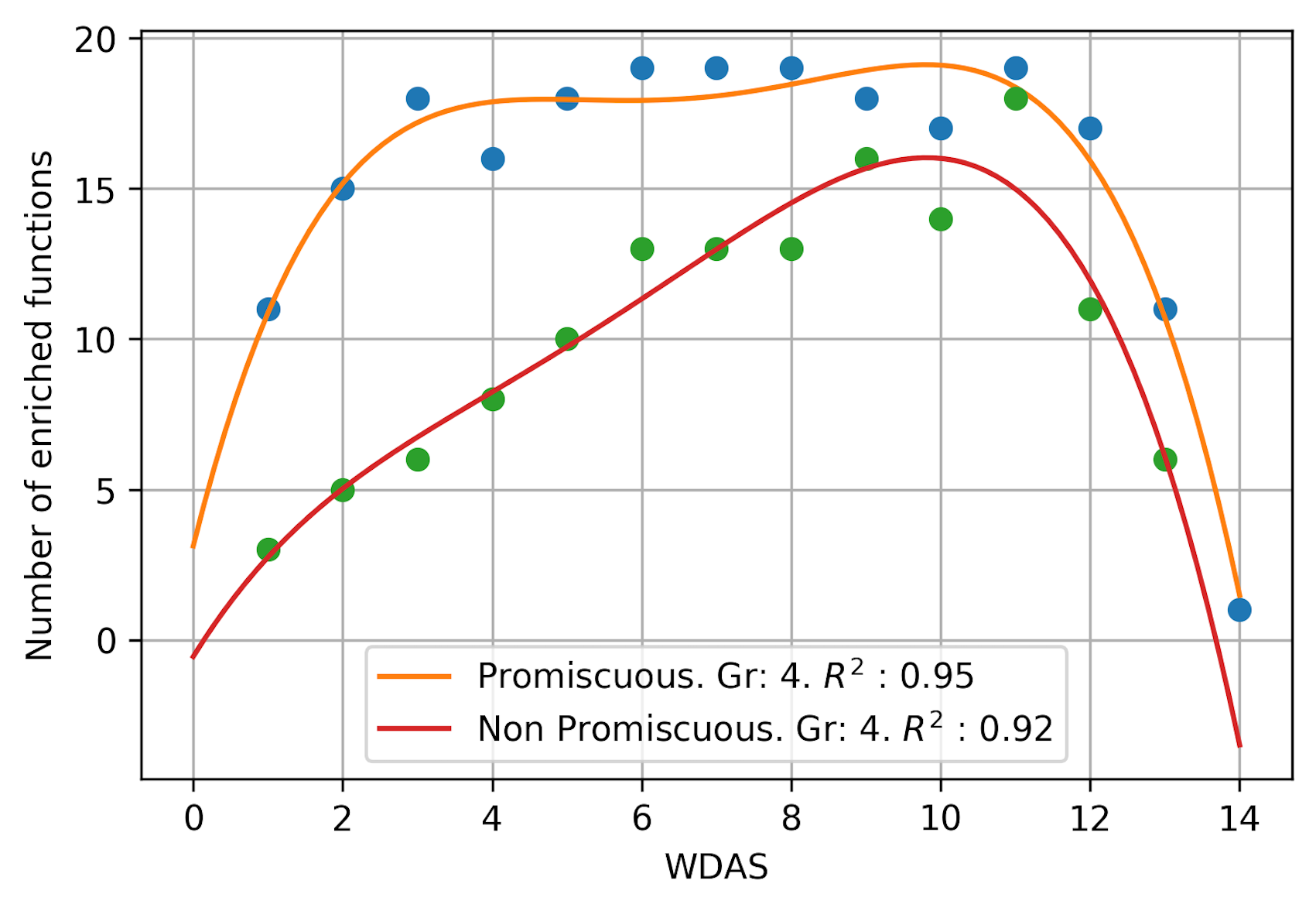

Supplement: S1 Fig — The distributions were adjusted using the function polyfit of the numpy package in python, and different degree of polynomial regression were tested to maximize R2. A polynomial regression of grade 4 was found as the best for both datasets. (TIFF) [file pone.0226604.s001.tiff]

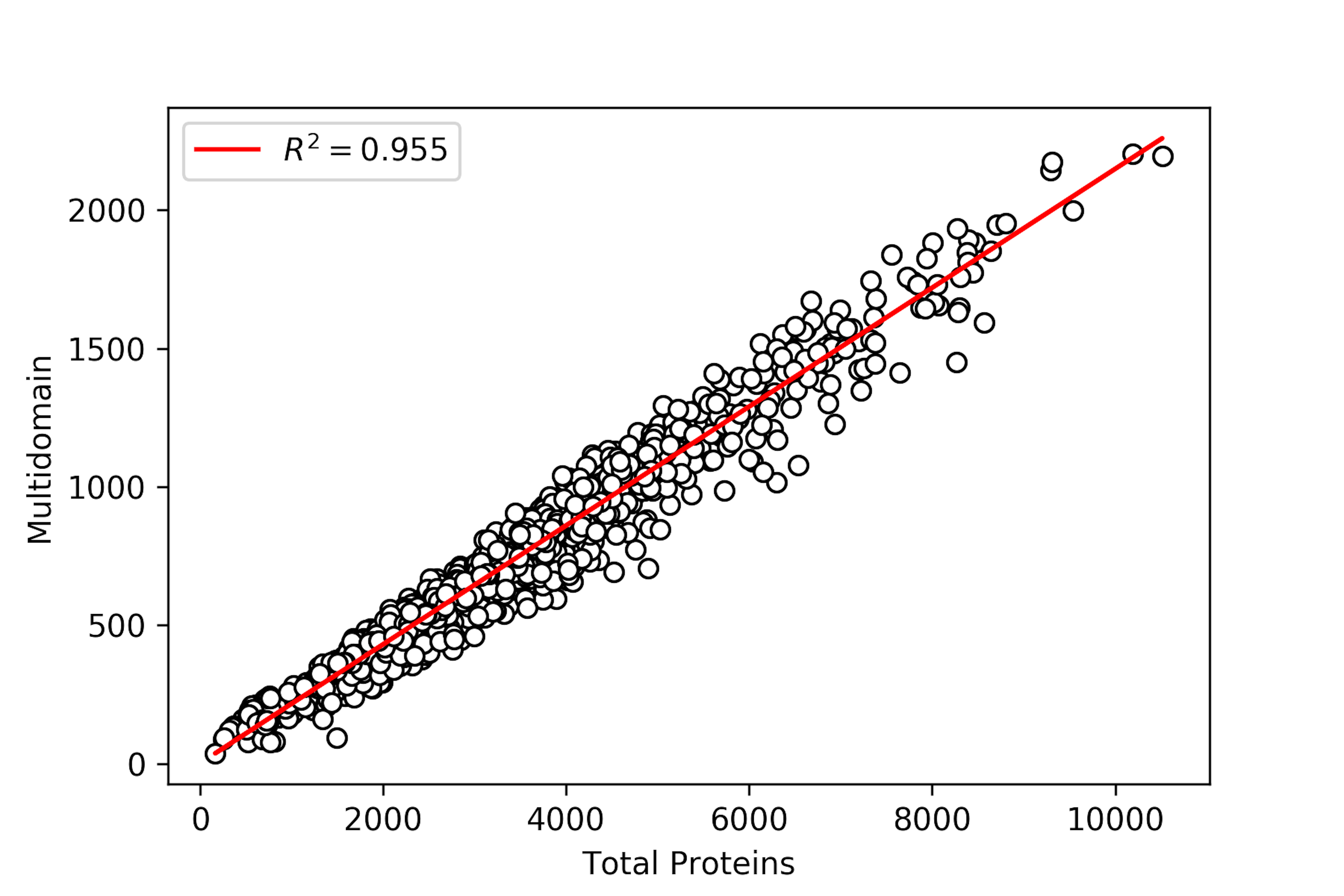

Supplement: S2 Fig — On the X-axis is the number of ORFs per genome, and the Y-axis shows the total of proteins with two or more domains. Each open circle denotes a genome. (R-value = 0.955, p-value < 0.0). A non-linear least squared to fit the dataset was used. (TIFF) [file pone.0226604.s002.tiff]

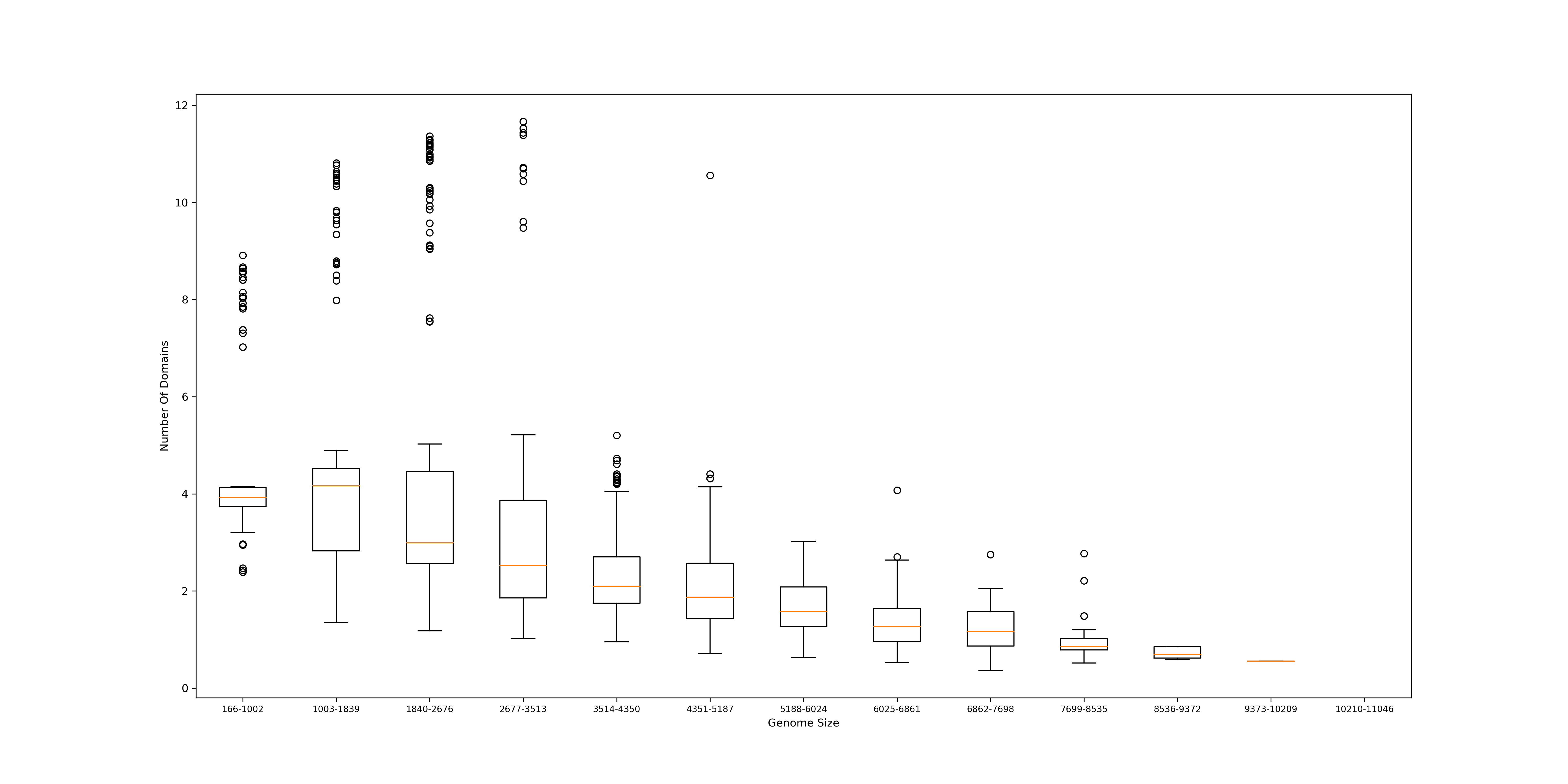

Supplement: S1 File — On the X-axis of each graph, genome size ranges are displayed in 13 windows, with a range of 836 ORFs each. On the Y-axis are the WDASs. The lines shown in the boxes are the median values. The whisker caps represent the minimum and maximum values. Superfamily IDs correspond to the names in Table 1. (ZIP) [file pone.0226604.s006.zip › Supplemnetary_material_S1/Figure_WSByIntervals_88659.png]

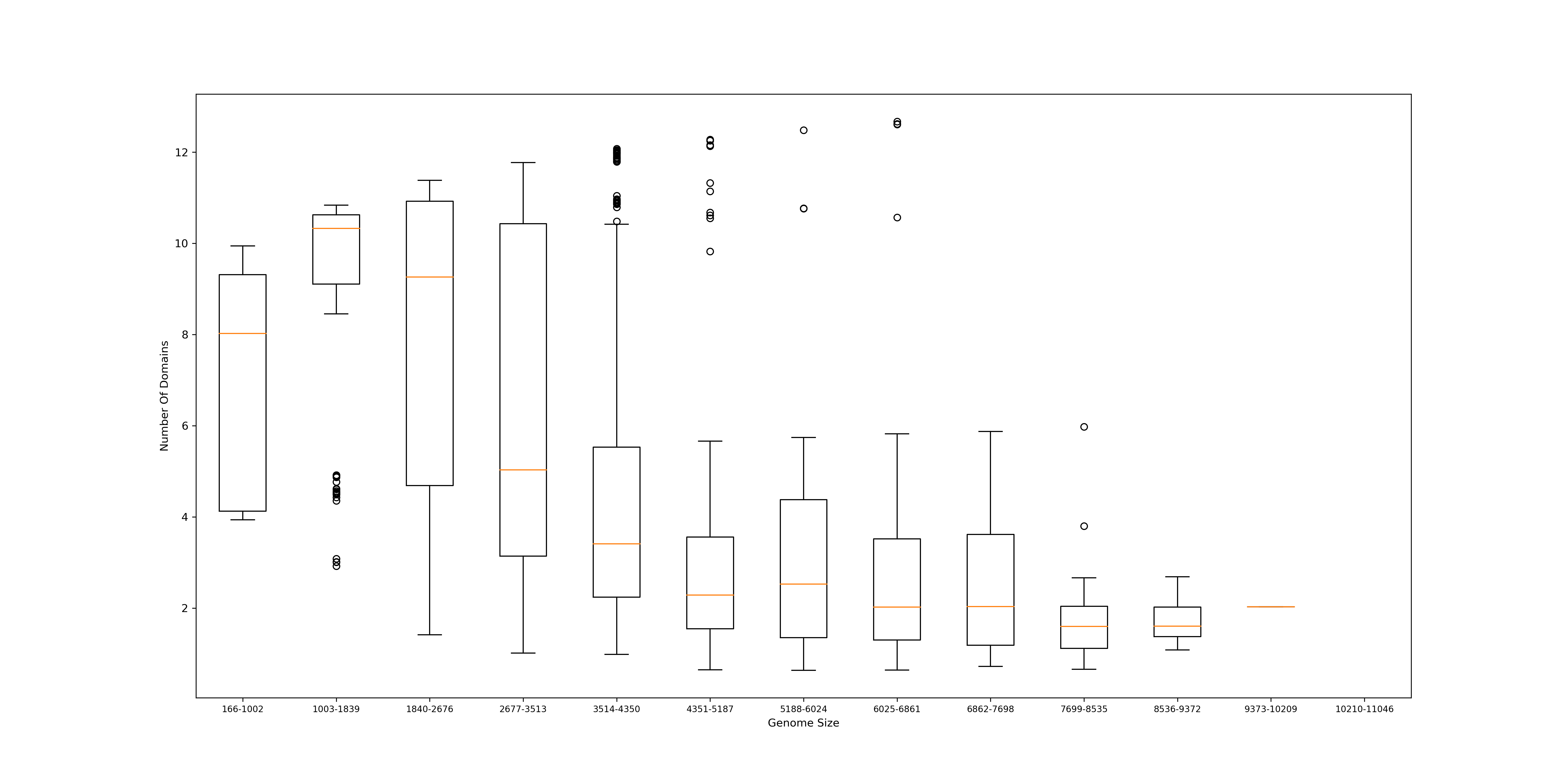

Supplement: S1 File — On the X-axis of each graph, genome size ranges are displayed in 13 windows, with a range of 836 ORFs each. On the Y-axis are the WDASs. The lines shown in the boxes are the median values. The whisker caps represent the minimum and maximum values. Superfamily IDs correspond to the names in Table 1. (ZIP) [file pone.0226604.s006.zip › Supplemnetary_material_S1/Figure_WSByIntervals_52343.png]

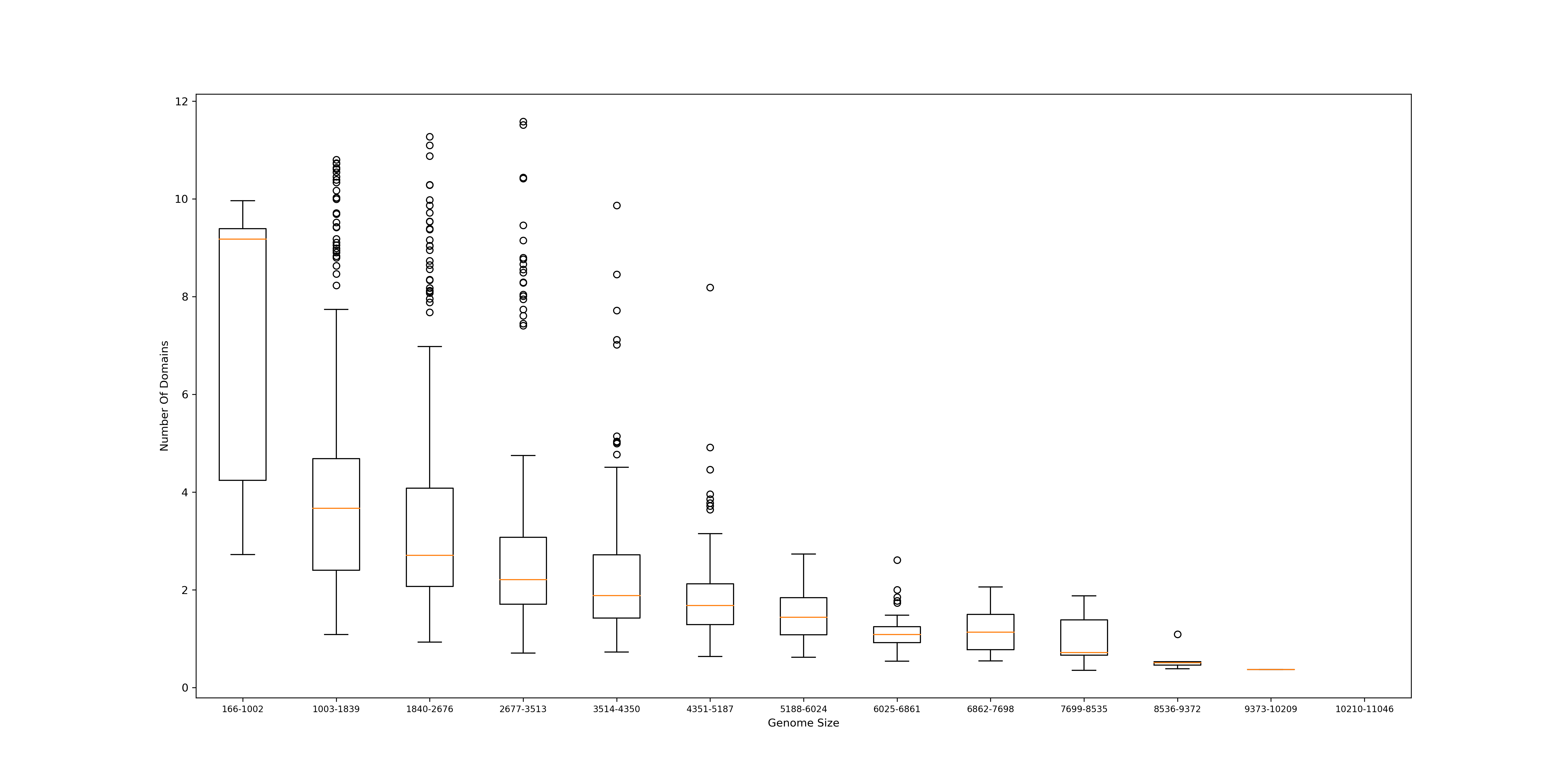

Supplement: S1 File — On the X-axis of each graph, genome size ranges are displayed in 13 windows, with a range of 836 ORFs each. On the Y-axis are the WDASs. The lines shown in the boxes are the median values. The whisker caps represent the minimum and maximum values. Superfamily IDs correspond to the names in Table 1. (ZIP) [file pone.0226604.s006.zip › Supplemnetary_material_S1/Figure_WSByIntervals_47413.png]

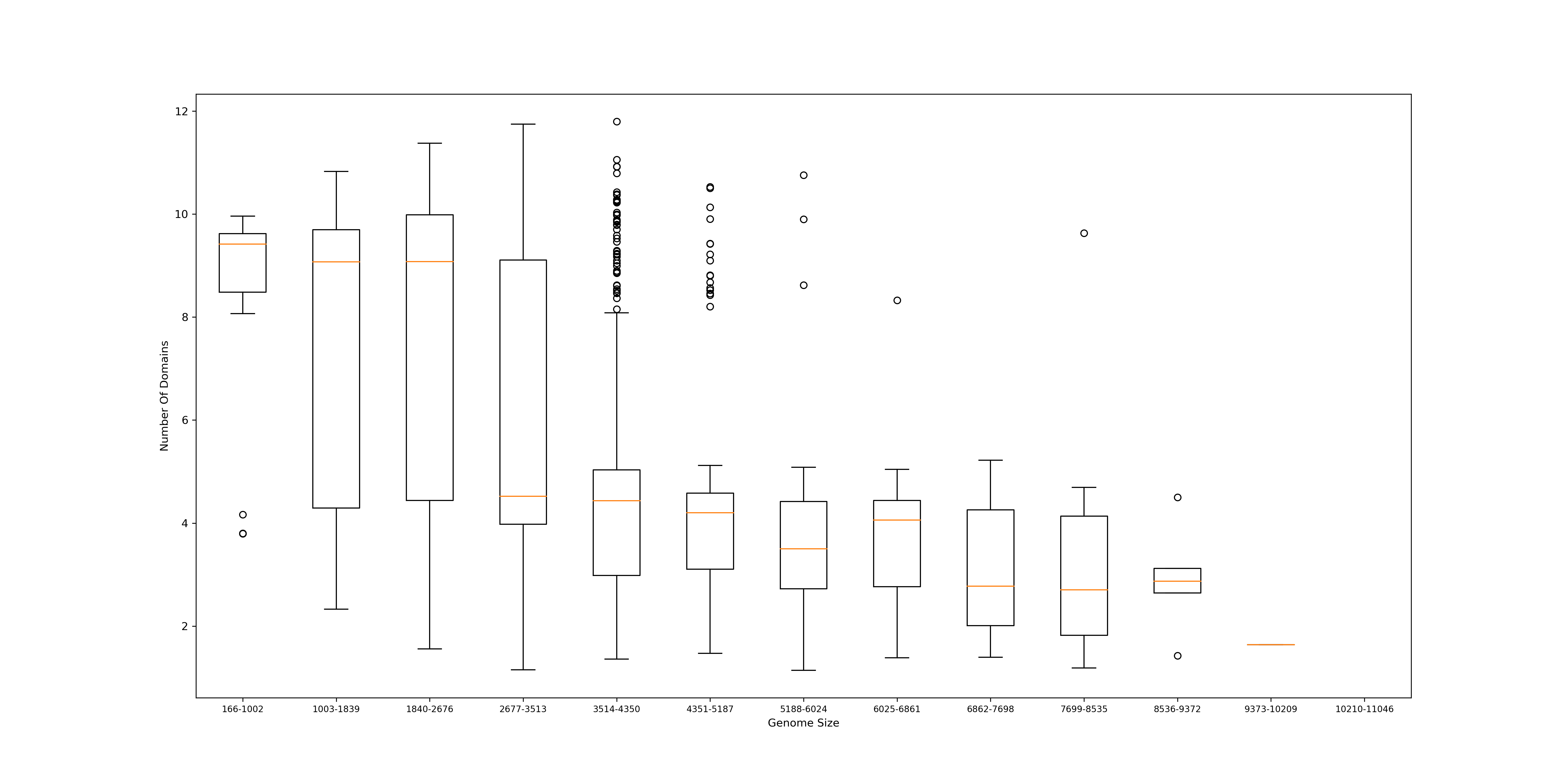

Supplement: S1 File — On the X-axis of each graph, genome size ranges are displayed in 13 windows, with a range of 836 ORFs each. On the Y-axis are the WDASs. The lines shown in the boxes are the median values. The whisker caps represent the minimum and maximum values. Superfamily IDs correspond to the names in Table 1. (ZIP) [file pone.0226604.s006.zip › Supplemnetary_material_S1/Figure_WSByIntervals_51338.png]

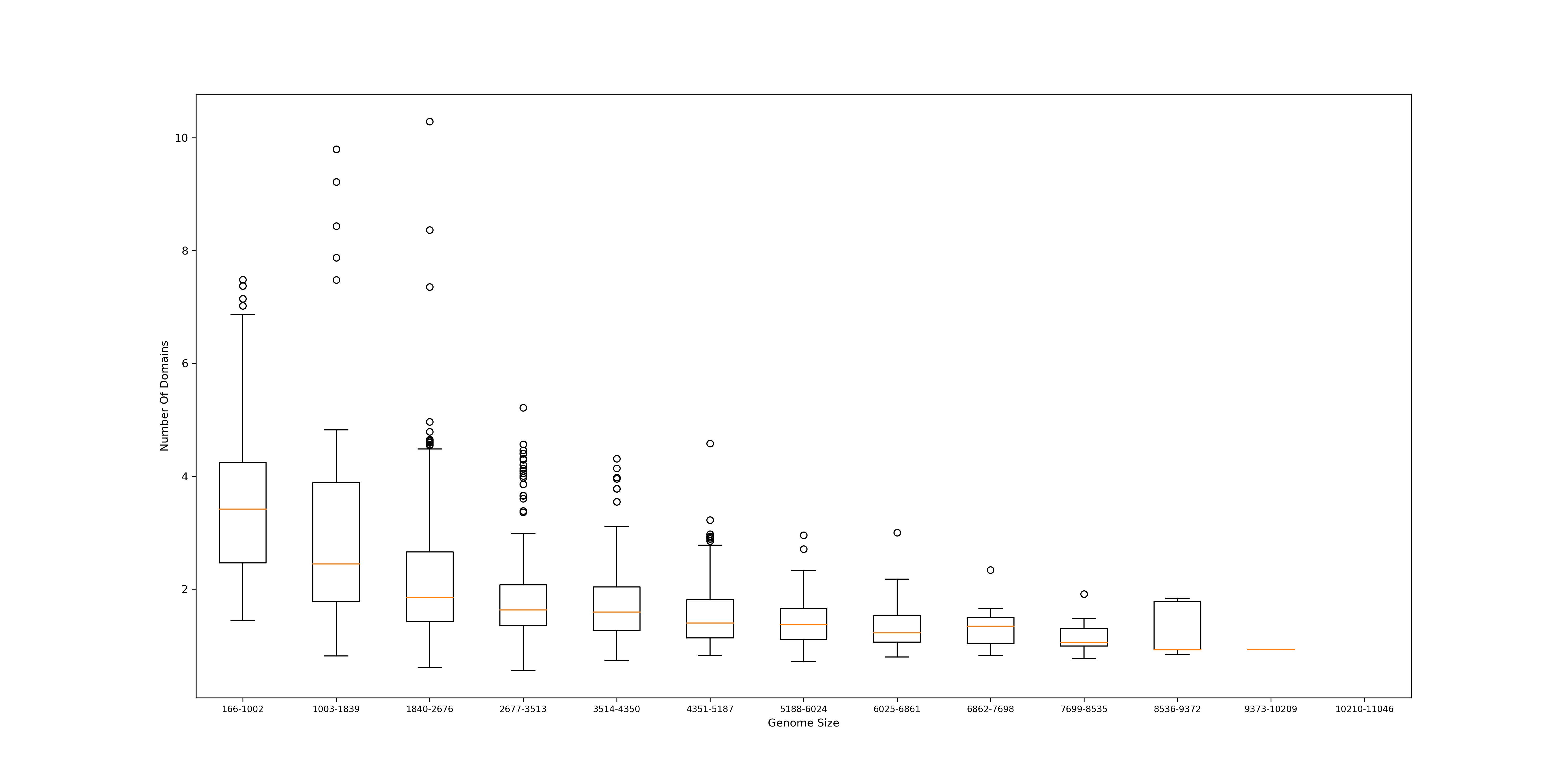

Supplement: S1 File — On the X-axis of each graph, genome size ranges are displayed in 13 windows, with a range of 836 ORFs each. On the Y-axis are the WDASs. The lines shown in the boxes are the median values. The whisker caps represent the minimum and maximum values. Superfamily IDs correspond to the names in Table 1. (ZIP) [file pone.0226604.s006.zip › Supplemnetary_material_S1/Figure_WSByIntervals_53098.png]

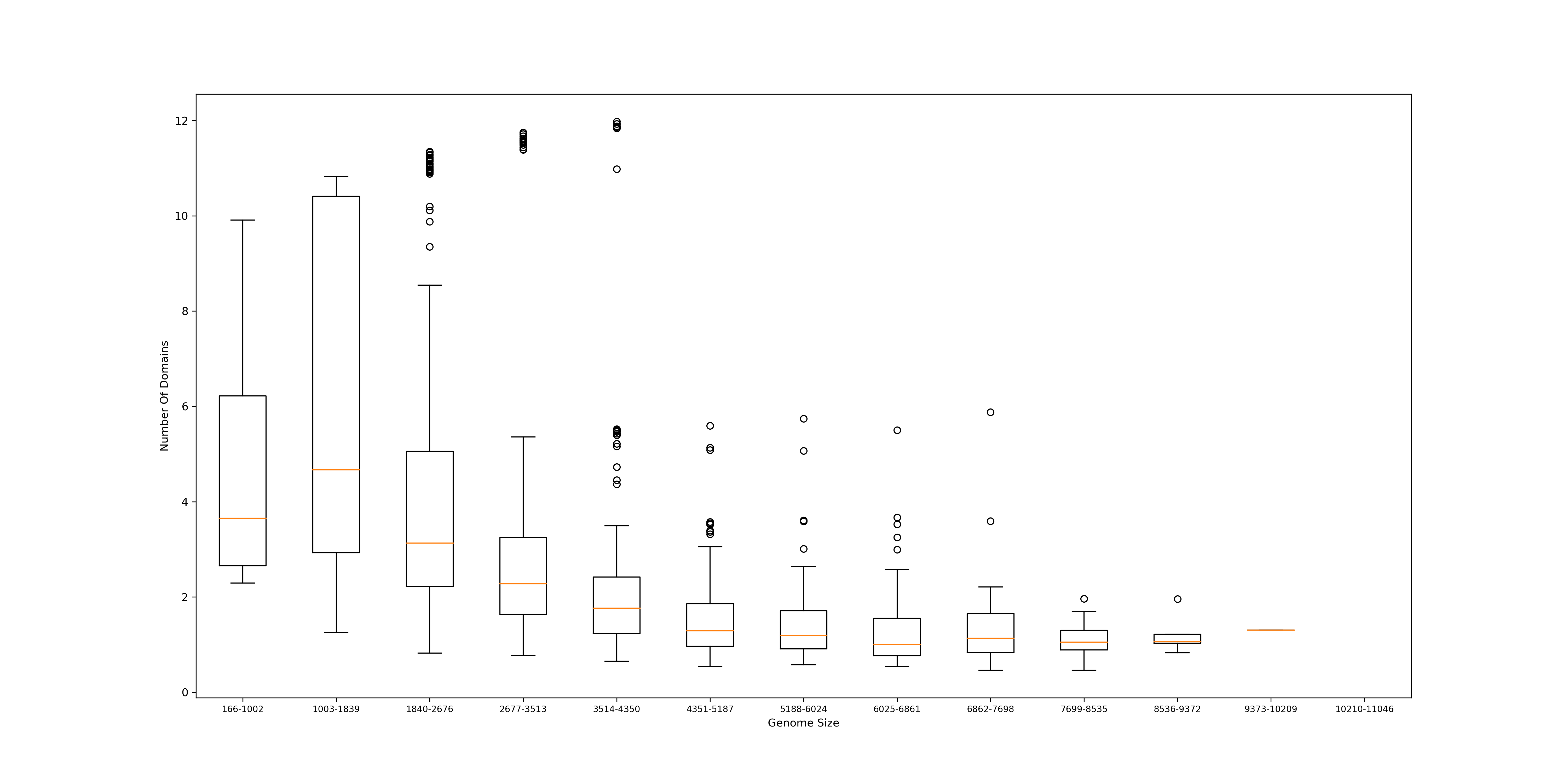

Supplement: S1 File — On the X-axis of each graph, genome size ranges are displayed in 13 windows, with a range of 836 ORFs each. On the Y-axis are the WDASs. The lines shown in the boxes are the median values. The whisker caps represent the minimum and maximum values. Superfamily IDs correspond to the names in Table 1. (ZIP) [file pone.0226604.s006.zip › Supplemnetary_material_S1/Figure_WSByIntervals_54292.png]

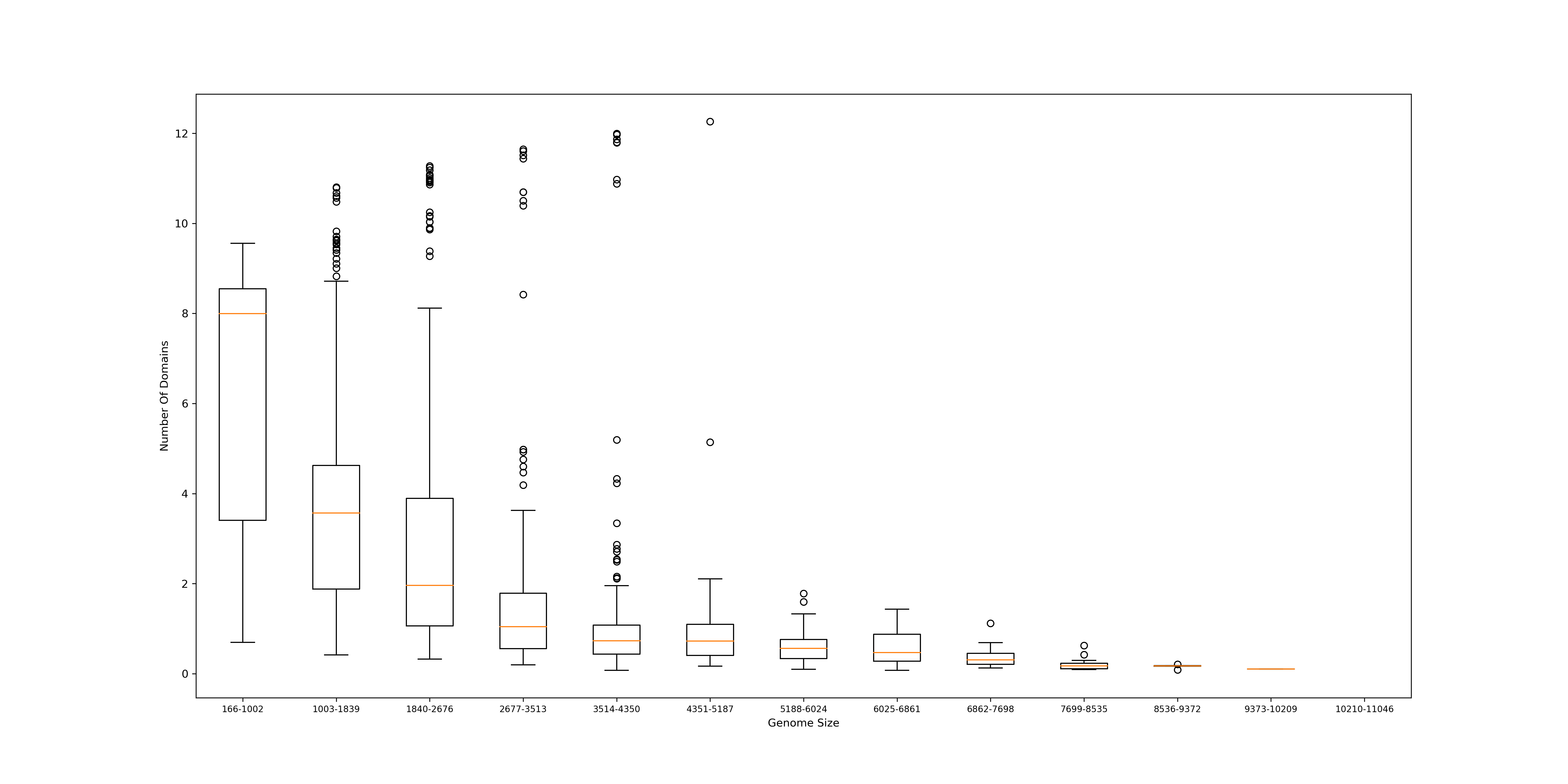

Supplement: S1 File — On the X-axis of each graph, genome size ranges are displayed in 13 windows, with a range of 836 ORFs each. On the Y-axis are the WDASs. The lines shown in the boxes are the median values. The whisker caps represent the minimum and maximum values. Superfamily IDs correspond to the names in Table 1. (ZIP) [file pone.0226604.s006.zip › Supplemnetary_material_S1/Figure_WSByIntervals_48452.png]

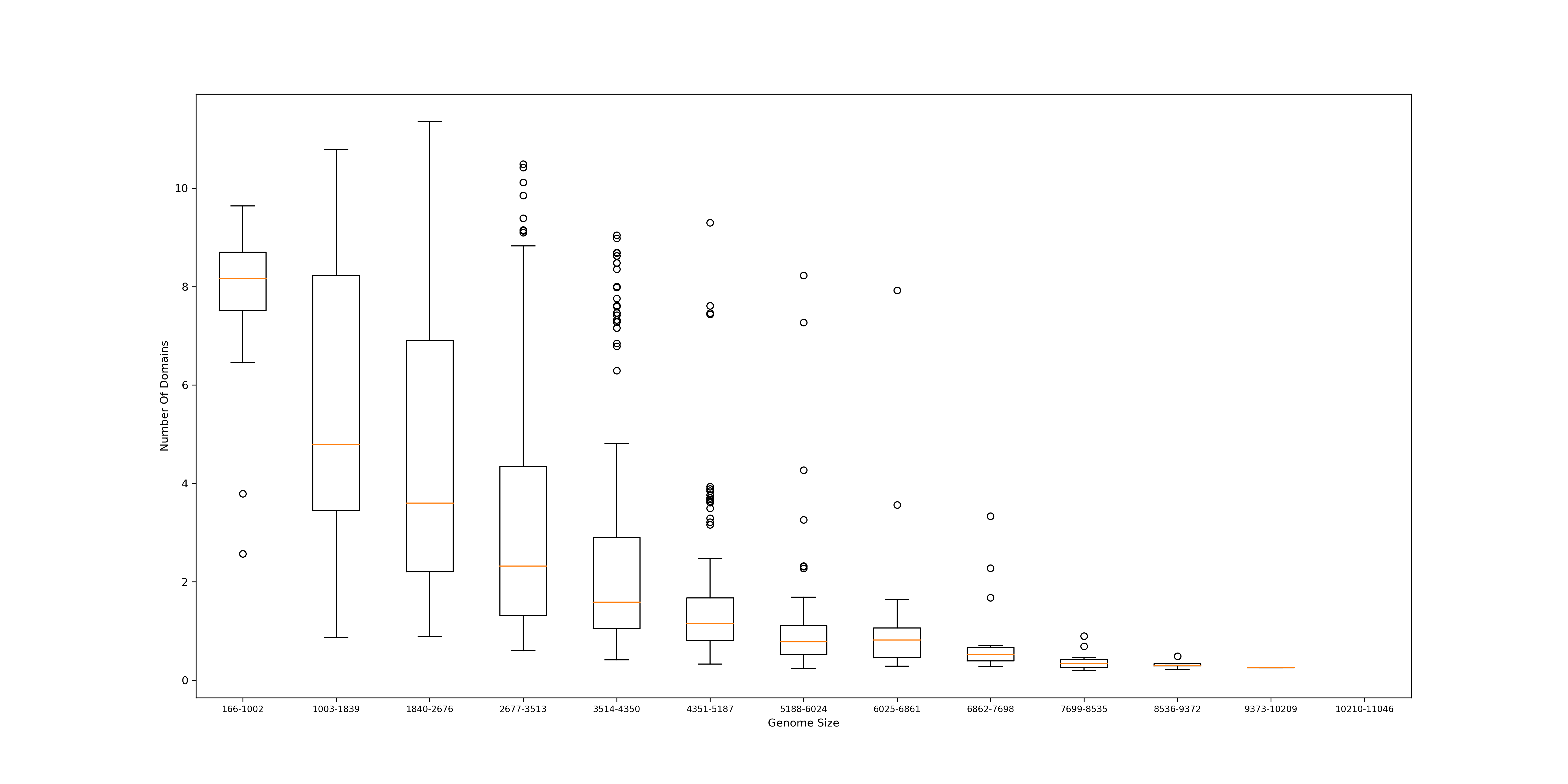

Supplement: S1 File — On the X-axis of each graph, genome size ranges are displayed in 13 windows, with a range of 836 ORFs each. On the Y-axis are the WDASs. The lines shown in the boxes are the median values. The whisker caps represent the minimum and maximum values. Superfamily IDs correspond to the names in Table 1. (ZIP) [file pone.0226604.s006.zip › Supplemnetary_material_S1/Figure_WSByIntervals_53474.png]

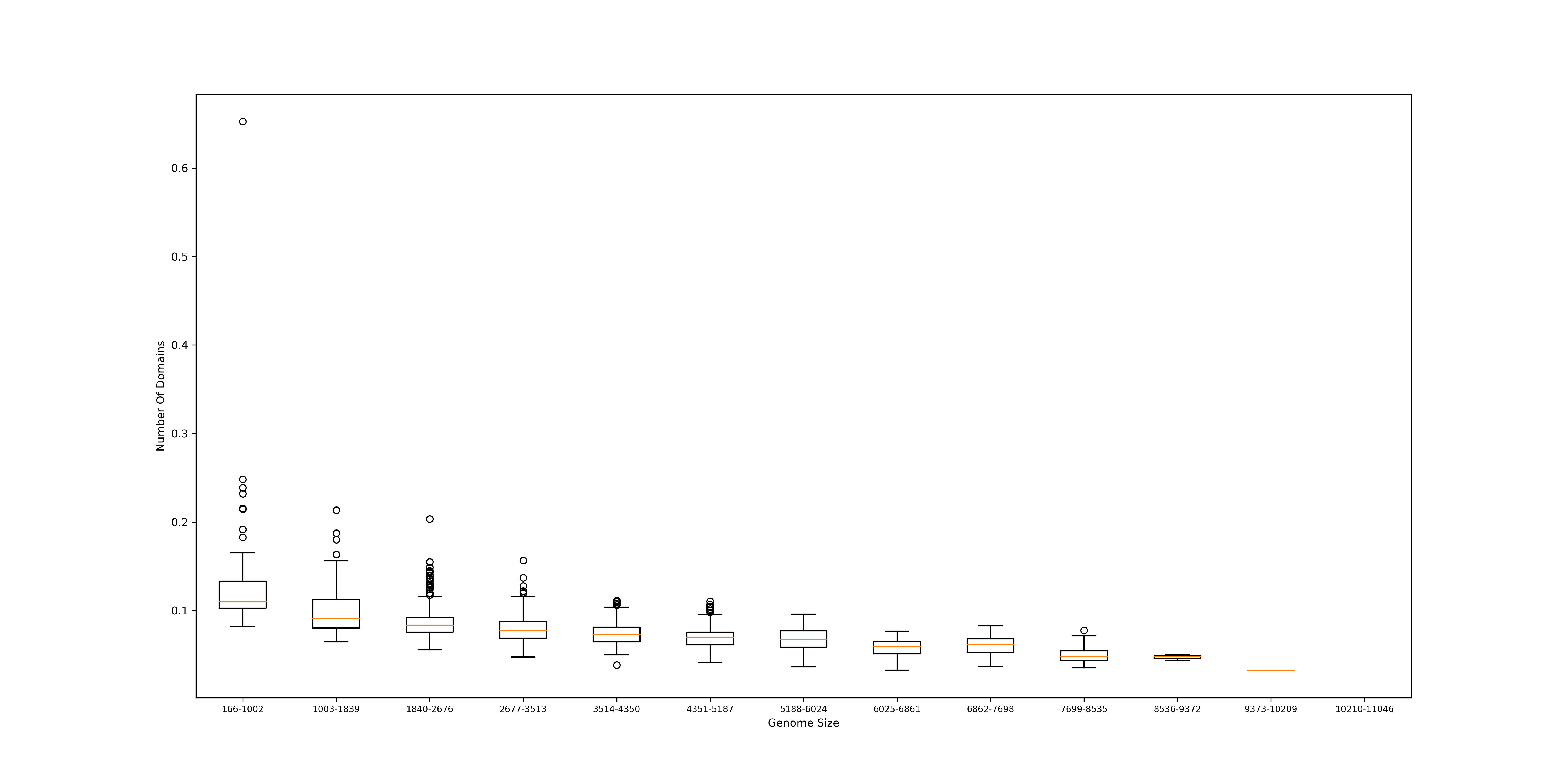

Supplement: S1 File — On the X-axis of each graph, genome size ranges are displayed in 13 windows, with a range of 836 ORFs each. On the Y-axis are the WDASs. The lines shown in the boxes are the median values. The whisker caps represent the minimum and maximum values. Superfamily IDs correspond to the names in Table 1. (ZIP) [file pone.0226604.s006.zip › Supplemnetary_material_S1/Figure_WSByIntervals_52540.png]

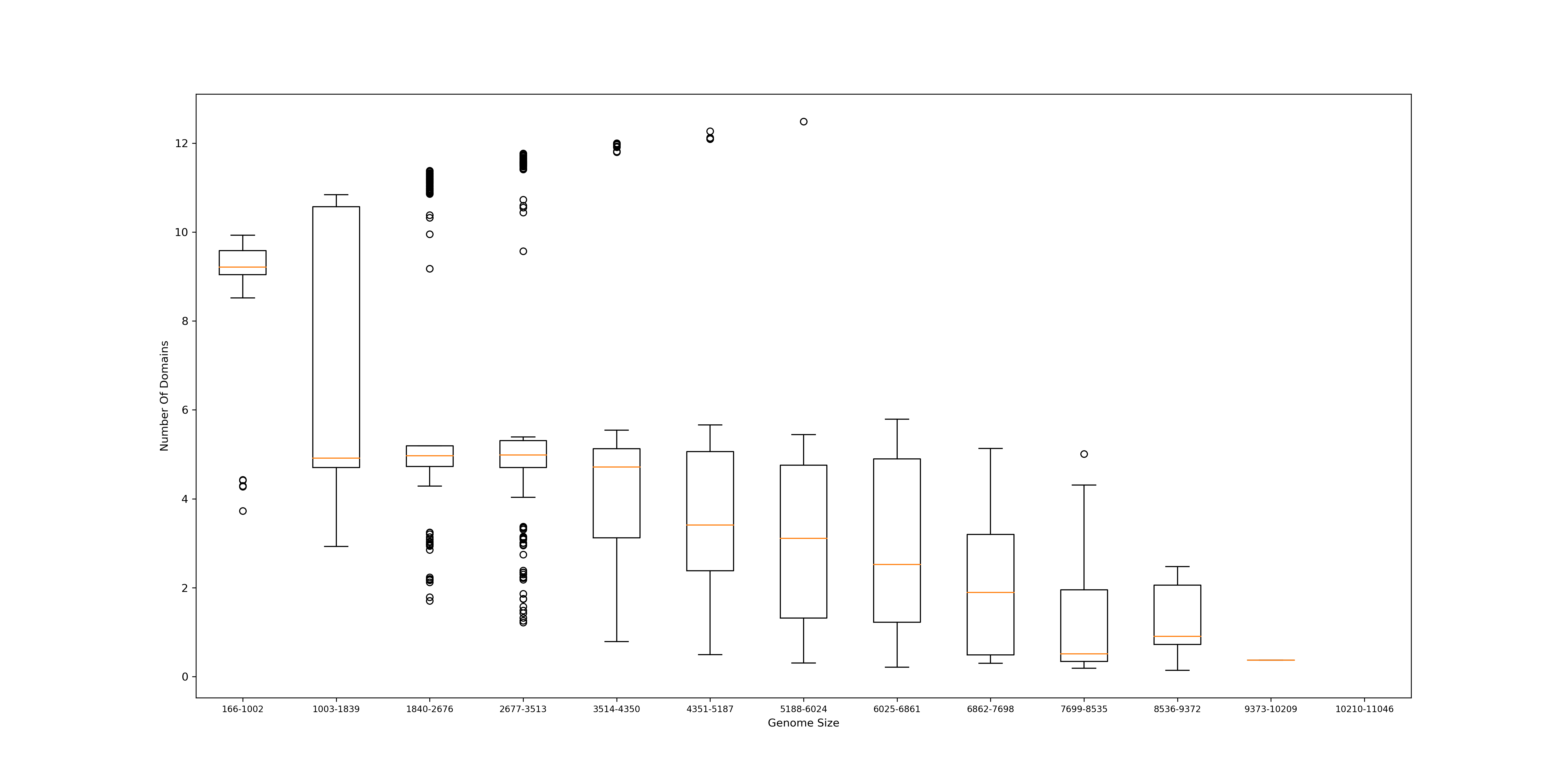

Supplement: S1 File — On the X-axis of each graph, genome size ranges are displayed in 13 windows, with a range of 836 ORFs each. On the Y-axis are the WDASs. The lines shown in the boxes are the median values. The whisker caps represent the minimum and maximum values. Superfamily IDs correspond to the names in Table 1. (ZIP) [file pone.0226604.s006.zip › Supplemnetary_material_S1/Figure_WSByIntervals_52151.png]

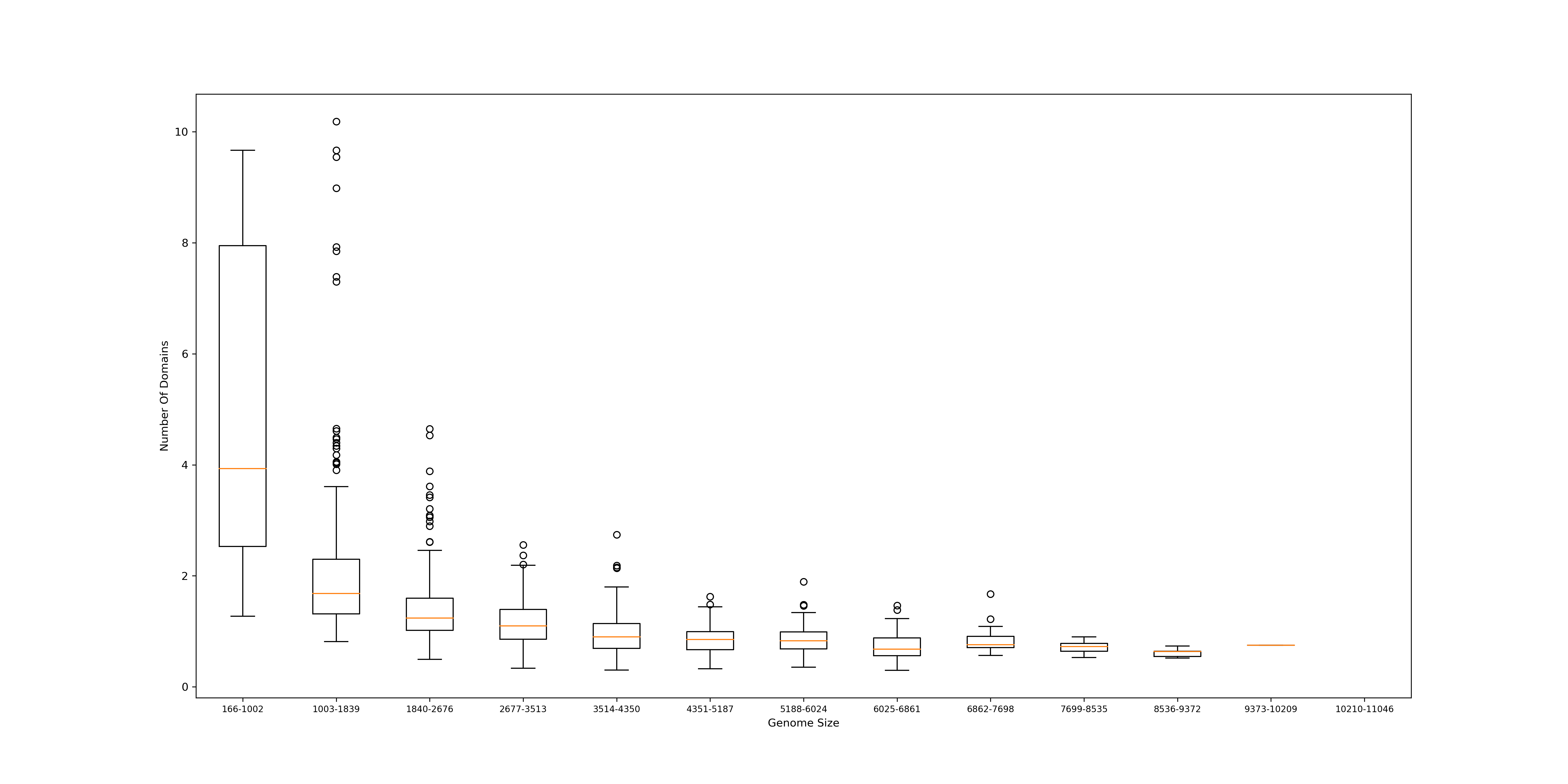

Supplement: S1 File — On the X-axis of each graph, genome size ranges are displayed in 13 windows, with a range of 836 ORFs each. On the Y-axis are the WDASs. The lines shown in the boxes are the median values. The whisker caps represent the minimum and maximum values. Superfamily IDs correspond to the names in Table 1. (ZIP) [file pone.0226604.s006.zip › Supplemnetary_material_S1/Figure_WSByIntervals_53850.png]

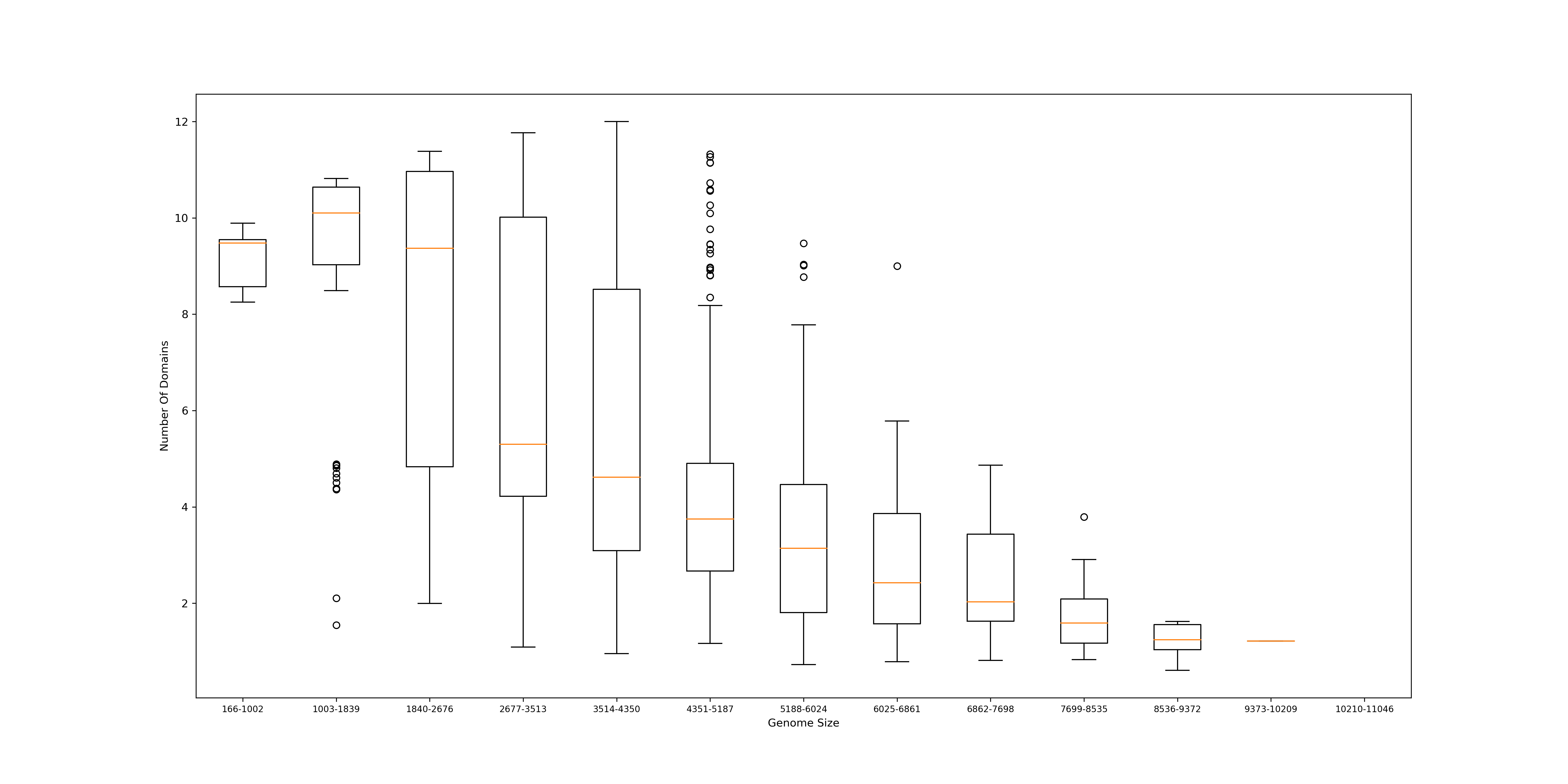

Supplement: S1 File — On the X-axis of each graph, genome size ranges are displayed in 13 windows, with a range of 836 ORFs each. On the Y-axis are the WDASs. The lines shown in the boxes are the median values. The whisker caps represent the minimum and maximum values. Superfamily IDs correspond to the names in Table 1. (ZIP) [file pone.0226604.s006.zip › Supplemnetary_material_S1/Figure_WSByIntervals_54427.png]

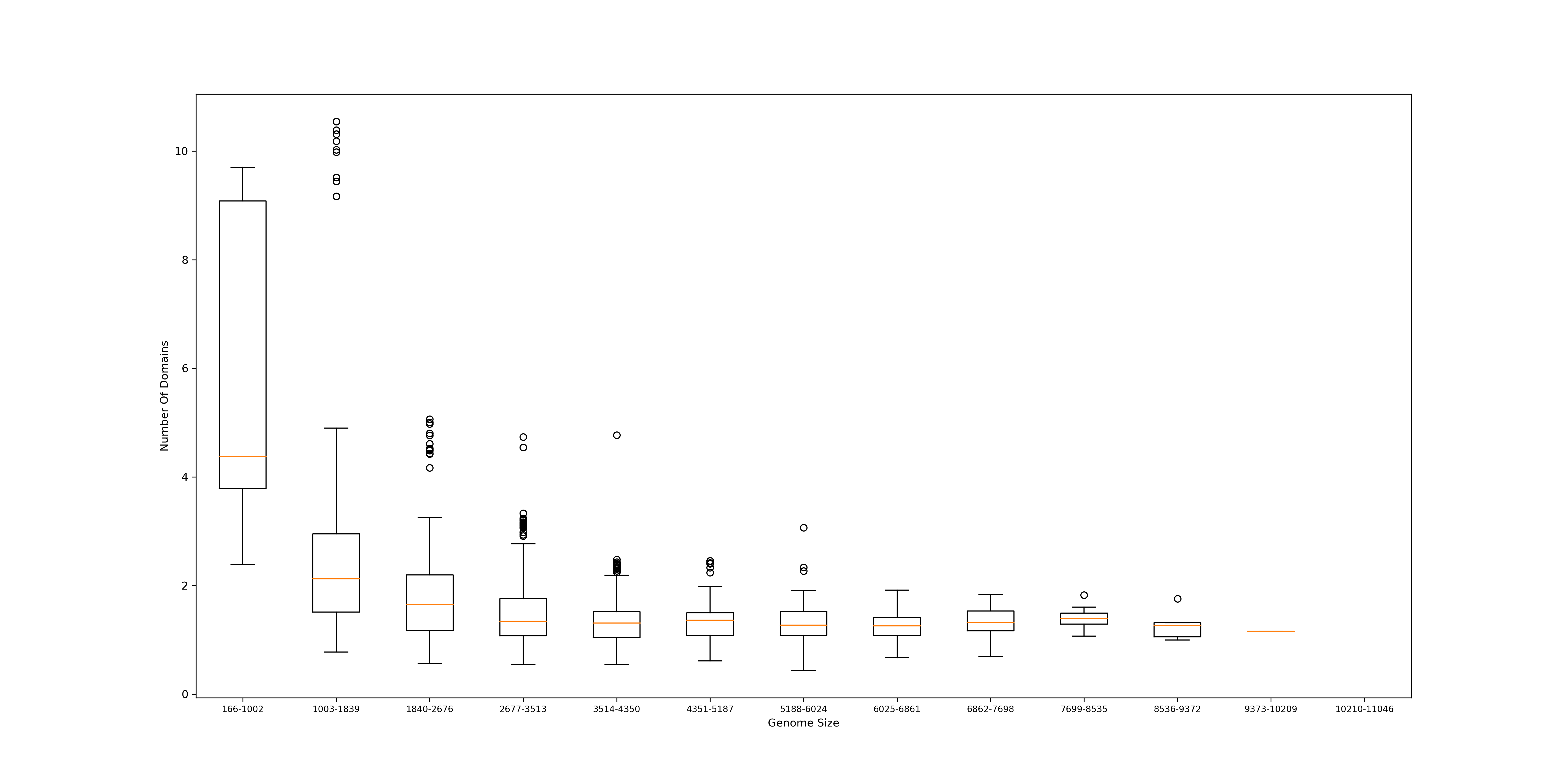

Supplement: S1 File — On the X-axis of each graph, genome size ranges are displayed in 13 windows, with a range of 836 ORFs each. On the Y-axis are the WDASs. The lines shown in the boxes are the median values. The whisker caps represent the minimum and maximum values. Superfamily IDs correspond to the names in Table 1. (ZIP) [file pone.0226604.s006.zip › Supplemnetary_material_S1/Figure_WSByIntervals_54631.png]

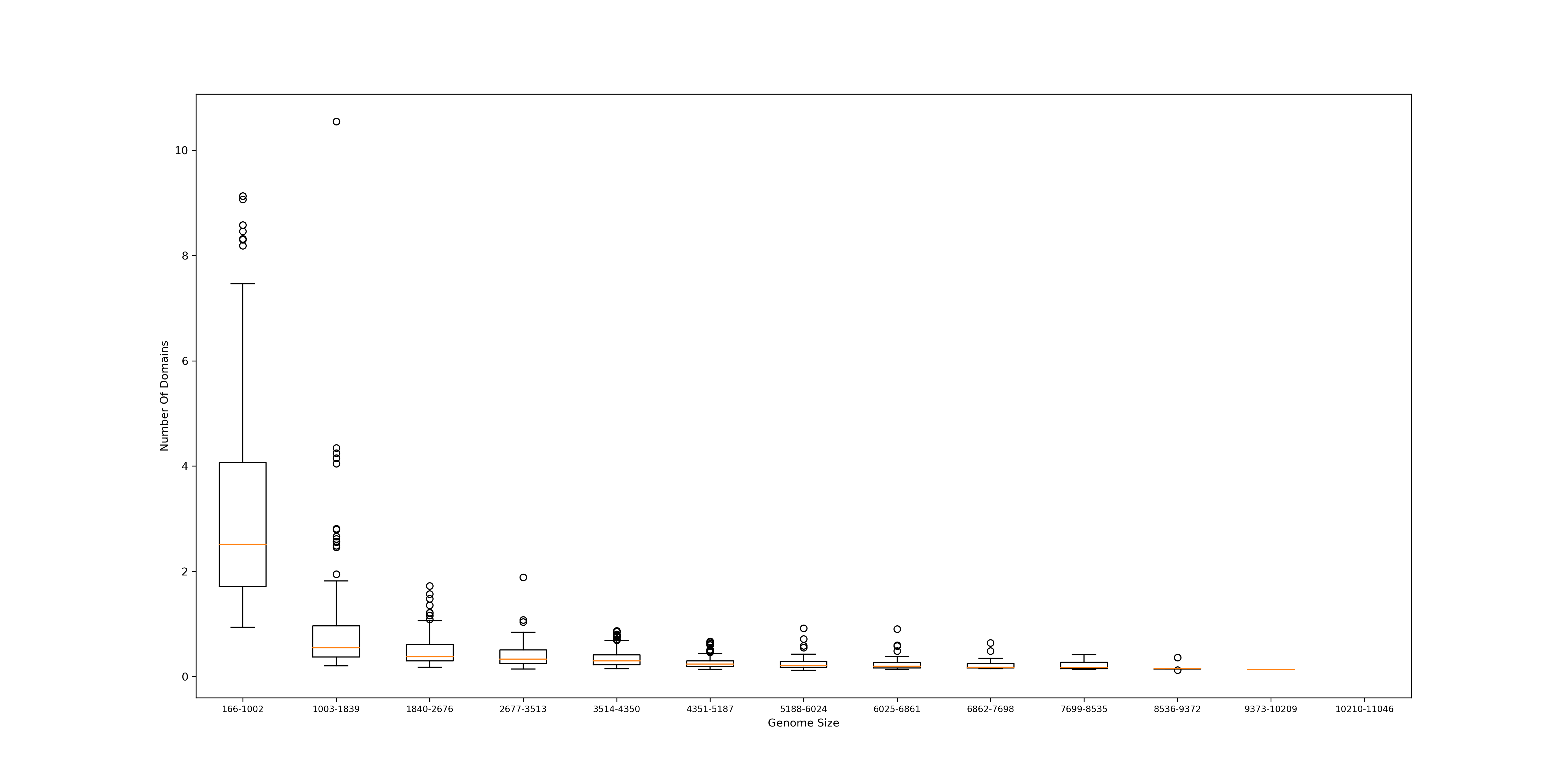

Supplement: S1 File — On the X-axis of each graph, genome size ranges are displayed in 13 windows, with a range of 836 ORFs each. On the Y-axis are the WDASs. The lines shown in the boxes are the median values. The whisker caps represent the minimum and maximum values. Superfamily IDs correspond to the names in Table 1. (ZIP) [file pone.0226604.s006.zip › Supplemnetary_material_S1/Figure_WSByIntervals_46785.png]

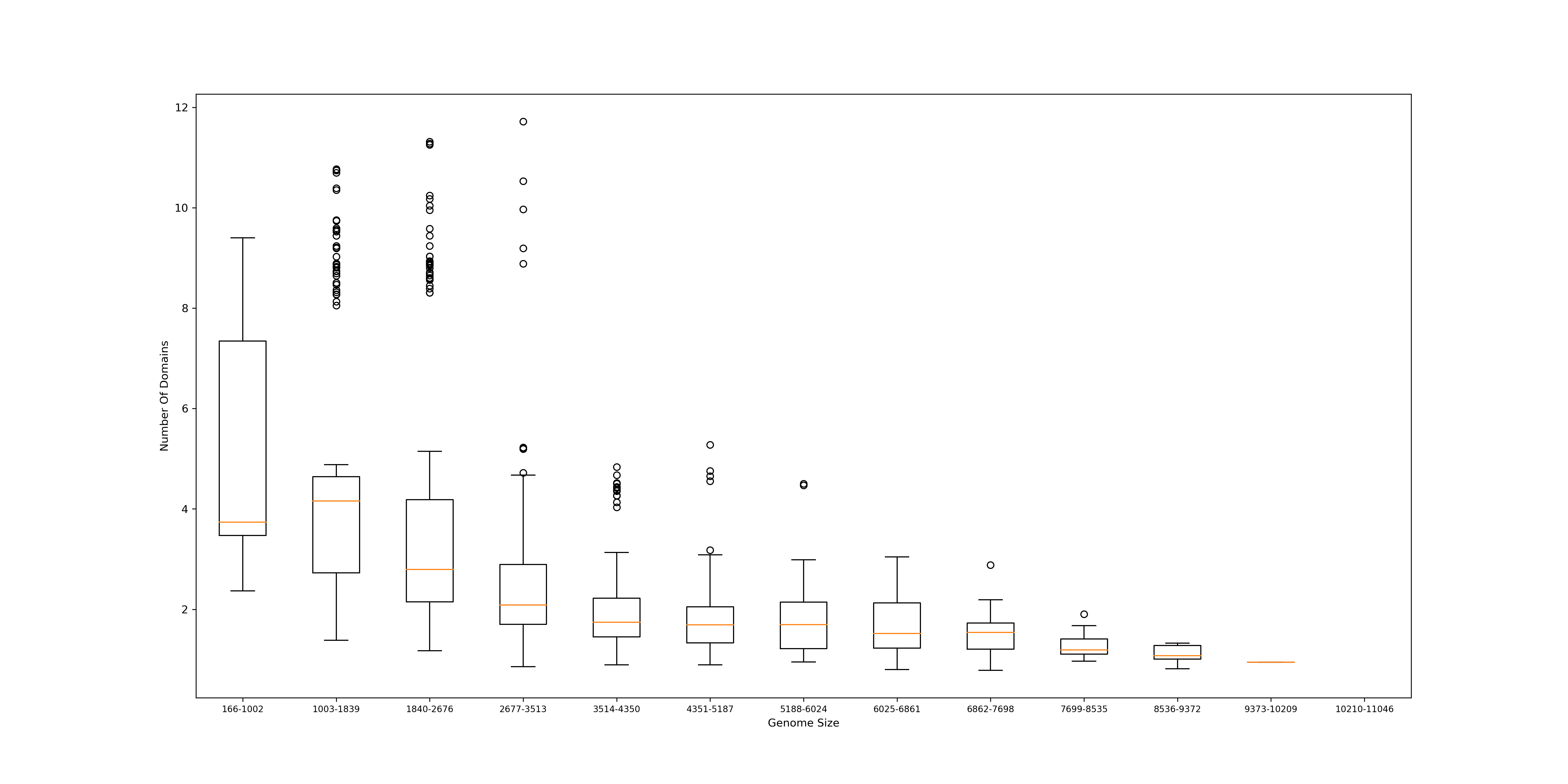

Supplement: S1 File — On the X-axis of each graph, genome size ranges are displayed in 13 windows, with a range of 836 ORFs each. On the Y-axis are the WDASs. The lines shown in the boxes are the median values. The whisker caps represent the minimum and maximum values. Superfamily IDs correspond to the names in Table 1. (ZIP) [file pone.0226604.s006.zip › Supplemnetary_material_S1/Figure_WSByIntervals_52096.png]

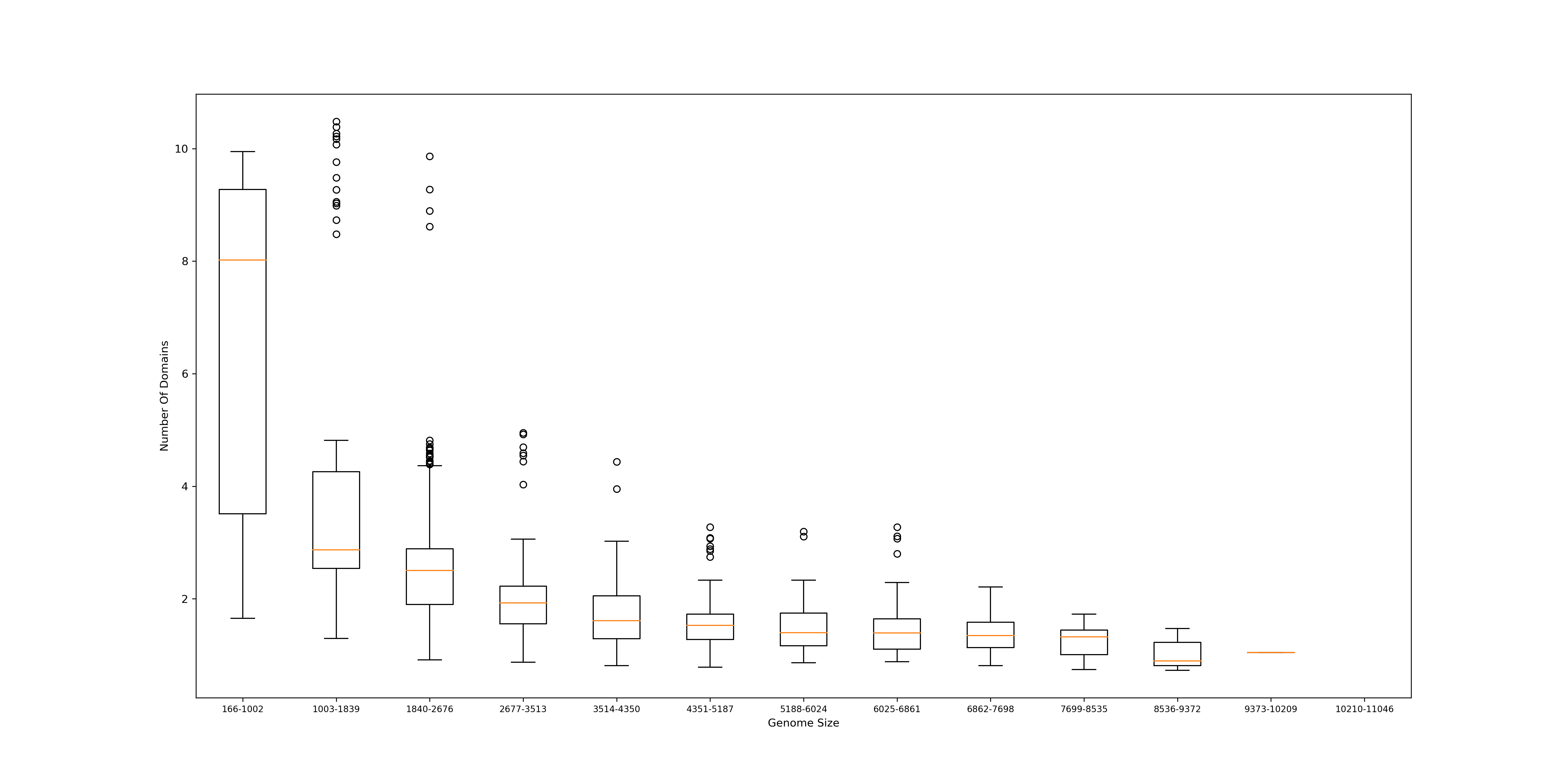

Supplement: S1 File — On the X-axis of each graph, genome size ranges are displayed in 13 windows, with a range of 836 ORFs each. On the Y-axis are the WDASs. The lines shown in the boxes are the median values. The whisker caps represent the minimum and maximum values. Superfamily IDs correspond to the names in Table 1. (ZIP) [file pone.0226604.s006.zip › Supplemnetary_material_S1/Figure_WSByIntervals_48179.png]

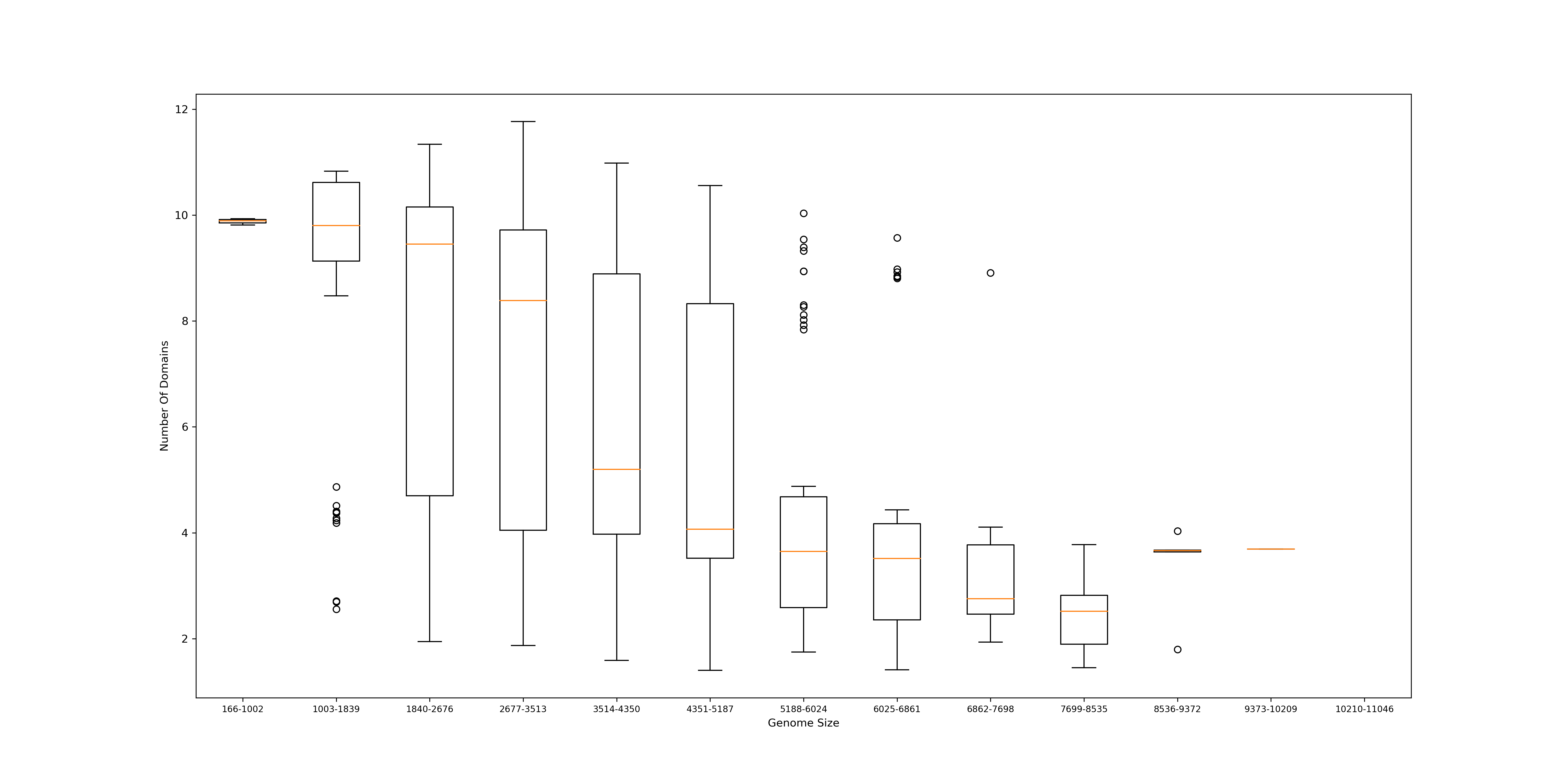

Supplement: S1 File — On the X-axis of each graph, genome size ranges are displayed in 13 windows, with a range of 836 ORFs each. On the Y-axis are the WDASs. The lines shown in the boxes are the median values. The whisker caps represent the minimum and maximum values. Superfamily IDs correspond to the names in Table 1. (ZIP) [file pone.0226604.s006.zip › Supplemnetary_material_S1/Figure_WSByIntervals_54593.png]

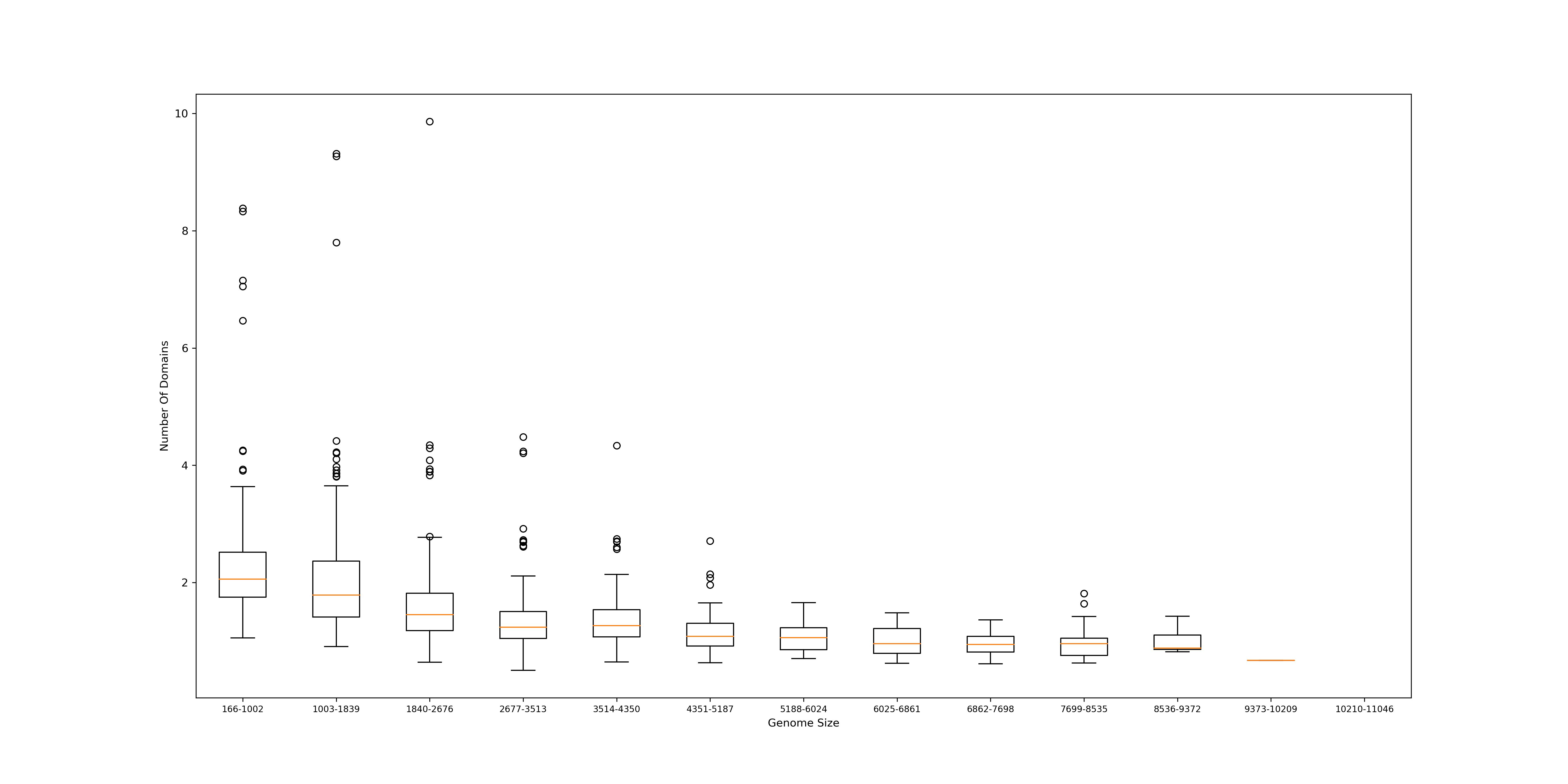

Supplement: S1 File — On the X-axis of each graph, genome size ranges are displayed in 13 windows, with a range of 836 ORFs each. On the Y-axis are the WDASs. The lines shown in the boxes are the median values. The whisker caps represent the minimum and maximum values. Superfamily IDs correspond to the names in Table 1. (ZIP) [file pone.0226604.s006.zip › Supplemnetary_material_S1/Figure_WSByIntervals_52518.png]

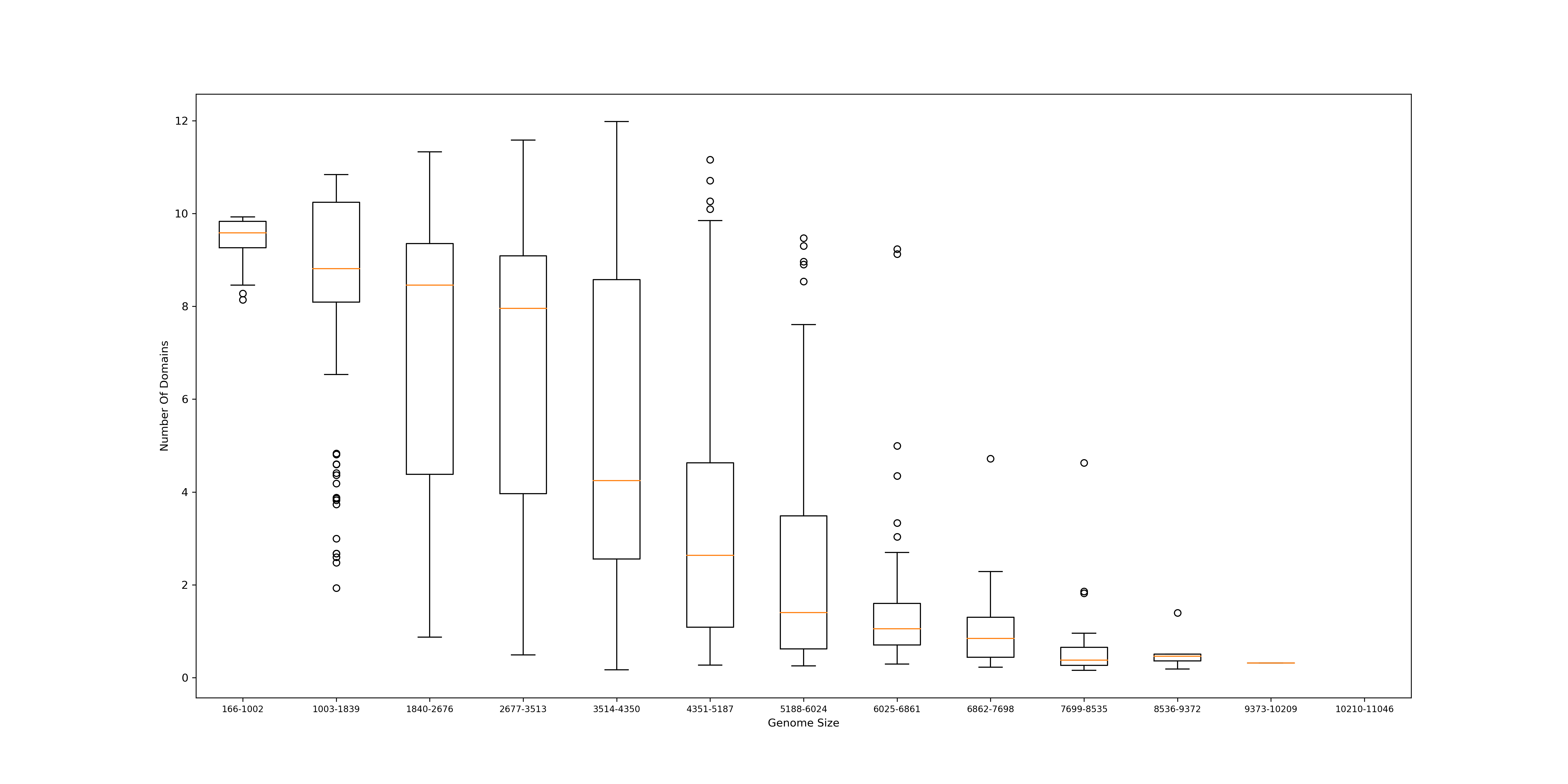

Supplement: S1 File — On the X-axis of each graph, genome size ranges are displayed in 13 windows, with a range of 836 ORFs each. On the Y-axis are the WDASs. The lines shown in the boxes are the median values. The whisker caps represent the minimum and maximum values. Superfamily IDs correspond to the names in Table 1. (ZIP) [file pone.0226604.s006.zip › Supplemnetary_material_S1/Figure_WSByIntervals_56801.png]

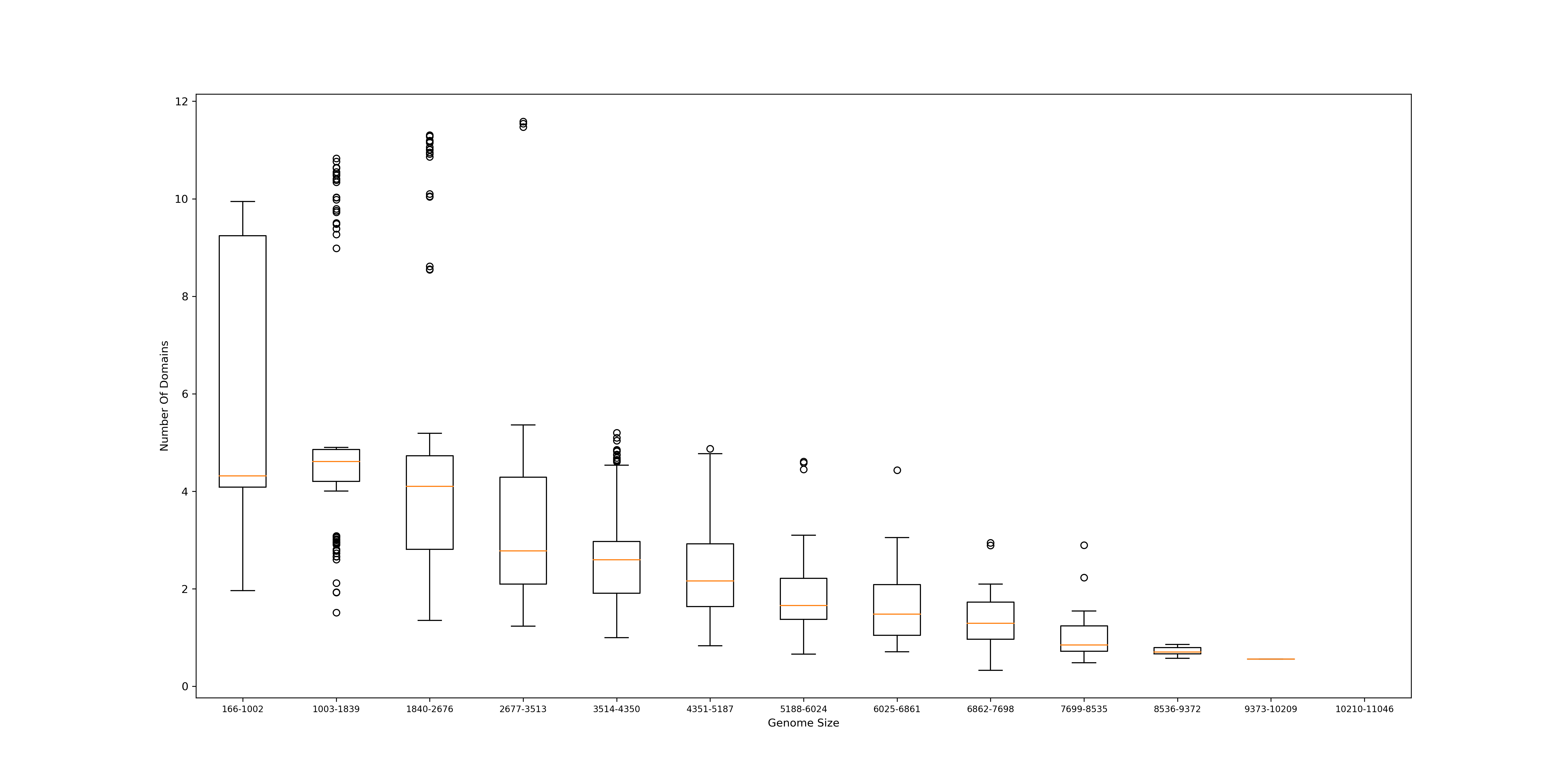

Supplement: S1 File — On the X-axis of each graph, genome size ranges are displayed in 13 windows, with a range of 836 ORFs each. On the Y-axis are the WDASs. The lines shown in the boxes are the median values. The whisker caps represent the minimum and maximum values. Superfamily IDs correspond to the names in Table 1. (ZIP) [file pone.0226604.s006.zip › Supplemnetary_material_S1/Figure_WSByIntervals_88946.png]

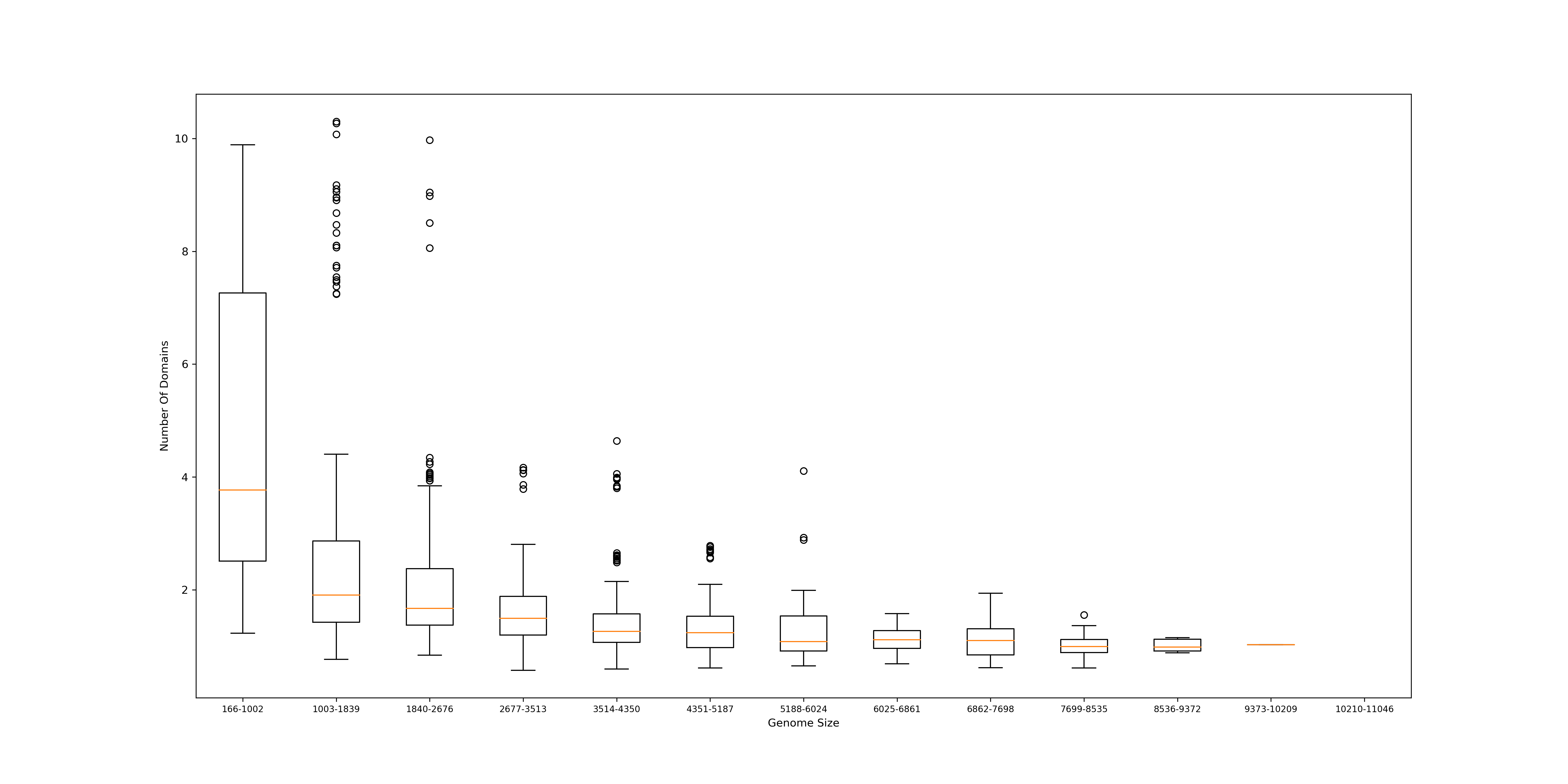

Supplement: S1 File — On the X-axis of each graph, genome size ranges are displayed in 13 windows, with a range of 836 ORFs each. On the Y-axis are the WDASs. The lines shown in the boxes are the median values. The whisker caps represent the minimum and maximum values. Superfamily IDs correspond to the names in Table 1. (ZIP) [file pone.0226604.s006.zip › Supplemnetary_material_S1/Figure_WSByIntervals_56784.png]

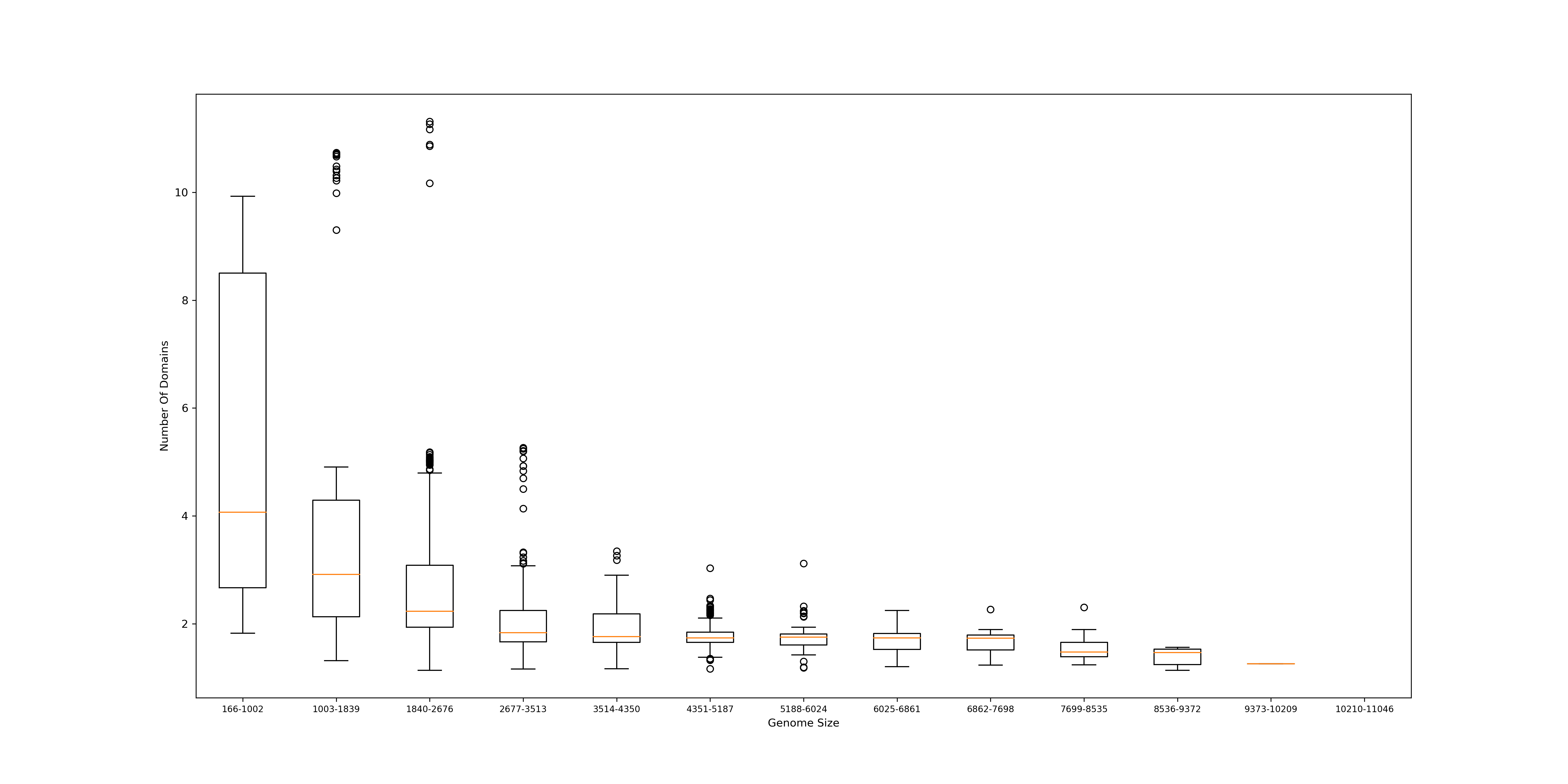

Supplement: S1 File — On the X-axis of each graph, genome size ranges are displayed in 13 windows, with a range of 836 ORFs each. On the Y-axis are the WDASs. The lines shown in the boxes are the median values. The whisker caps represent the minimum and maximum values. Superfamily IDs correspond to the names in Table 1. (ZIP) [file pone.0226604.s006.zip › Supplemnetary_material_S1/Figure_WSByIntervals_56235.png]

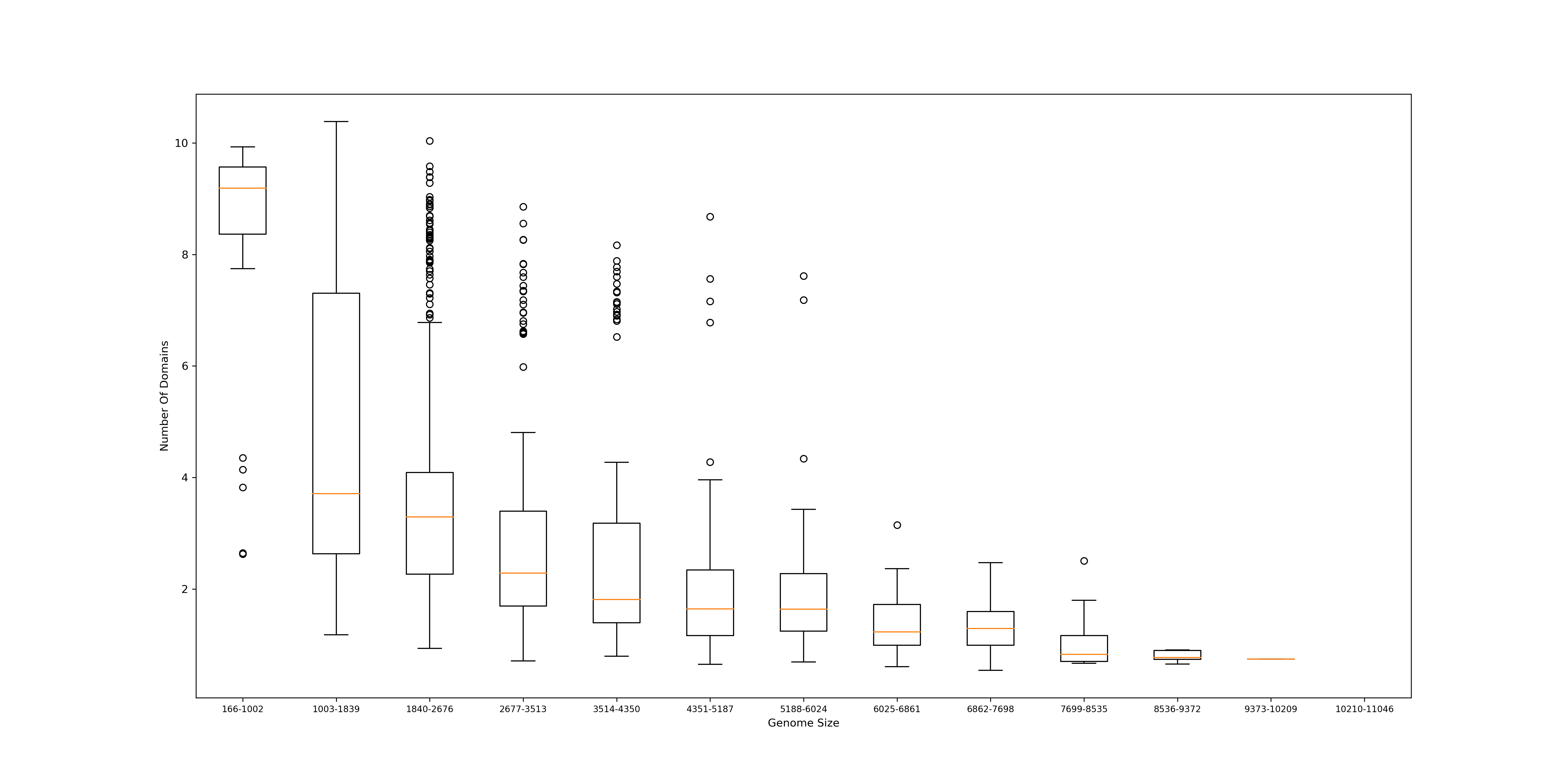

Supplement: S1 File — On the X-axis of each graph, genome size ranges are displayed in 13 windows, with a range of 836 ORFs each. On the Y-axis are the WDASs. The lines shown in the boxes are the median values. The whisker caps represent the minimum and maximum values. Superfamily IDs correspond to the names in Table 1. (ZIP) [file pone.0226604.s006.zip › Supplemnetary_material_S1/Figure_WSByIntervals_55729.png]

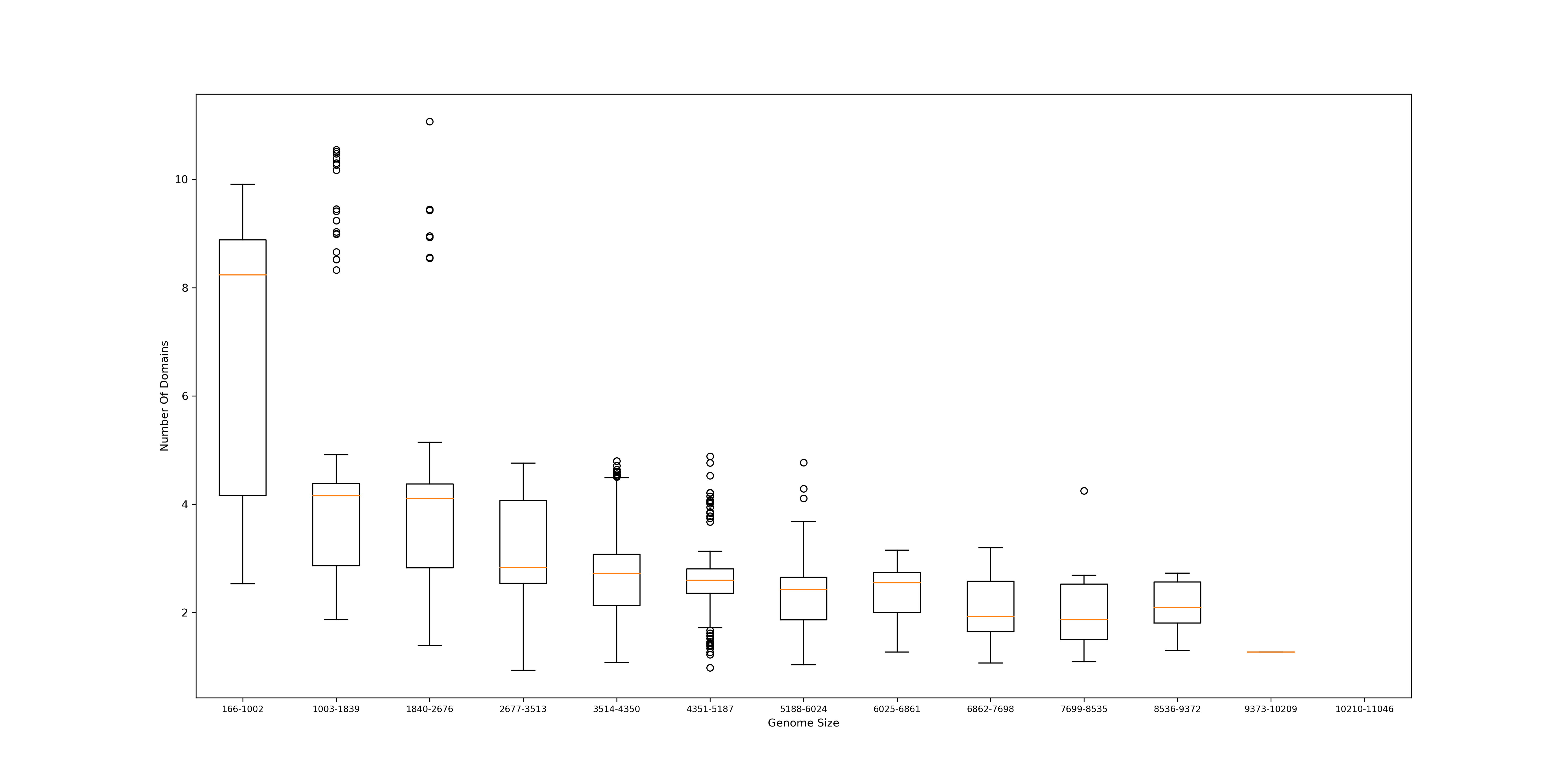

Supplement: S1 File — On the X-axis of each graph, genome size ranges are displayed in 13 windows, with a range of 836 ORFs each. On the Y-axis are the WDASs. The lines shown in the boxes are the median values. The whisker caps represent the minimum and maximum values. Superfamily IDs correspond to the names in Table 1. (ZIP) [file pone.0226604.s006.zip › Supplemnetary_material_S1/Figure_WSByIntervals_51556.png]

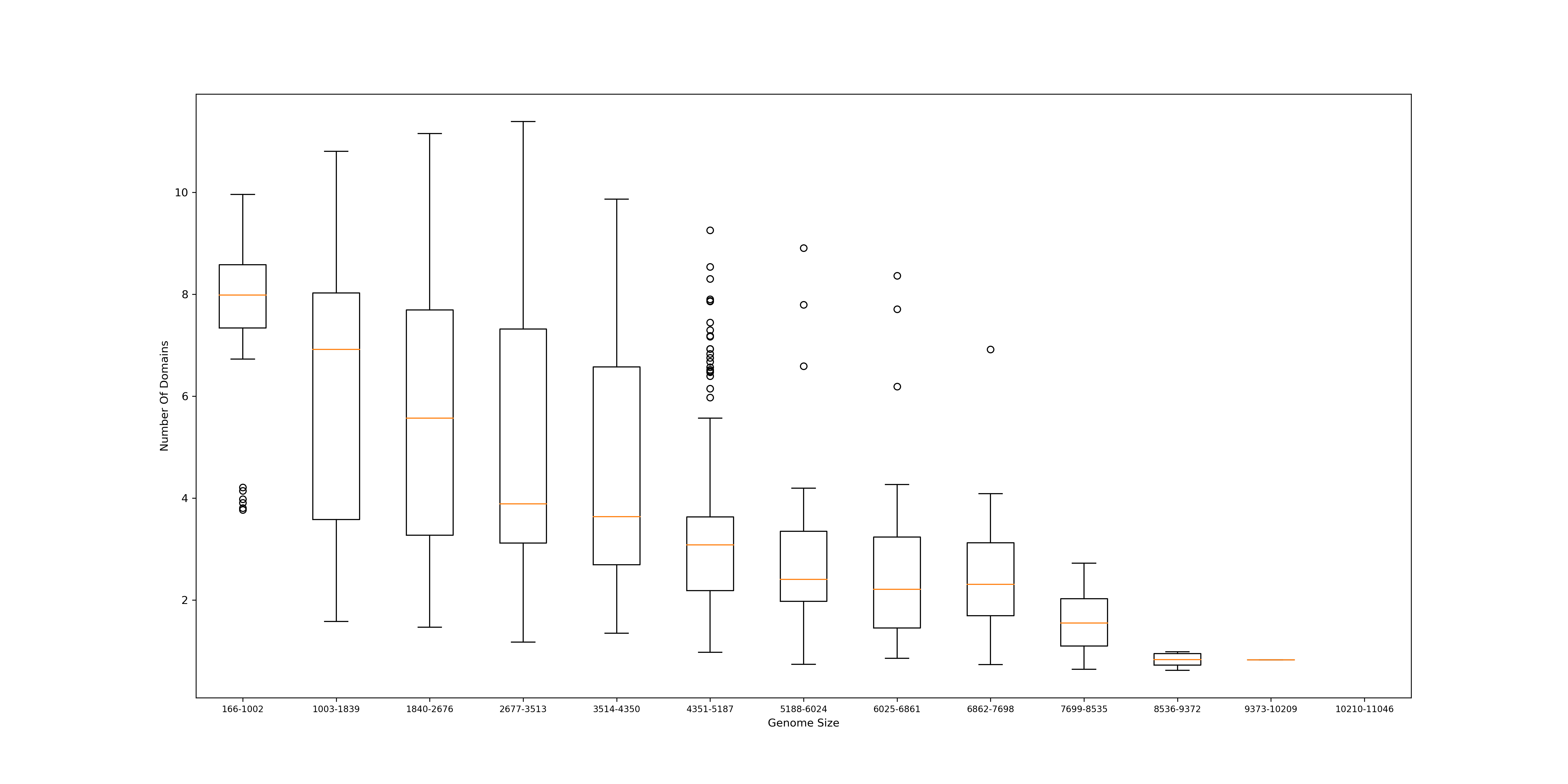

Supplement: S1 File — On the X-axis of each graph, genome size ranges are displayed in 13 windows, with a range of 836 ORFs each. On the Y-axis are the WDASs. The lines shown in the boxes are the median values. The whisker caps represent the minimum and maximum values. Superfamily IDs correspond to the names in Table 1. (ZIP) [file pone.0226604.s006.zip › Supplemnetary_material_S1/Figure_WSByIntervals_103473.png]

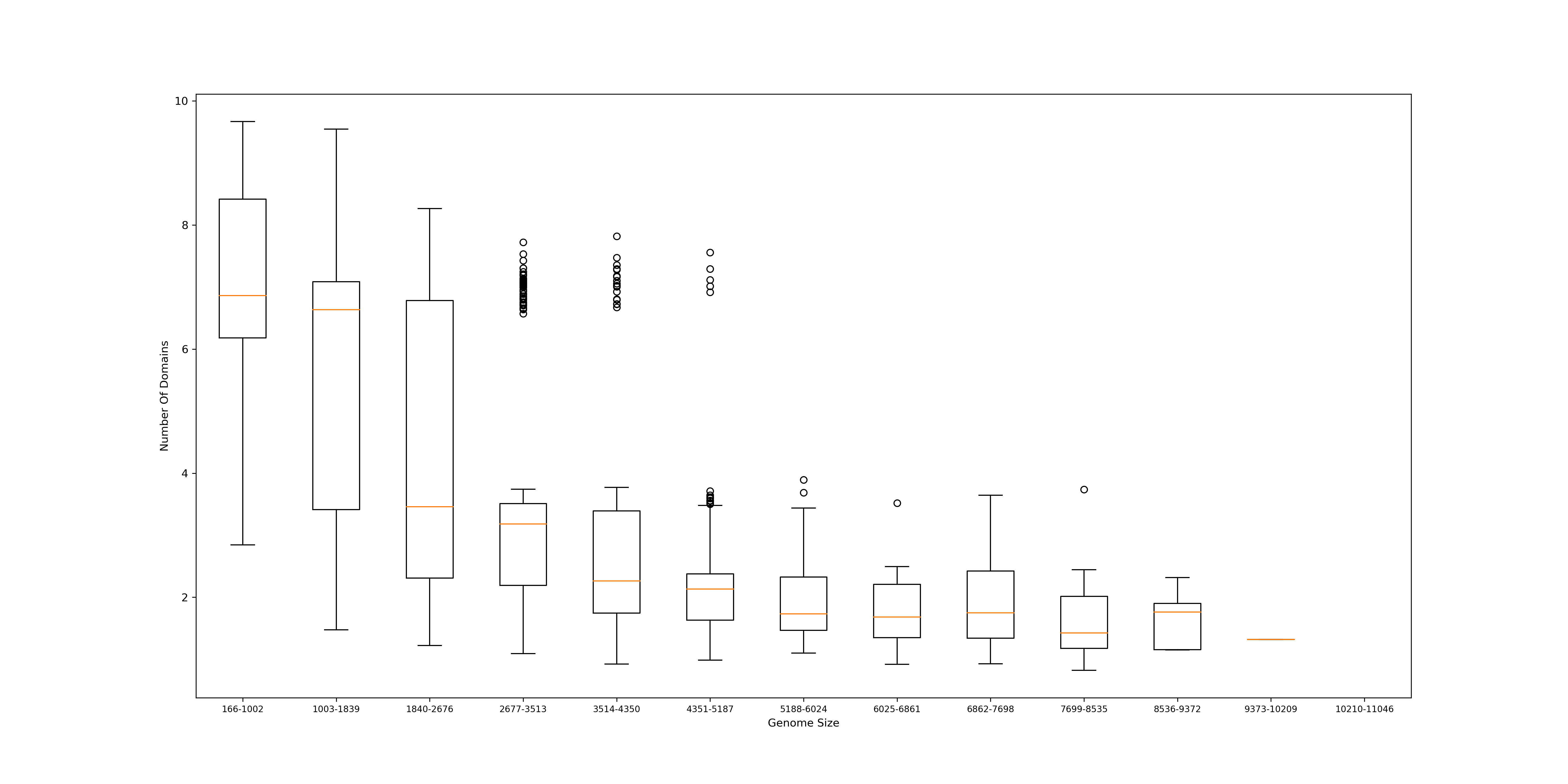

Supplement: S1 File — On the X-axis of each graph, genome size ranges are displayed in 13 windows, with a range of 836 ORFs each. On the Y-axis are the WDASs. The lines shown in the boxes are the median values. The whisker caps represent the minimum and maximum values. Superfamily IDs correspond to the names in Table 1. (ZIP) [file pone.0226604.s006.zip › Supplemnetary_material_S1/Figure_WSByIntervals_53383.png]

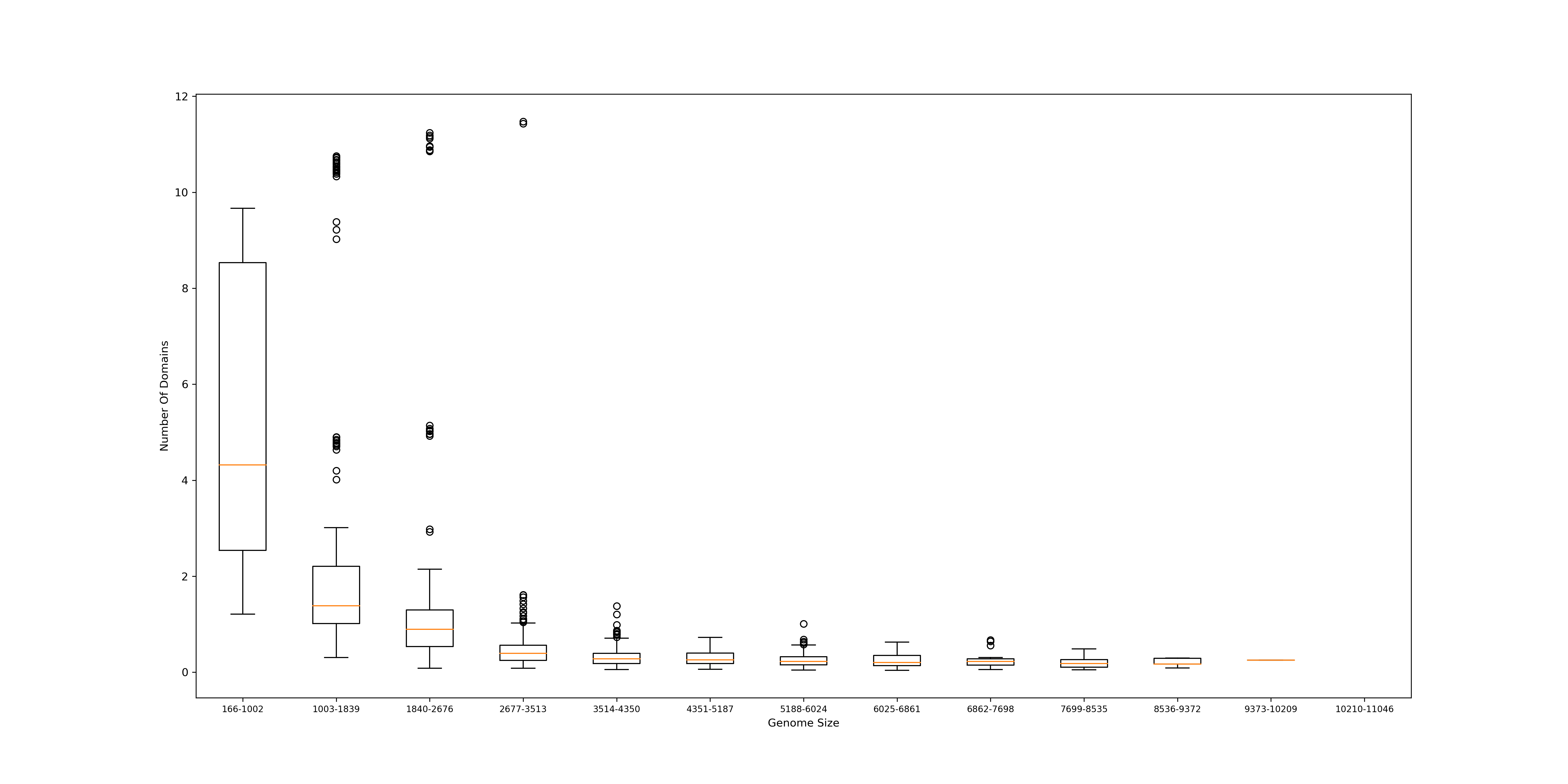

Supplement: S1 File — On the X-axis of each graph, genome size ranges are displayed in 13 windows, with a range of 836 ORFs each. On the Y-axis are the WDASs. The lines shown in the boxes are the median values. The whisker caps represent the minimum and maximum values. Superfamily IDs correspond to the names in Table 1. (ZIP) [file pone.0226604.s006.zip › Supplemnetary_material_S1/Figure_WSByIntervals_55874.png]

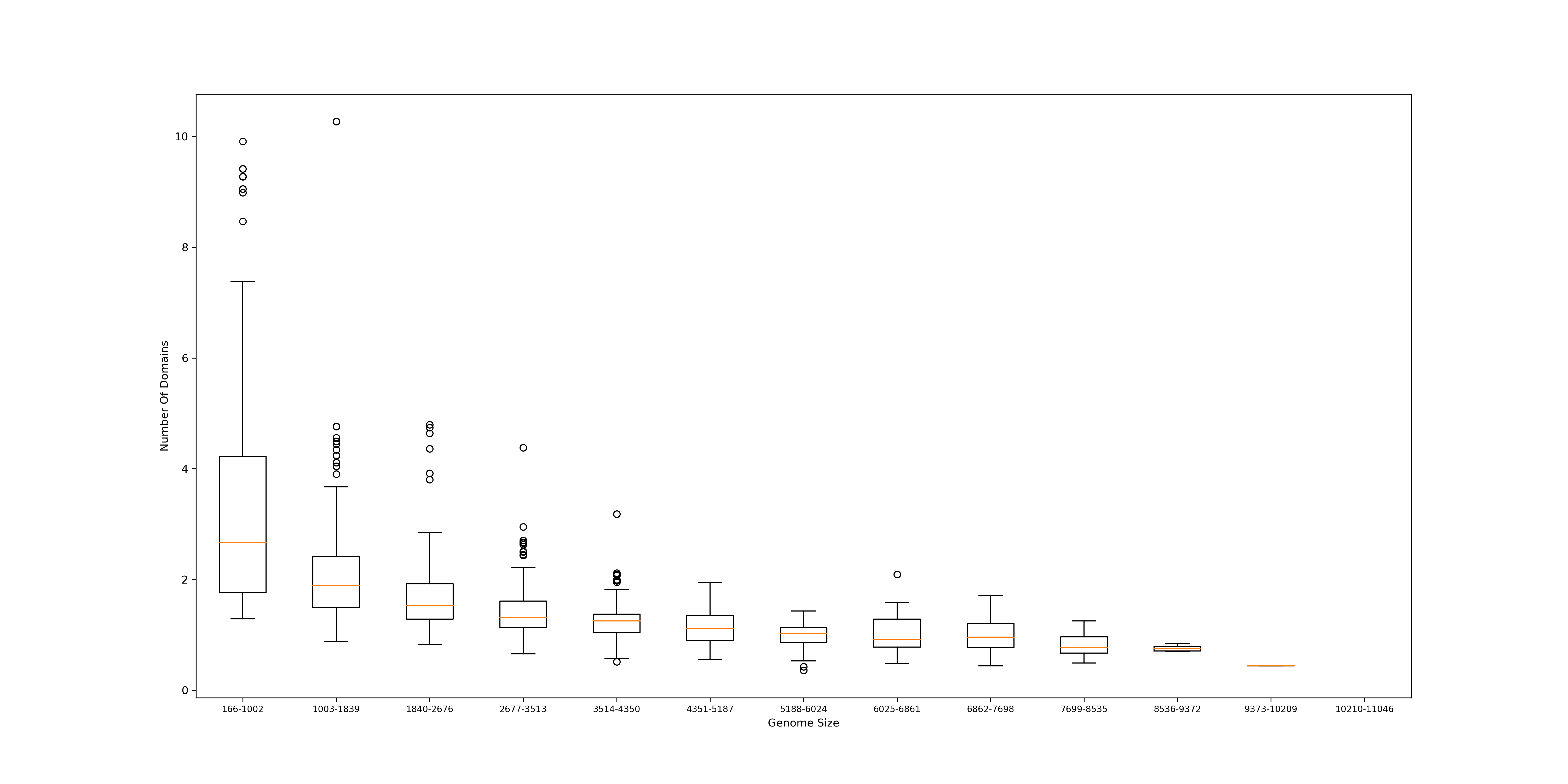

Supplement: S1 File — On the X-axis of each graph, genome size ranges are displayed in 13 windows, with a range of 836 ORFs each. On the Y-axis are the WDASs. The lines shown in the boxes are the median values. The whisker caps represent the minimum and maximum values. Superfamily IDs correspond to the names in Table 1. (ZIP) [file pone.0226604.s006.zip › Supplemnetary_material_S1/Figure_WSByIntervals_52317.png]

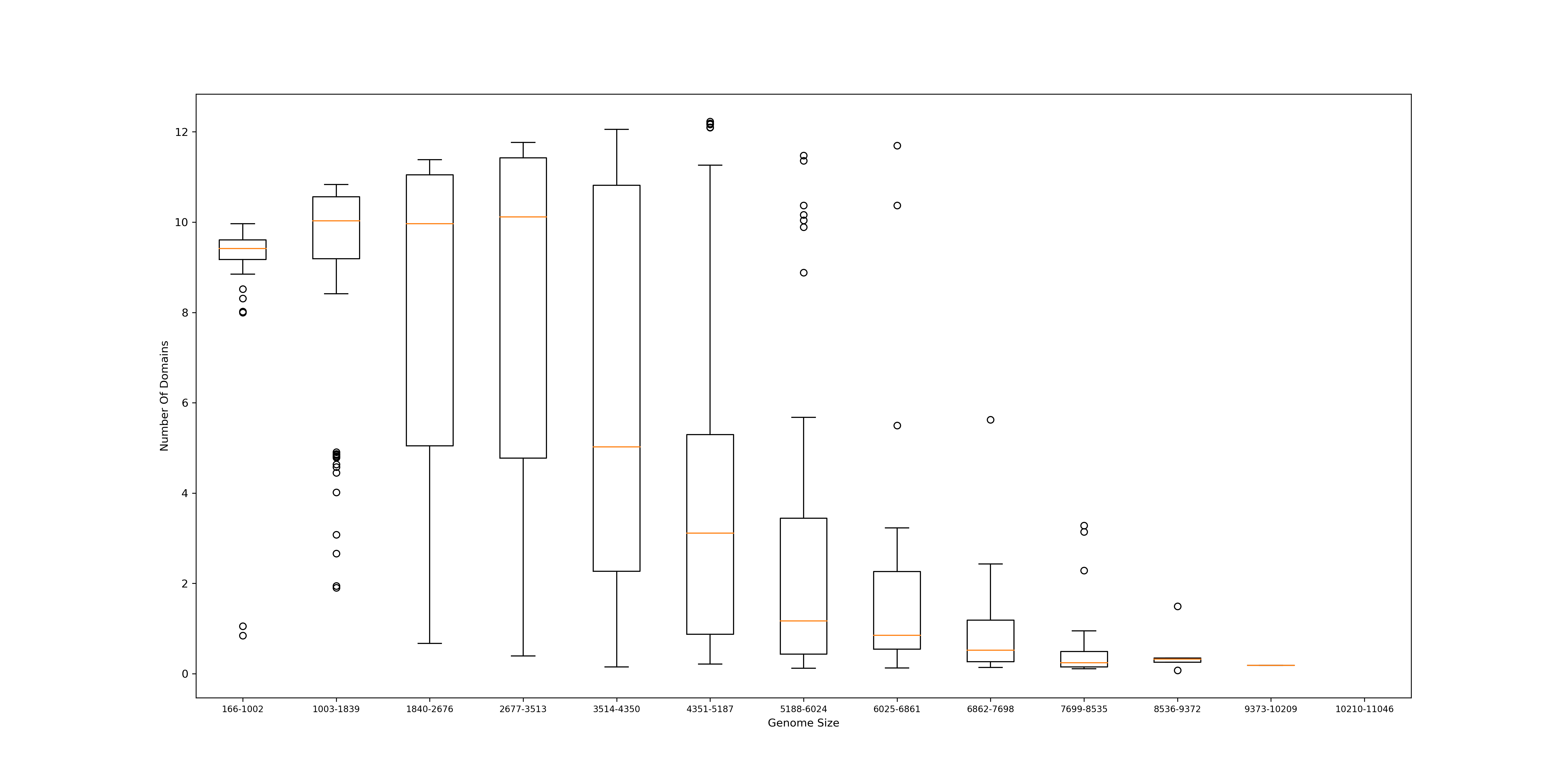

Supplement: S1 File — On the X-axis of each graph, genome size ranges are displayed in 13 windows, with a range of 836 ORFs each. On the Y-axis are the WDASs. The lines shown in the boxes are the median values. The whisker caps represent the minimum and maximum values. Superfamily IDs correspond to the names in Table 1. (ZIP) [file pone.0226604.s006.zip › Supplemnetary_material_S1/Figure_WSByIntervals_47336.png]

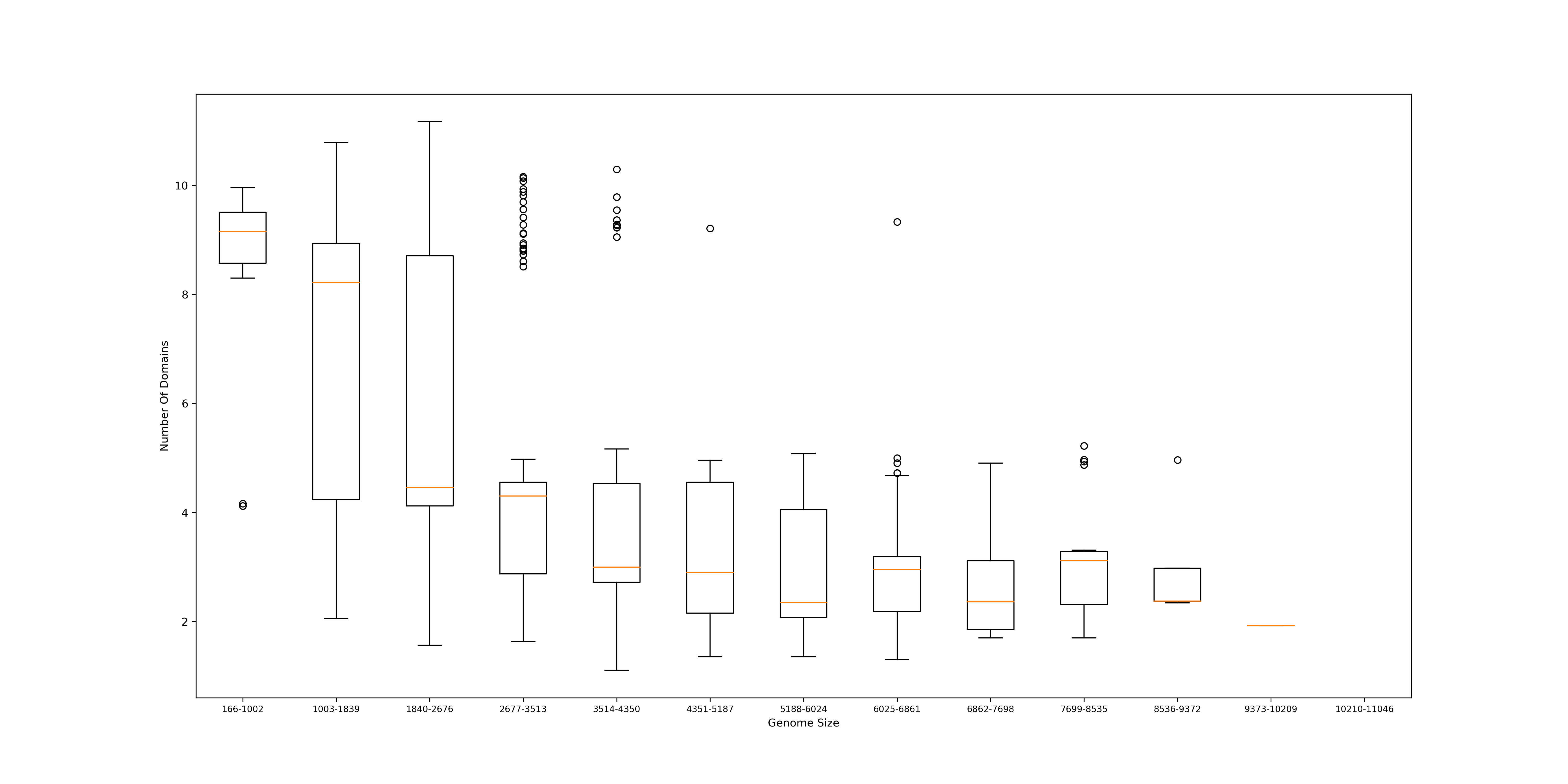

Supplement: S1 File — On the X-axis of each graph, genome size ranges are displayed in 13 windows, with a range of 836 ORFs each. On the Y-axis are the WDASs. The lines shown in the boxes are the median values. The whisker caps represent the minimum and maximum values. Superfamily IDs correspond to the names in Table 1. (ZIP) [file pone.0226604.s006.zip › Supplemnetary_material_S1/Figure_WSByIntervals_51395.png]

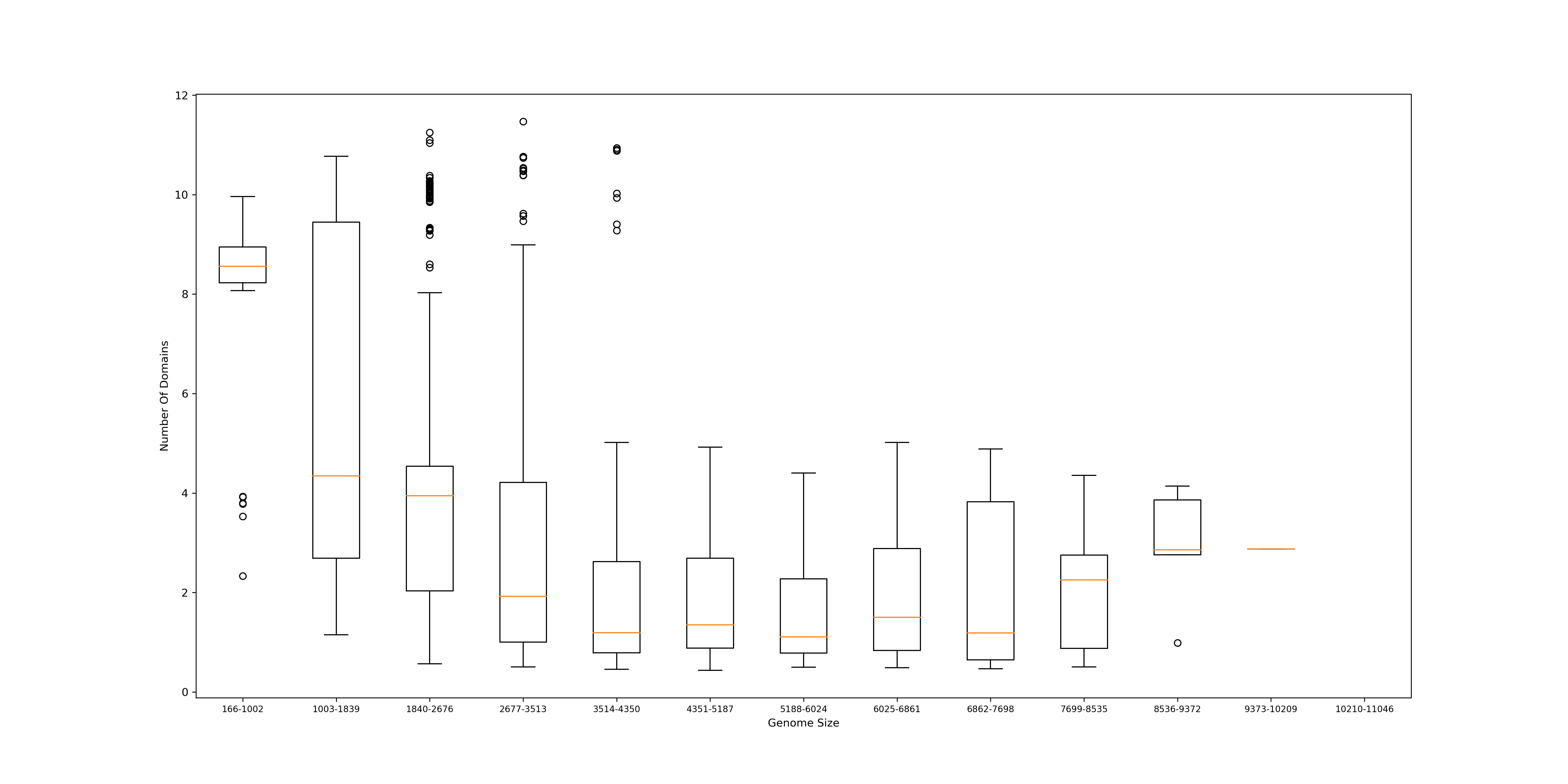

Supplement: S1 File — On the X-axis of each graph, genome size ranges are displayed in 13 windows, with a range of 836 ORFs each. On the Y-axis are the WDASs. The lines shown in the boxes are the median values. The whisker caps represent the minimum and maximum values. Superfamily IDs correspond to the names in Table 1. (ZIP) [file pone.0226604.s006.zip › Supplemnetary_material_S1/Figure_WSByIntervals_82866.png]

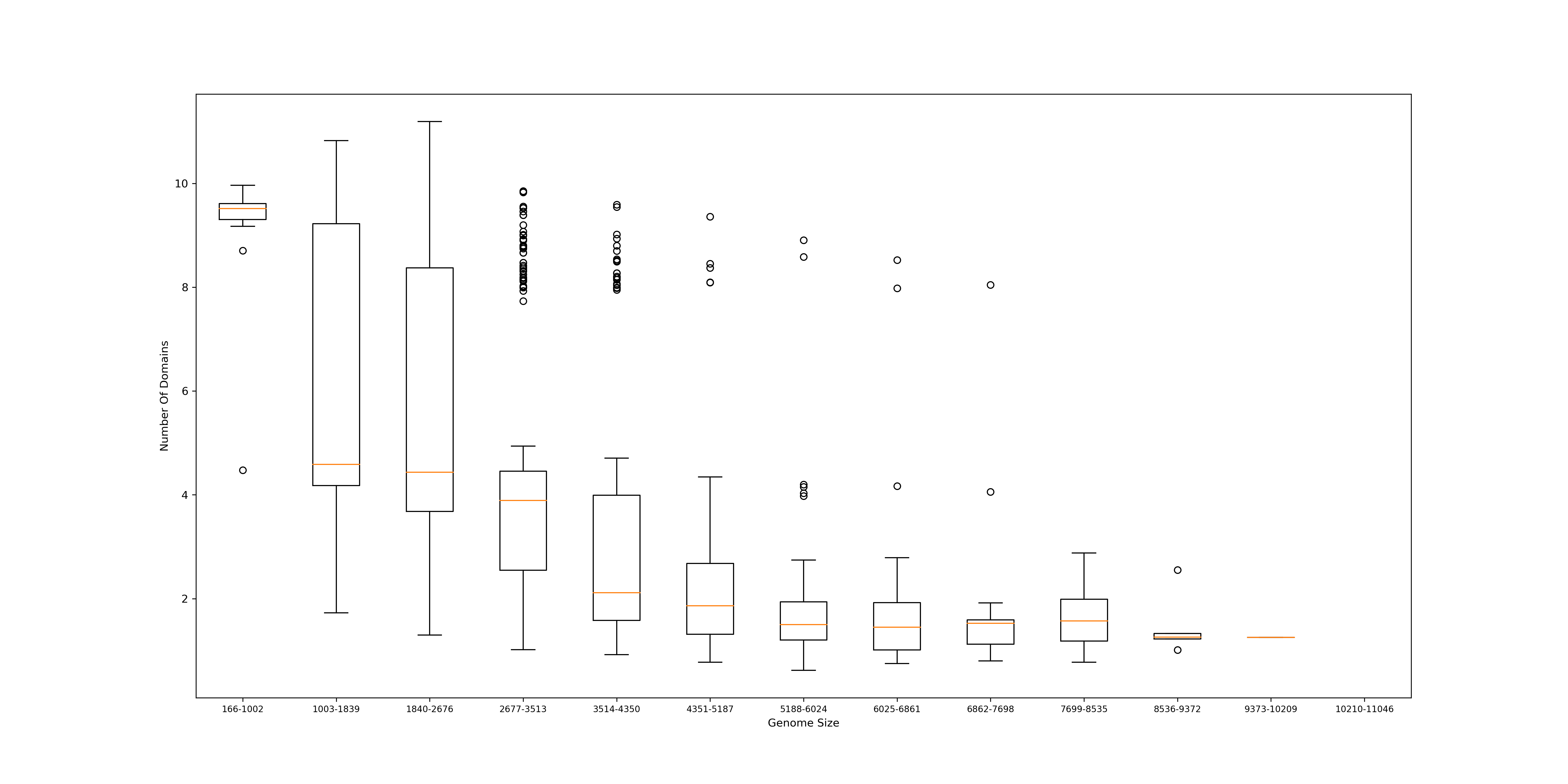

Supplement: S1 File — On the X-axis of each graph, genome size ranges are displayed in 13 windows, with a range of 836 ORFs each. On the Y-axis are the WDASs. The lines shown in the boxes are the median values. The whisker caps represent the minimum and maximum values. Superfamily IDs correspond to the names in Table 1. (ZIP) [file pone.0226604.s006.zip › Supplemnetary_material_S1/Figure_WSByIntervals_51182.png]

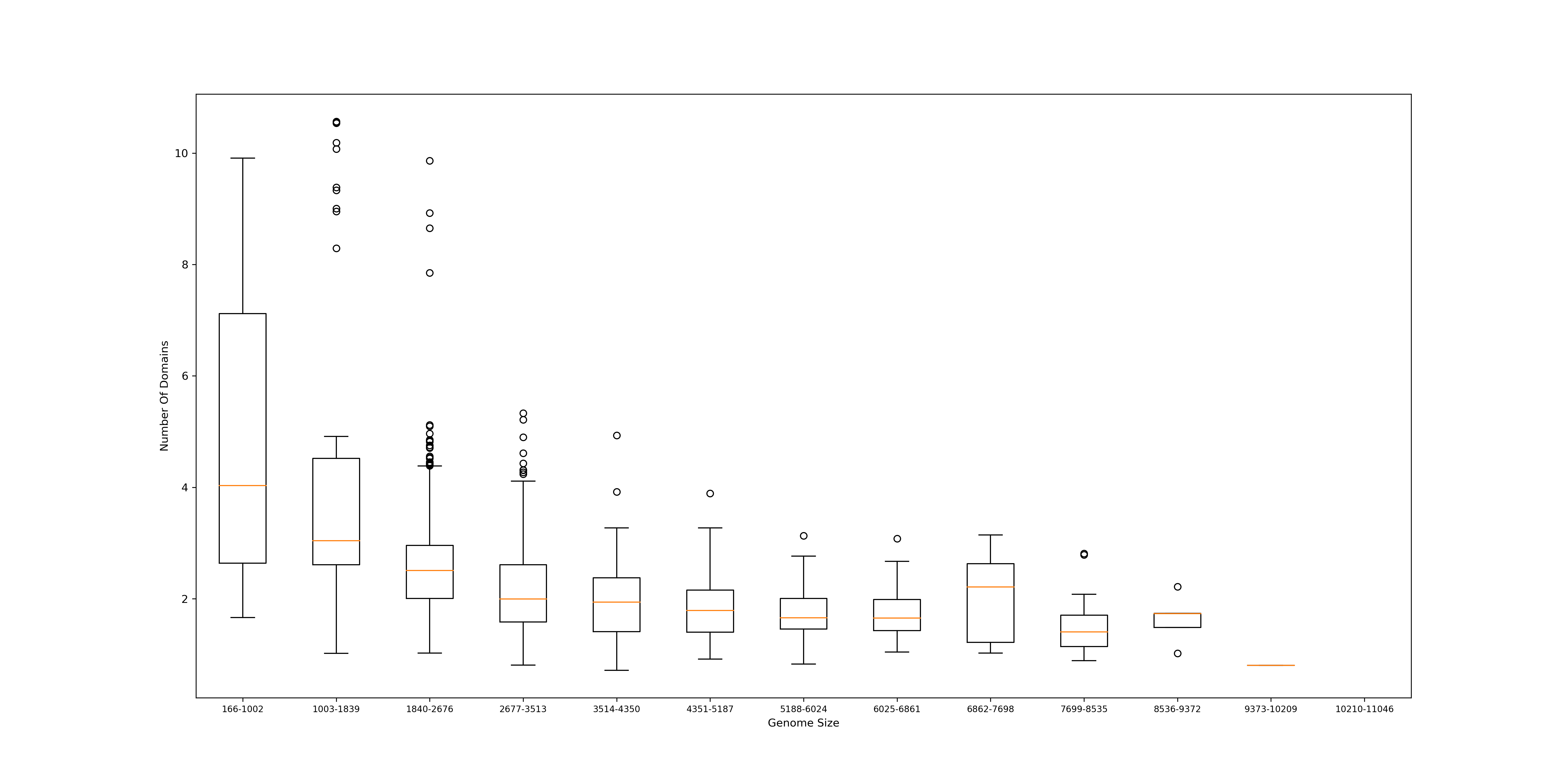

Supplement: S1 File — On the X-axis of each graph, genome size ranges are displayed in 13 windows, with a range of 836 ORFs each. On the Y-axis are the WDASs. The lines shown in the boxes are the median values. The whisker caps represent the minimum and maximum values. Superfamily IDs correspond to the names in Table 1. (ZIP) [file pone.0226604.s006.zip › Supplemnetary_material_S1/Figure_WSByIntervals_53187.png]

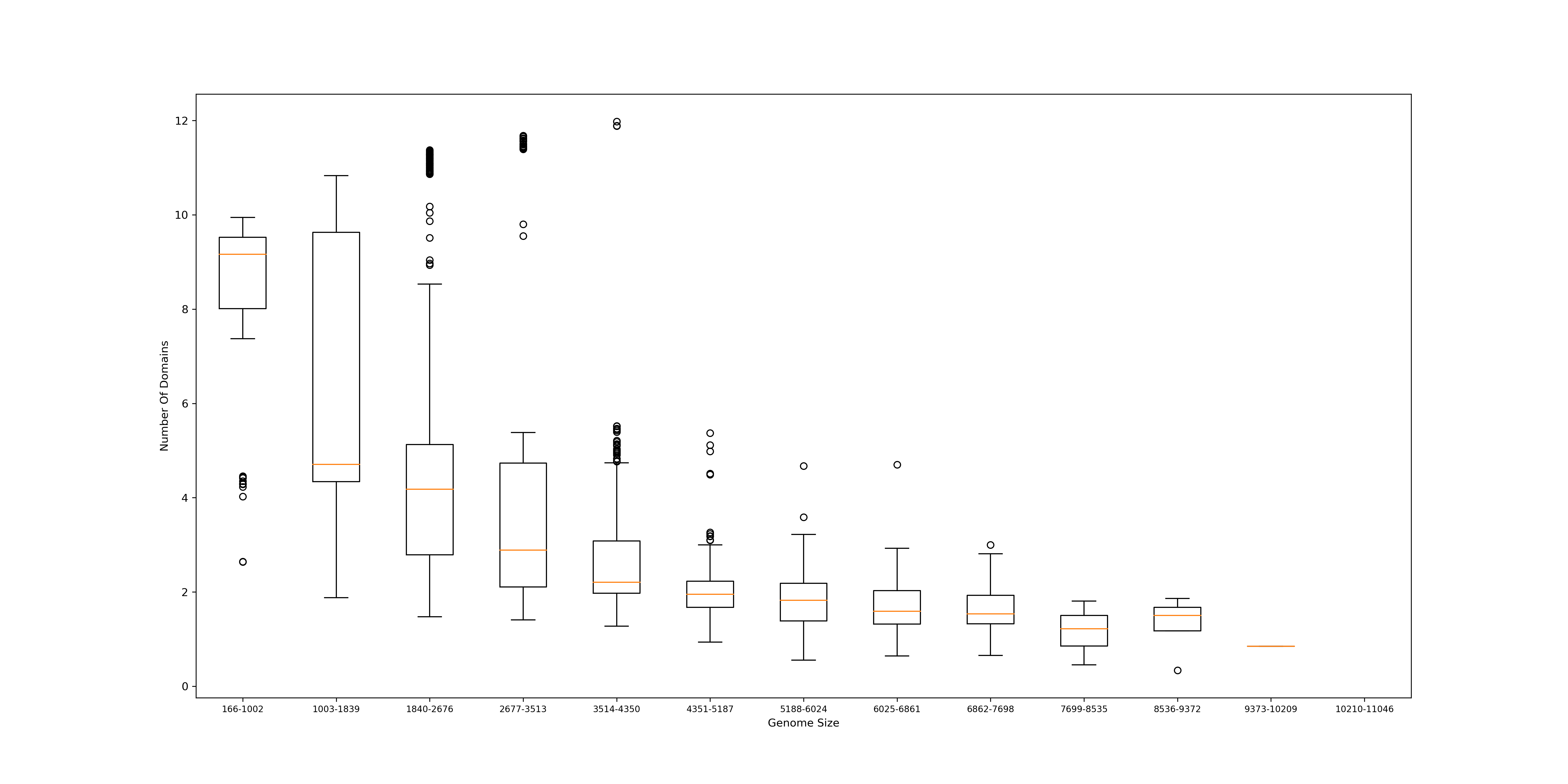

Supplement: S1 File — On the X-axis of each graph, genome size ranges are displayed in 13 windows, with a range of 836 ORFs each. On the Y-axis are the WDASs. The lines shown in the boxes are the median values. The whisker caps represent the minimum and maximum values. Superfamily IDs correspond to the names in Table 1. (ZIP) [file pone.0226604.s006.zip › Supplemnetary_material_S1/Figure_WSByIntervals_50129.png]

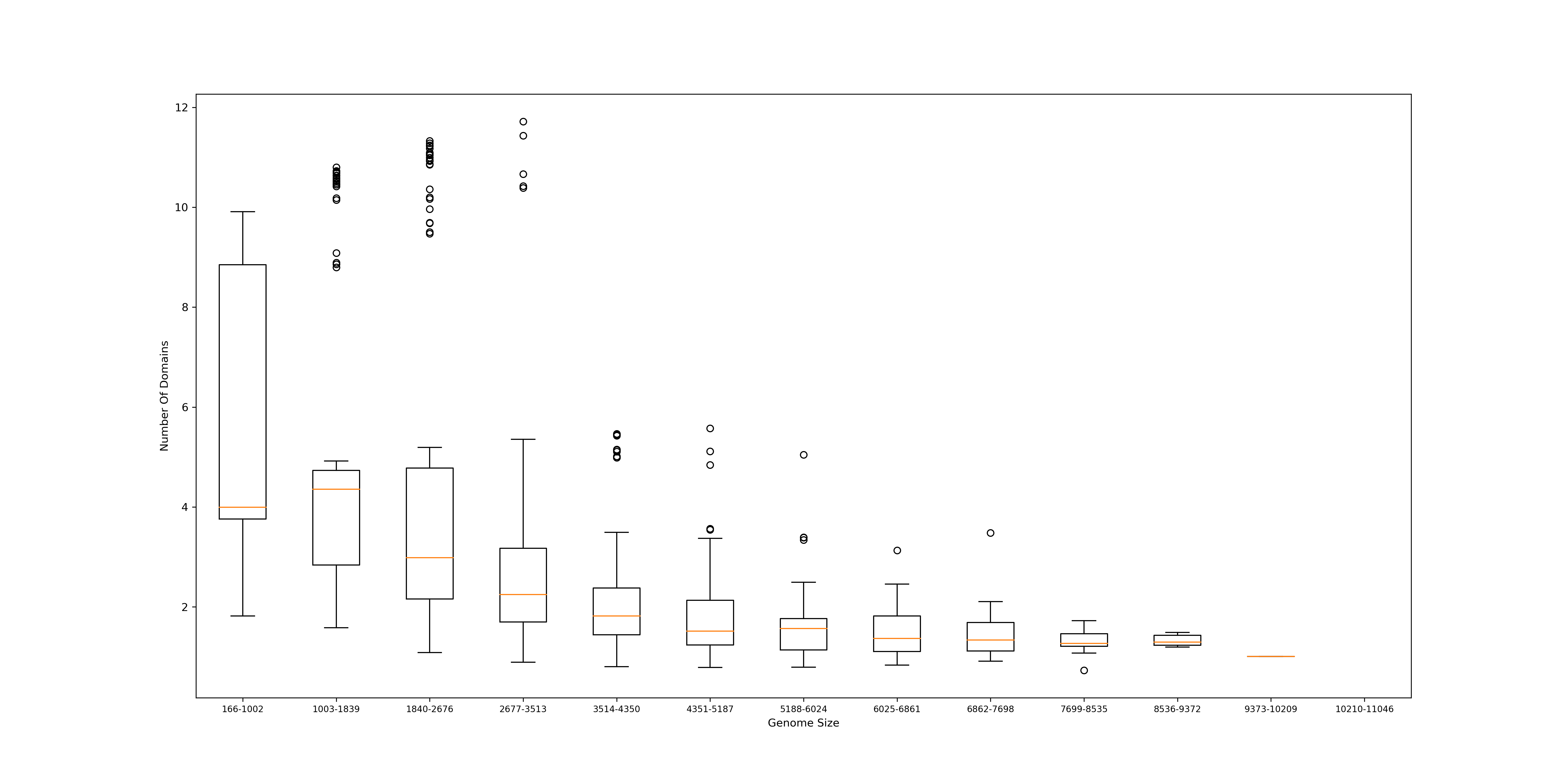

Supplement: S1 File — On the X-axis of each graph, genome size ranges are displayed in 13 windows, with a range of 836 ORFs each. On the Y-axis are the WDASs. The lines shown in the boxes are the median values. The whisker caps represent the minimum and maximum values. Superfamily IDs correspond to the names in Table 1. (ZIP) [file pone.0226604.s006.zip › Supplemnetary_material_S1/Figure_WSByIntervals_56176.png]

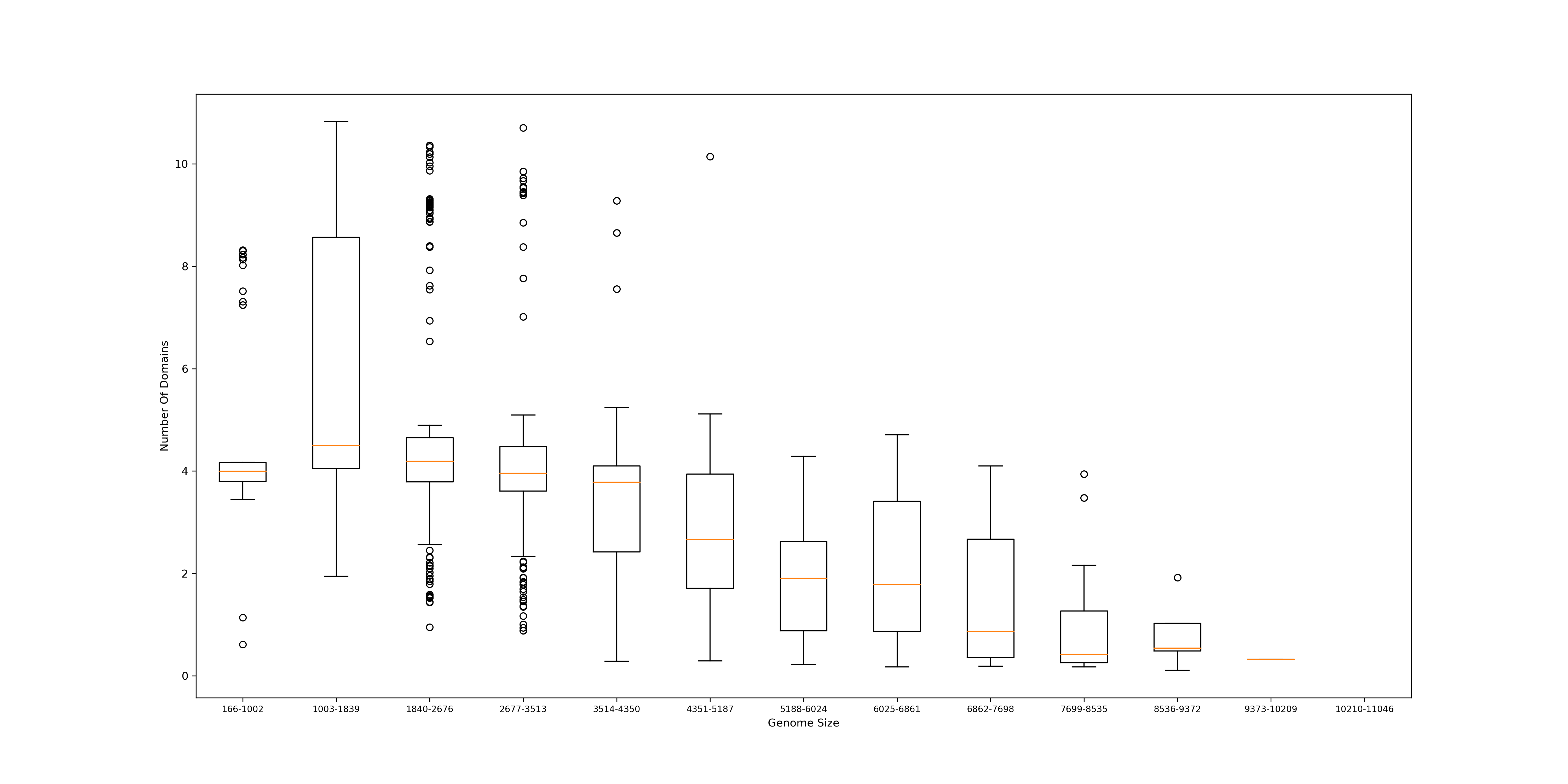

Supplement: S1 File — On the X-axis of each graph, genome size ranges are displayed in 13 windows, with a range of 836 ORFs each. On the Y-axis are the WDASs. The lines shown in the boxes are the median values. The whisker caps represent the minimum and maximum values. Superfamily IDs correspond to the names in Table 1. (ZIP) [file pone.0226604.s006.zip › Supplemnetary_material_S1/Figure_WSByIntervals_53901.png]

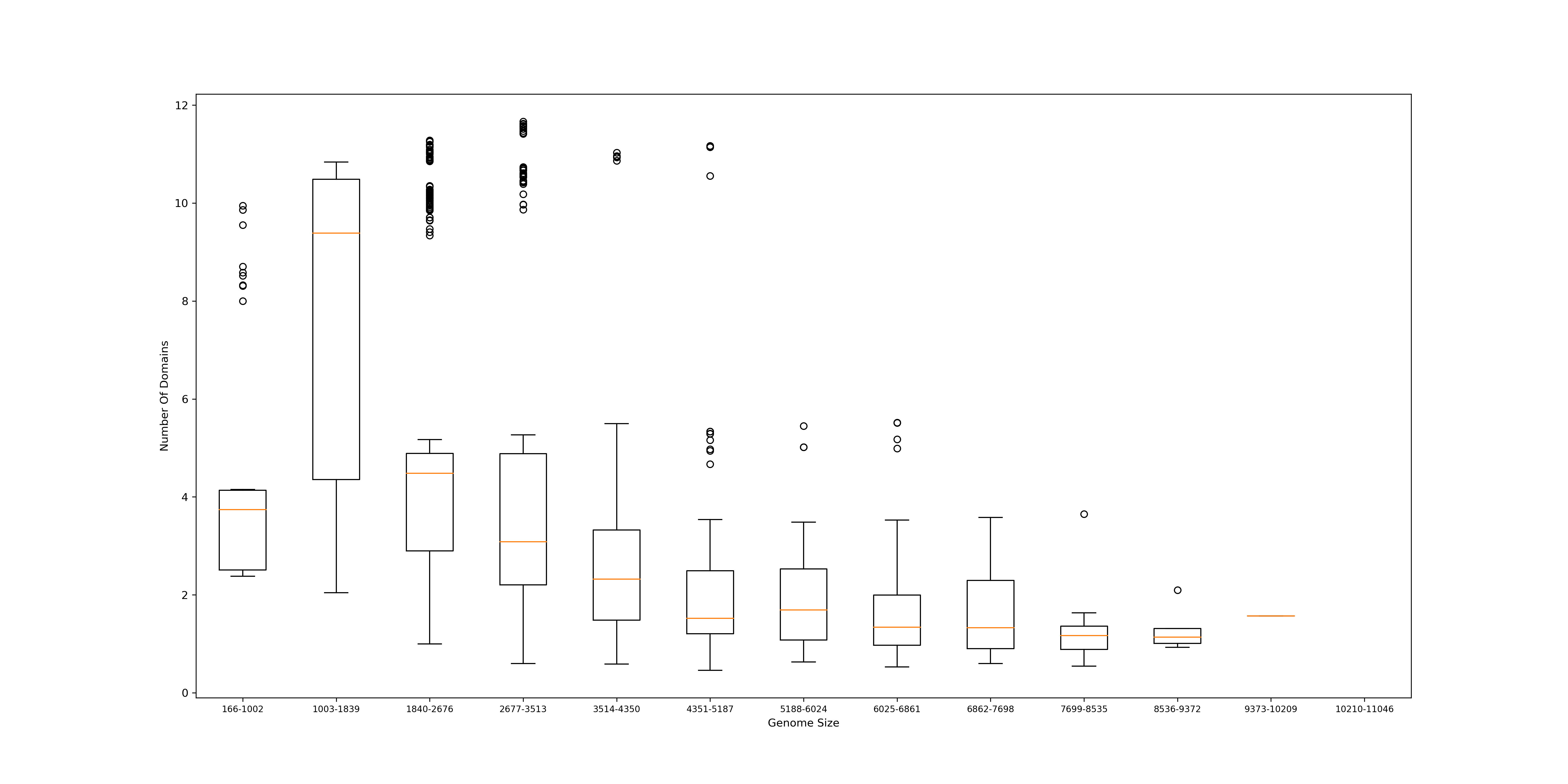

Supplement: S1 File — On the X-axis of each graph, genome size ranges are displayed in 13 windows, with a range of 836 ORFs each. On the Y-axis are the WDASs. The lines shown in the boxes are the median values. The whisker caps represent the minimum and maximum values. Superfamily IDs correspond to the names in Table 1. (ZIP) [file pone.0226604.s006.zip › Supplemnetary_material_S1/Figure_WSByIntervals_63380.png]

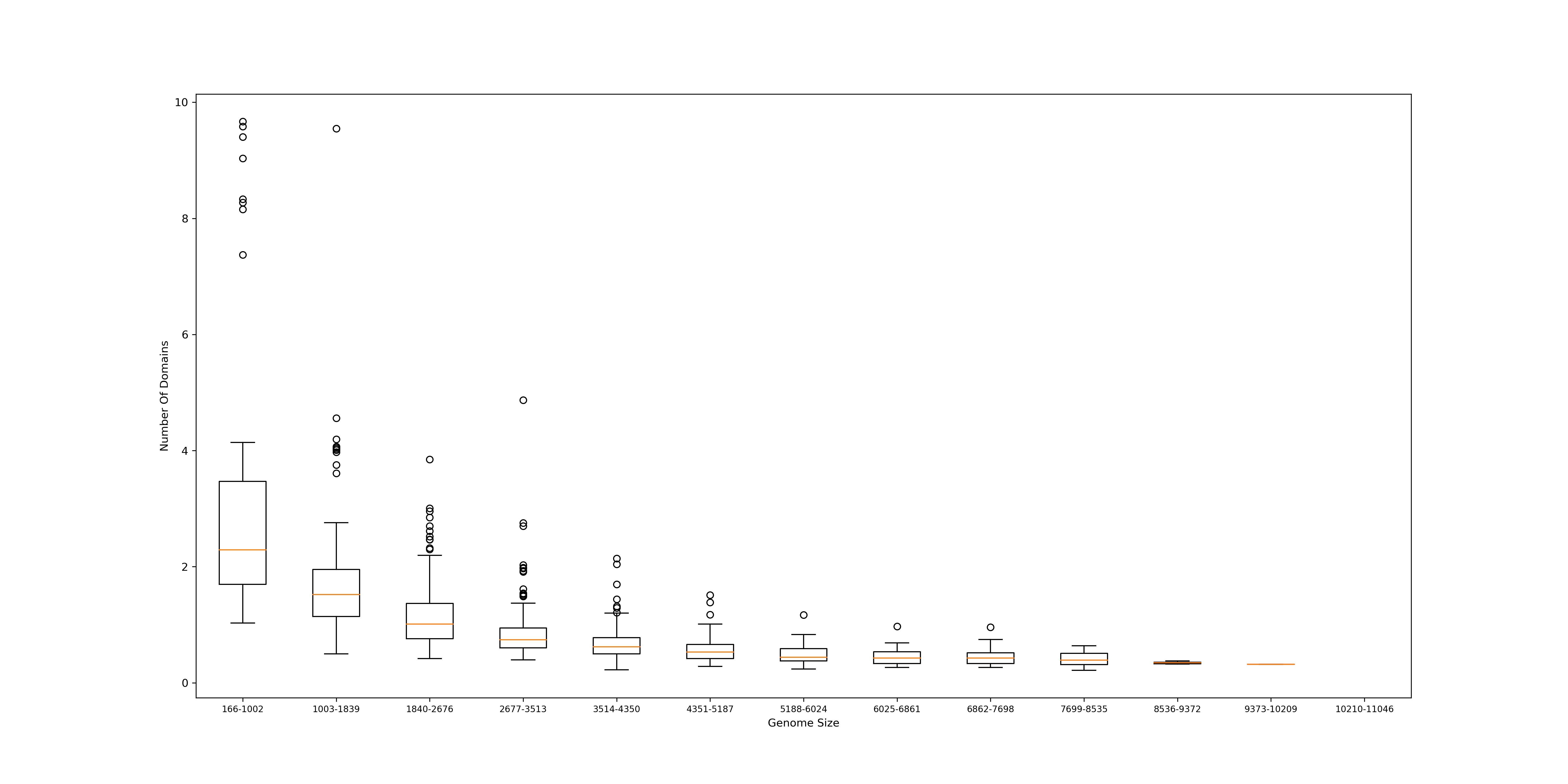

Supplement: S1 File — On the X-axis of each graph, genome size ranges are displayed in 13 windows, with a range of 836 ORFs each. On the Y-axis are the WDASs. The lines shown in the boxes are the median values. The whisker caps represent the minimum and maximum values. Superfamily IDs correspond to the names in Table 1. (ZIP) [file pone.0226604.s006.zip › Supplemnetary_material_S1/Figure_WSByIntervals_51905.png]

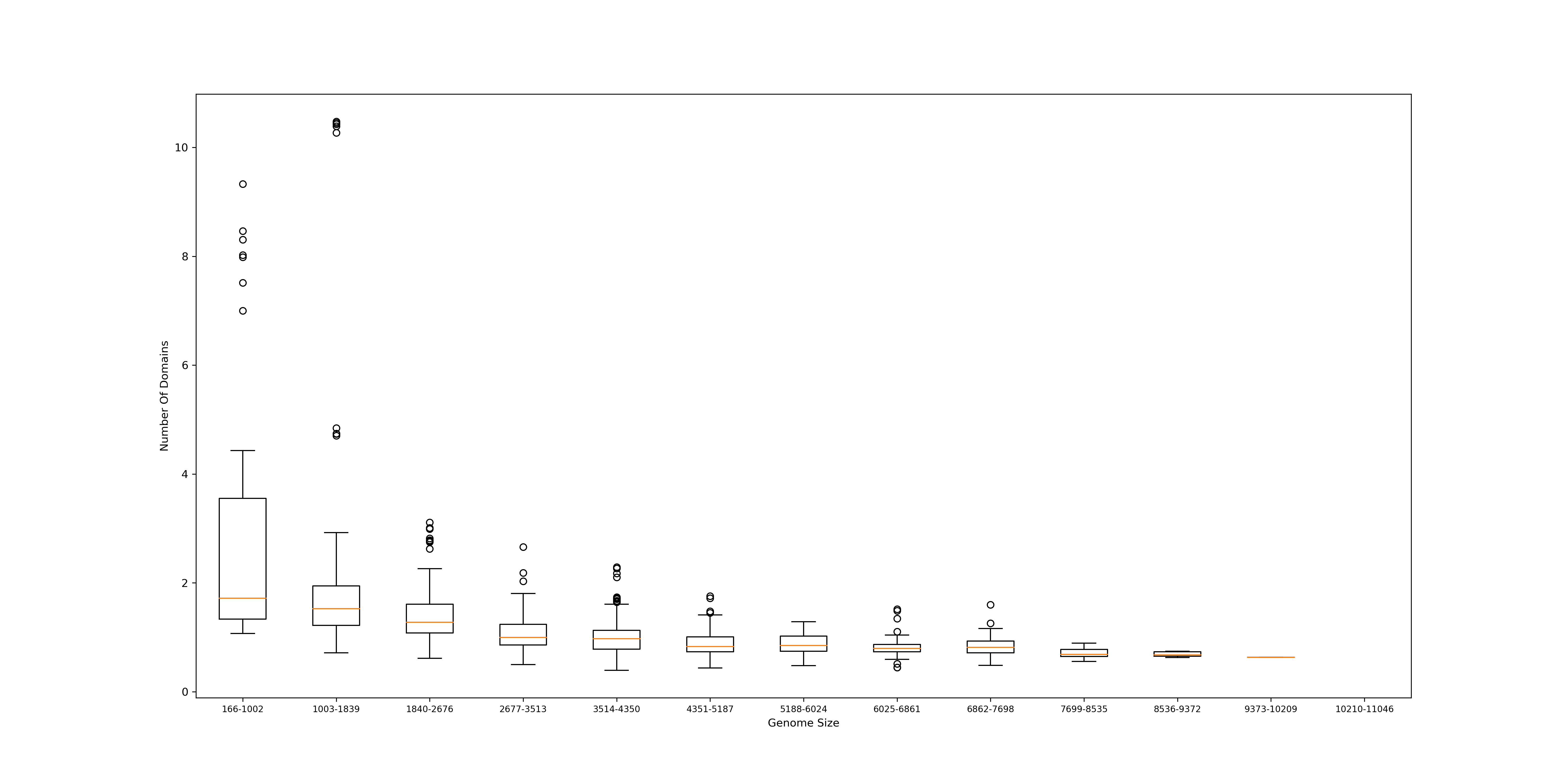

Supplement: S1 File — On the X-axis of each graph, genome size ranges are displayed in 13 windows, with a range of 836 ORFs each. On the Y-axis are the WDASs. The lines shown in the boxes are the median values. The whisker caps represent the minimum and maximum values. Superfamily IDs correspond to the names in Table 1. (ZIP) [file pone.0226604.s006.zip › Supplemnetary_material_S1/Figure_WSByIntervals_56059.png]

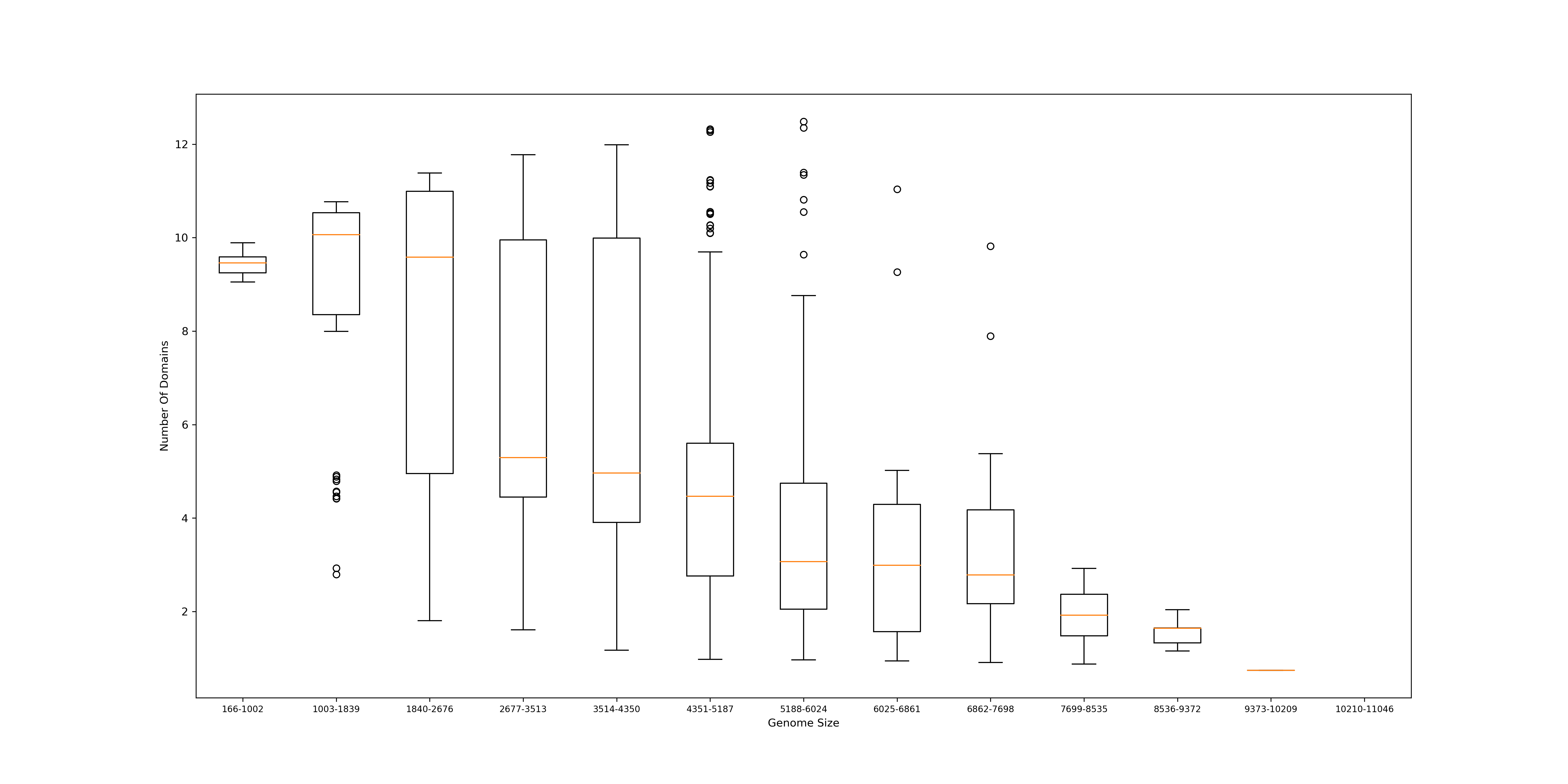

Supplement: S1 File — On the X-axis of each graph, genome size ranges are displayed in 13 windows, with a range of 836 ORFs each. On the Y-axis are the WDASs. The lines shown in the boxes are the median values. The whisker caps represent the minimum and maximum values. Superfamily IDs correspond to the names in Table 1. (ZIP) [file pone.0226604.s006.zip › Supplemnetary_material_S1/Figure_WSByIntervals_55961.png]

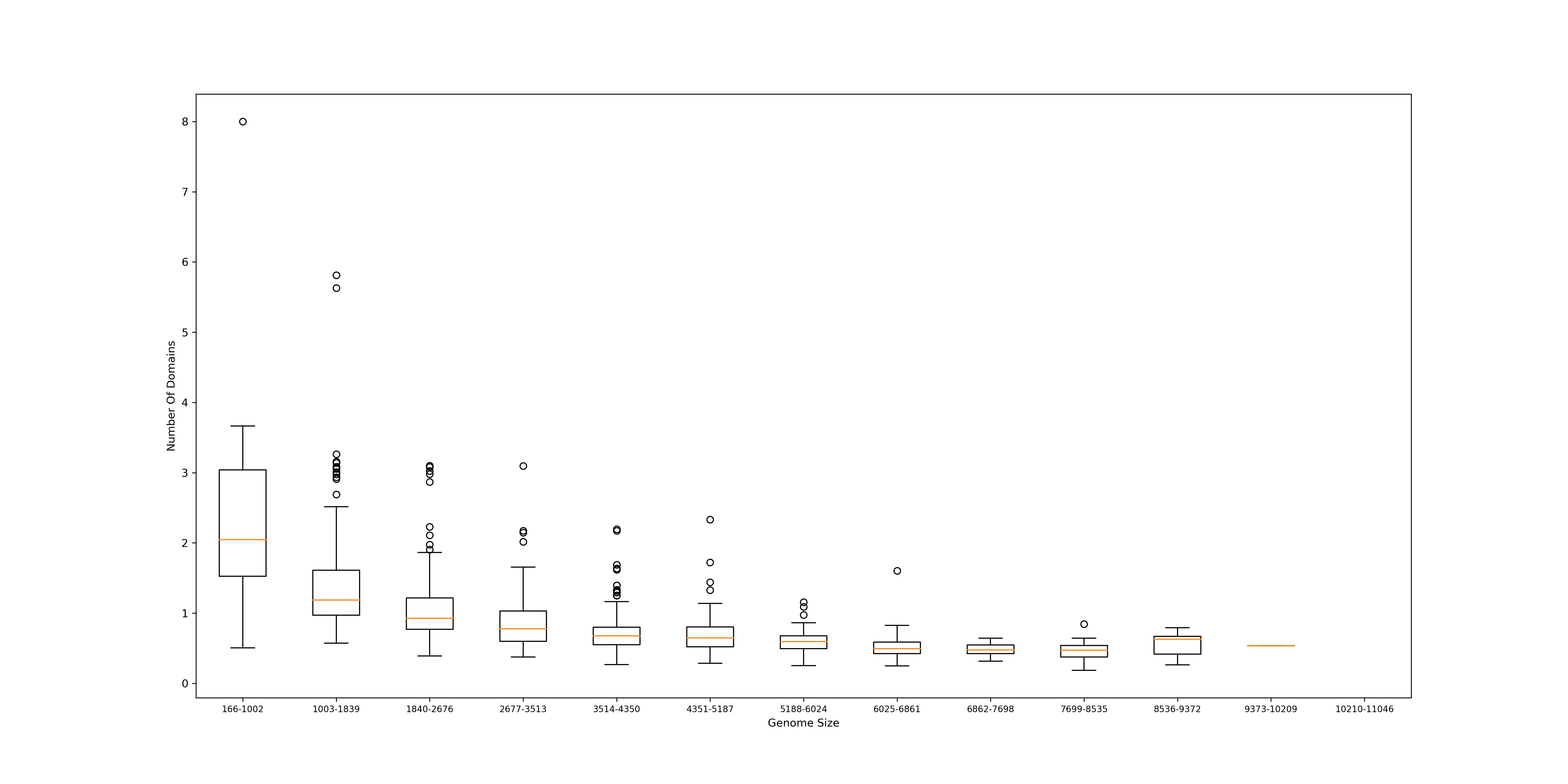

Supplement: S1 File — On the X-axis of each graph, genome size ranges are displayed in 13 windows, with a range of 836 ORFs each. On the Y-axis are the WDASs. The lines shown in the boxes are the median values. The whisker caps represent the minimum and maximum values. Superfamily IDs correspond to the names in Table 1. (ZIP) [file pone.0226604.s006.zip › Supplemnetary_material_S1/Figure_WSByIntervals_53335.png]

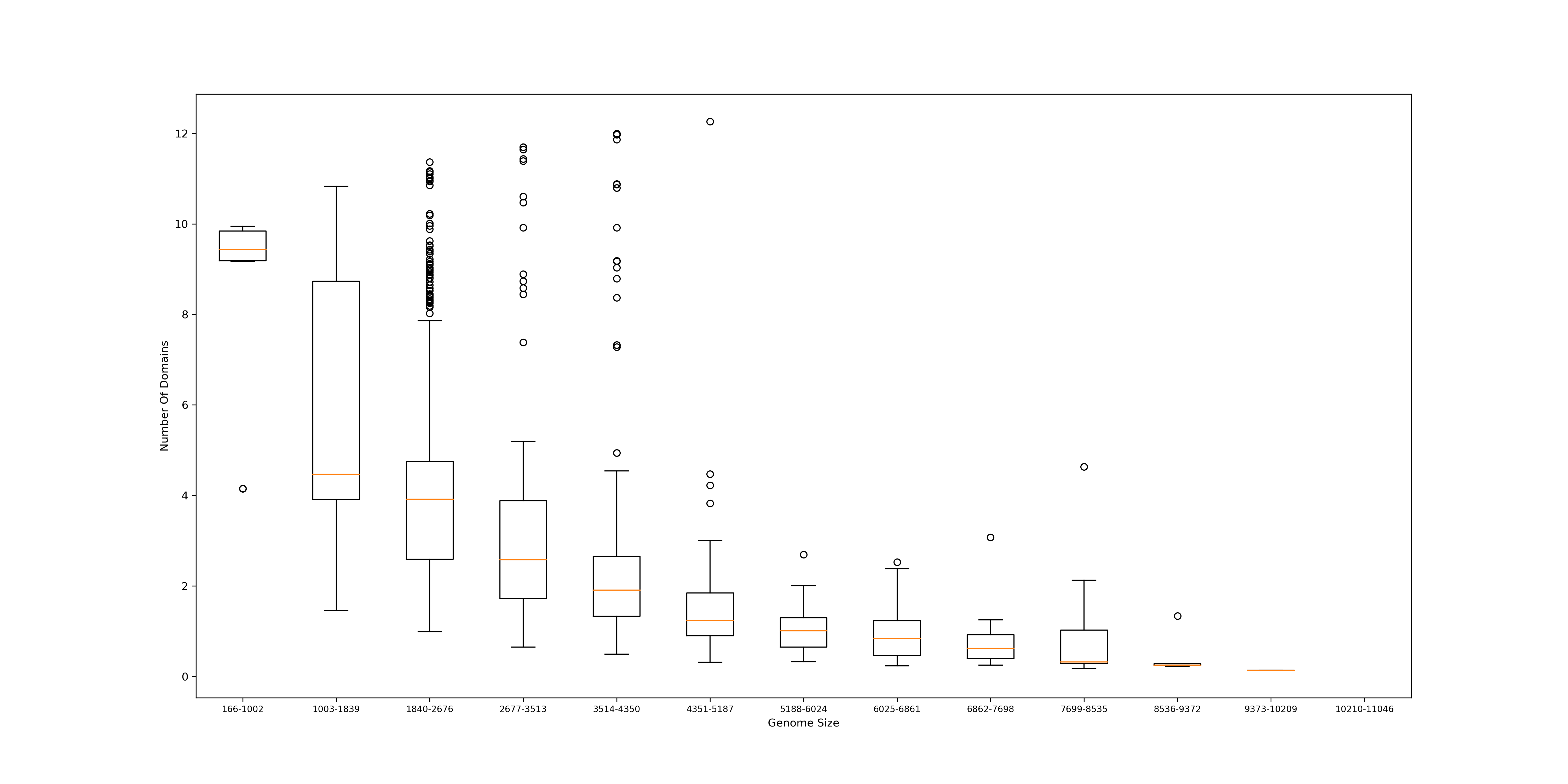

Supplement: S1 File — On the X-axis of each graph, genome size ranges are displayed in 13 windows, with a range of 836 ORFs each. On the Y-axis are the WDASs. The lines shown in the boxes are the median values. The whisker caps represent the minimum and maximum values. Superfamily IDs correspond to the names in Table 1. (ZIP) [file pone.0226604.s006.zip › Supplemnetary_material_S1/Figure_WSByIntervals_46894.png]

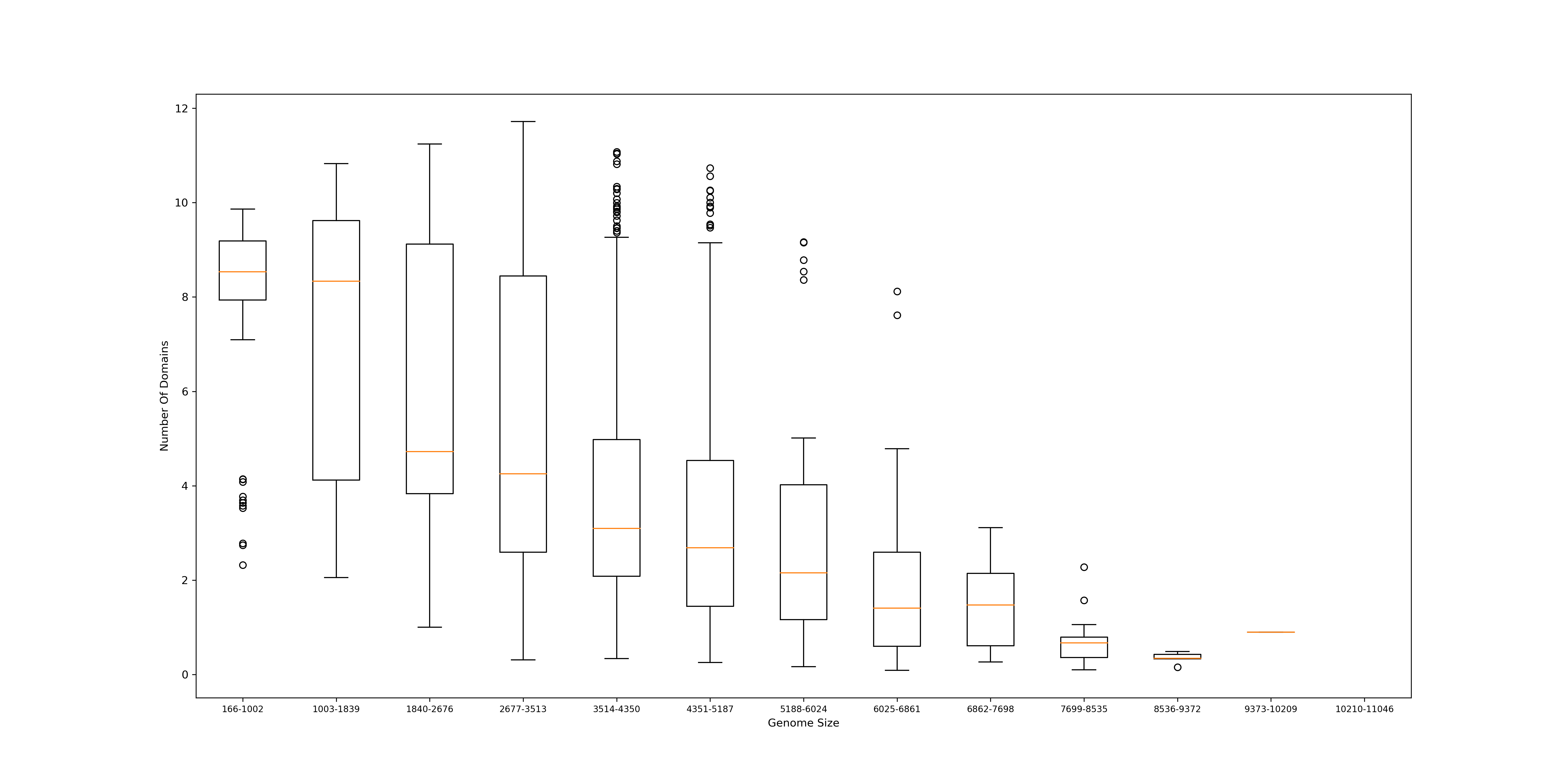

Supplement: S1 File — On the X-axis of each graph, genome size ranges are displayed in 13 windows, with a range of 836 ORFs each. On the Y-axis are the WDASs. The lines shown in the boxes are the median values. The whisker caps represent the minimum and maximum values. Superfamily IDs correspond to the names in Table 1. (ZIP) [file pone.0226604.s006.zip › Supplemnetary_material_S1/Figure_WSByIntervals_56112.png]

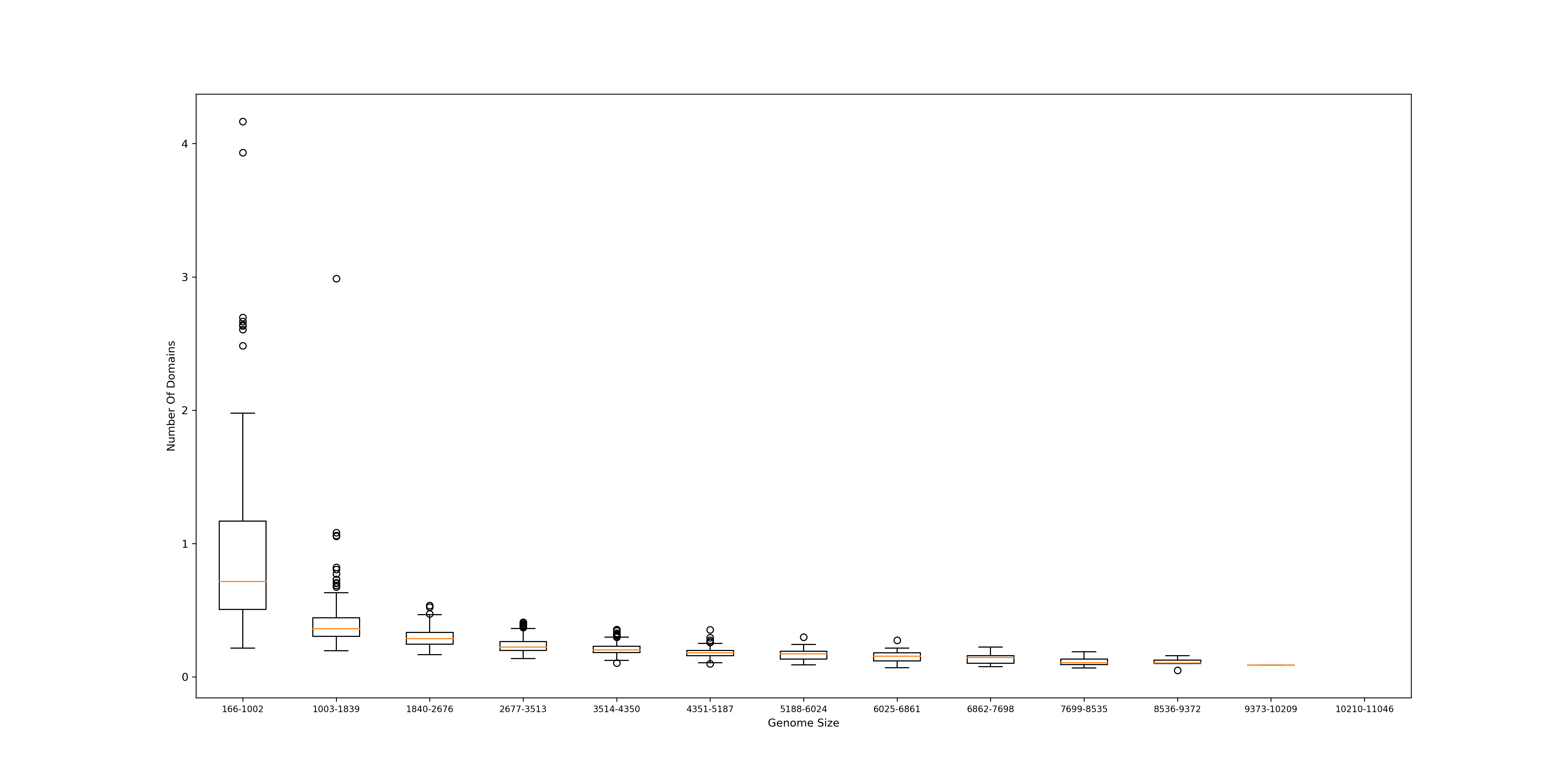

Supplement: S1 File — On the X-axis of each graph, genome size ranges are displayed in 13 windows, with a range of 836 ORFs each. On the Y-axis are the WDASs. The lines shown in the boxes are the median values. The whisker caps represent the minimum and maximum values. Superfamily IDs correspond to the names in Table 1. (ZIP) [file pone.0226604.s006.zip › Supplemnetary_material_S1/Figure_WSByIntervals_51735.png]

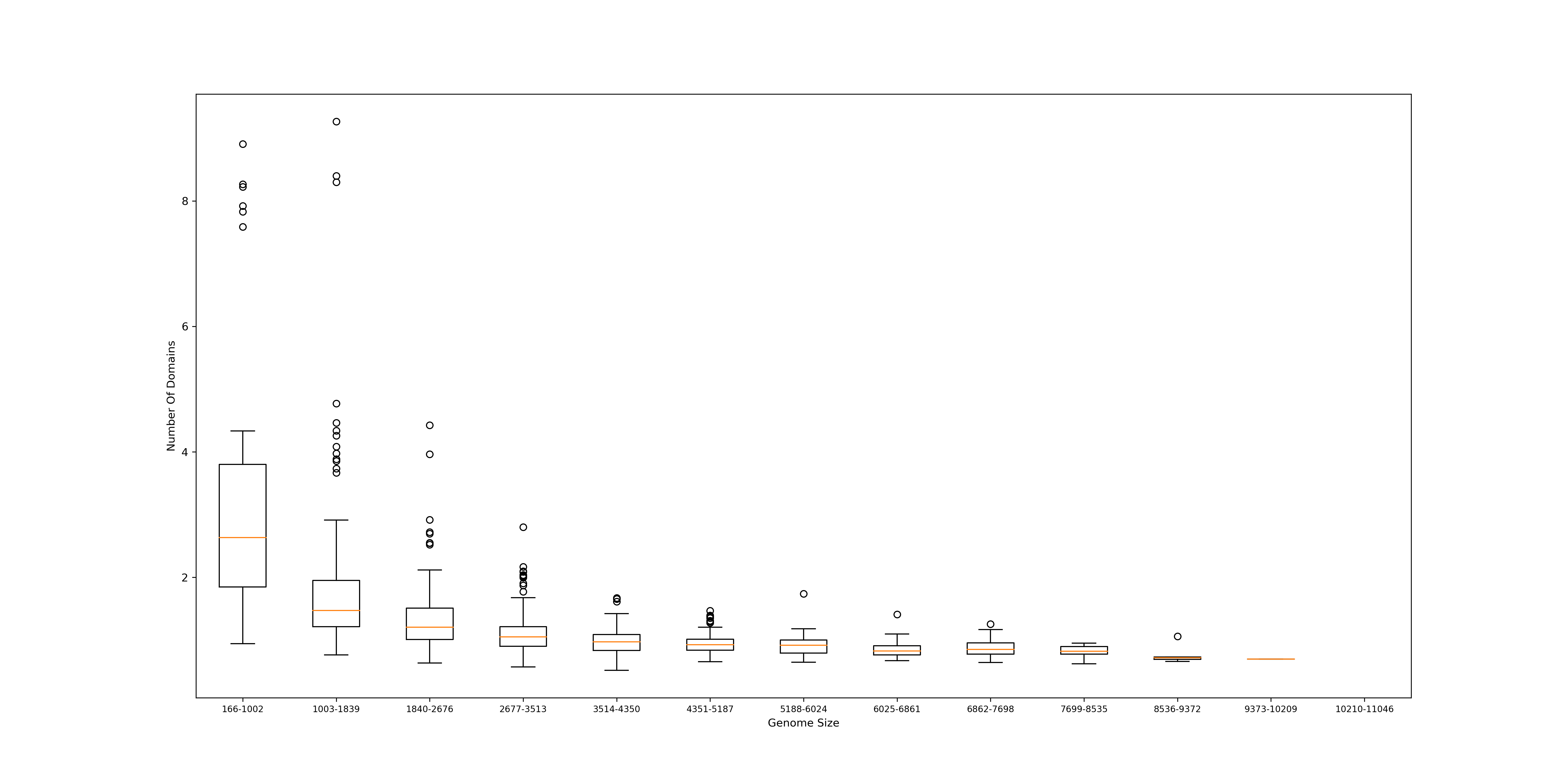

Supplement: S1 File — On the X-axis of each graph, genome size ranges are displayed in 13 windows, with a range of 836 ORFs each. On the Y-axis are the WDASs. The lines shown in the boxes are the median values. The whisker caps represent the minimum and maximum values. Superfamily IDs correspond to the names in Table 1. (ZIP) [file pone.0226604.s006.zip › Supplemnetary_material_S1/Figure_WSByIntervals_52402.png]

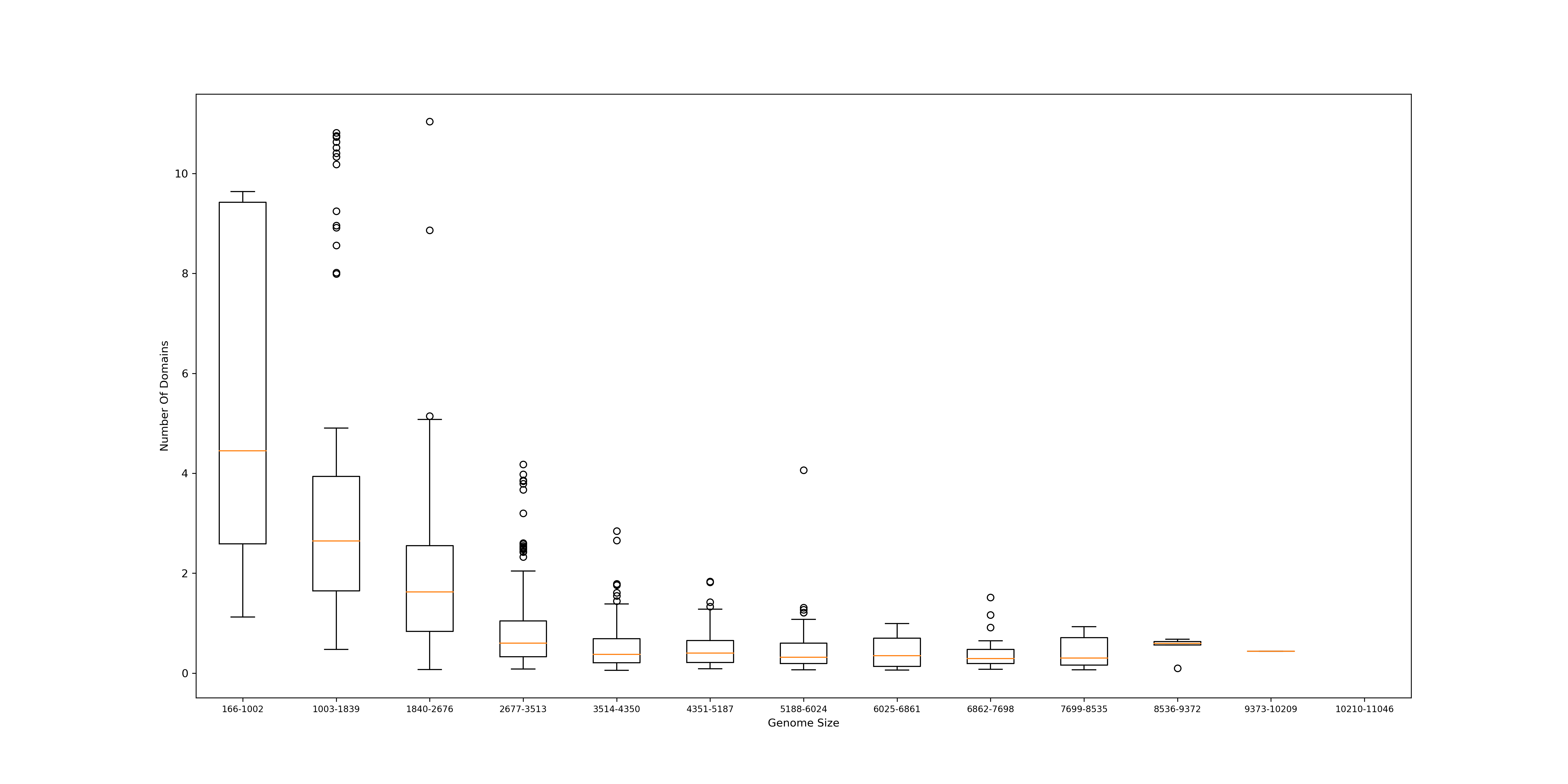

Supplement: S1 File — On the X-axis of each graph, genome size ranges are displayed in 13 windows, with a range of 836 ORFs each. On the Y-axis are the WDASs. The lines shown in the boxes are the median values. The whisker caps represent the minimum and maximum values. Superfamily IDs correspond to the names in Table 1. (ZIP) [file pone.0226604.s006.zip › Supplemnetary_material_S1/Figure_WSByIntervals_52172.png]

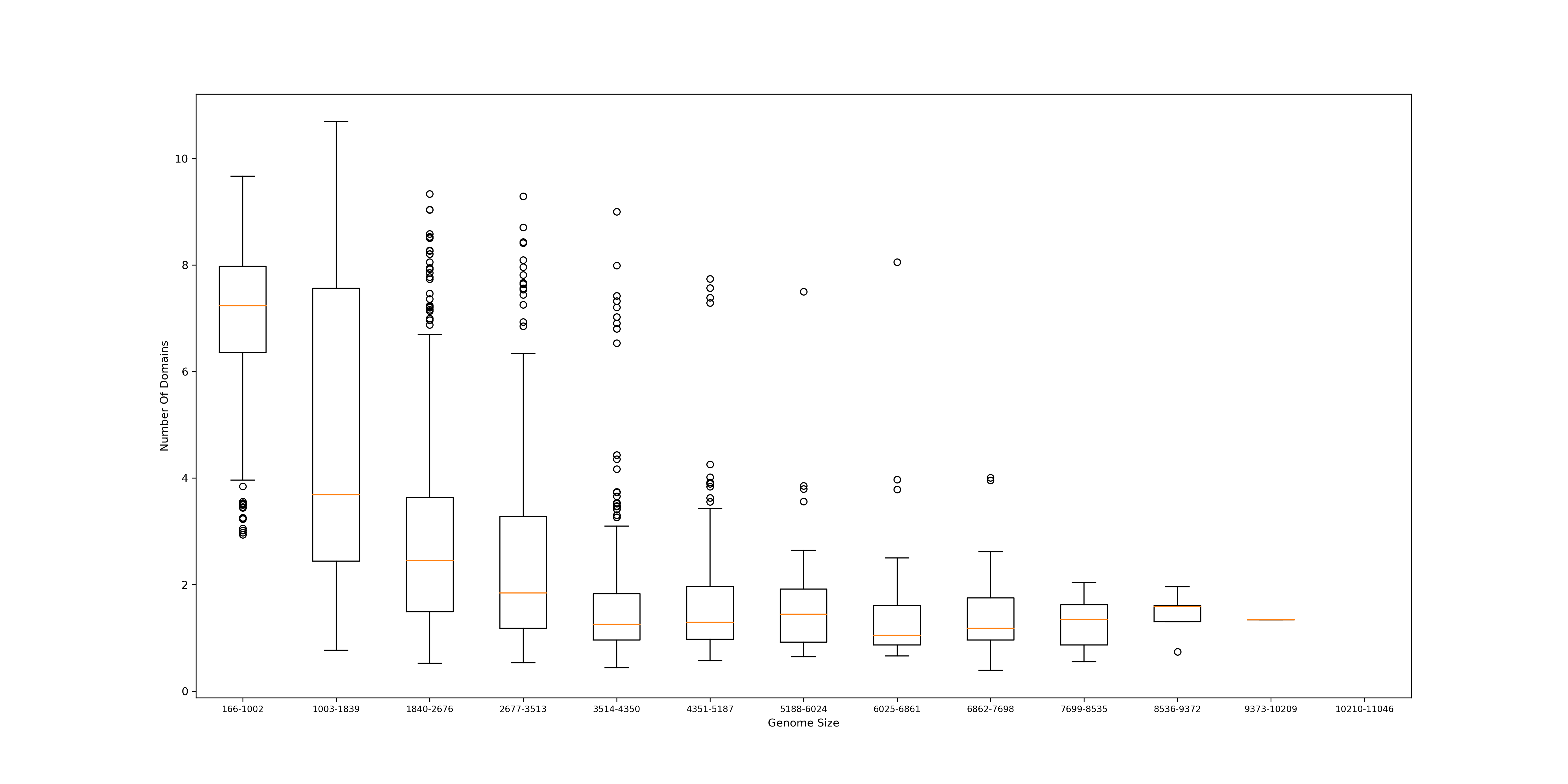

Supplement: S1 File — On the X-axis of each graph, genome size ranges are displayed in 13 windows, with a range of 836 ORFs each. On the Y-axis are the WDASs. The lines shown in the boxes are the median values. The whisker caps represent the minimum and maximum values. Superfamily IDs correspond to the names in Table 1. (ZIP) [file pone.0226604.s006.zip › Supplemnetary_material_S1/Figure_WSByIntervals_52833.png]

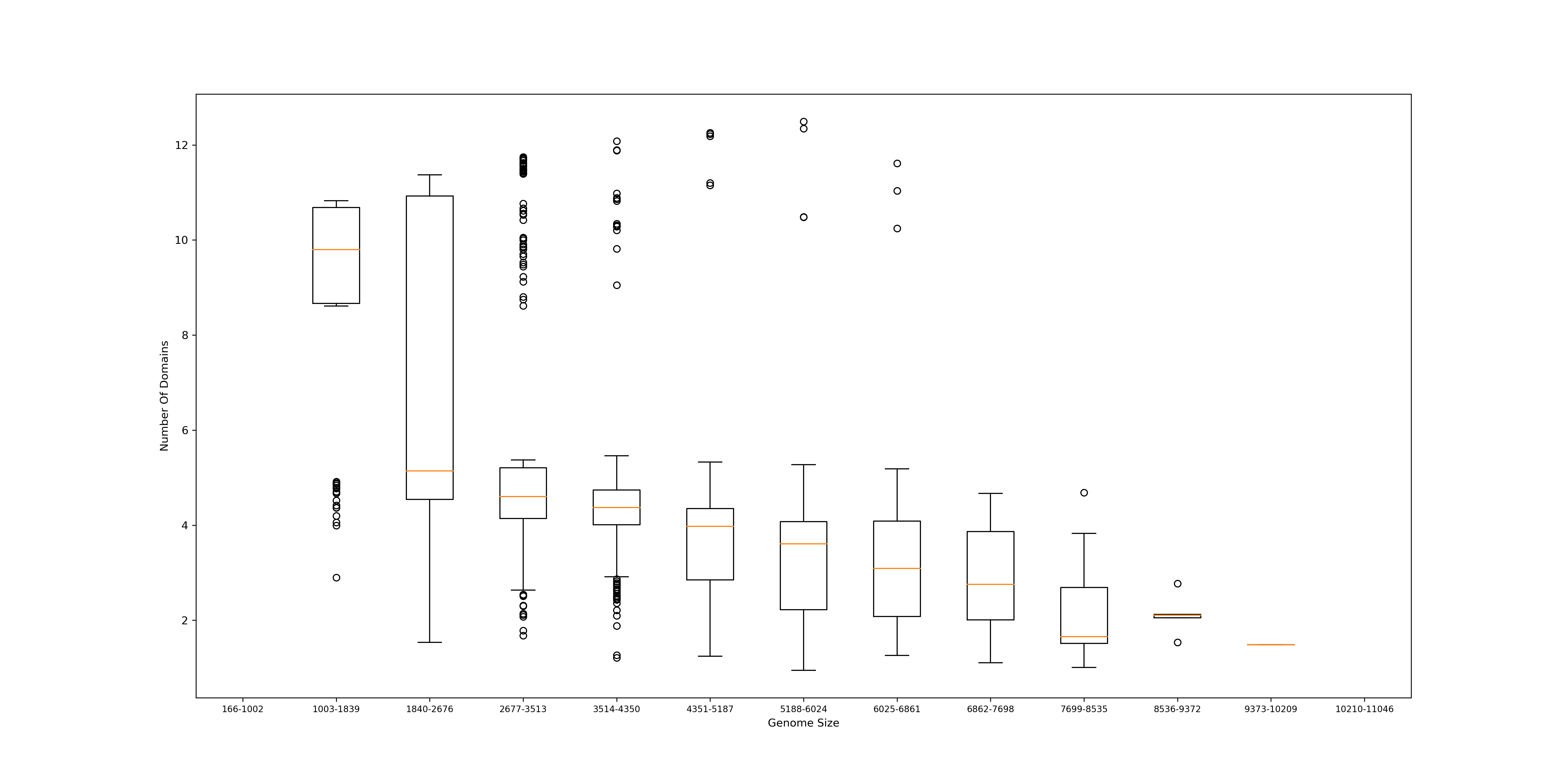

Supplement: S1 File — On the X-axis of each graph, genome size ranges are displayed in 13 windows, with a range of 836 ORFs each. On the Y-axis are the WDASs. The lines shown in the boxes are the median values. The whisker caps represent the minimum and maximum values. Superfamily IDs correspond to the names in Table 1. (ZIP) [file pone.0226604.s006.zip › Supplemnetary_material_S1/Figure_WSByIntervals_48498.png]

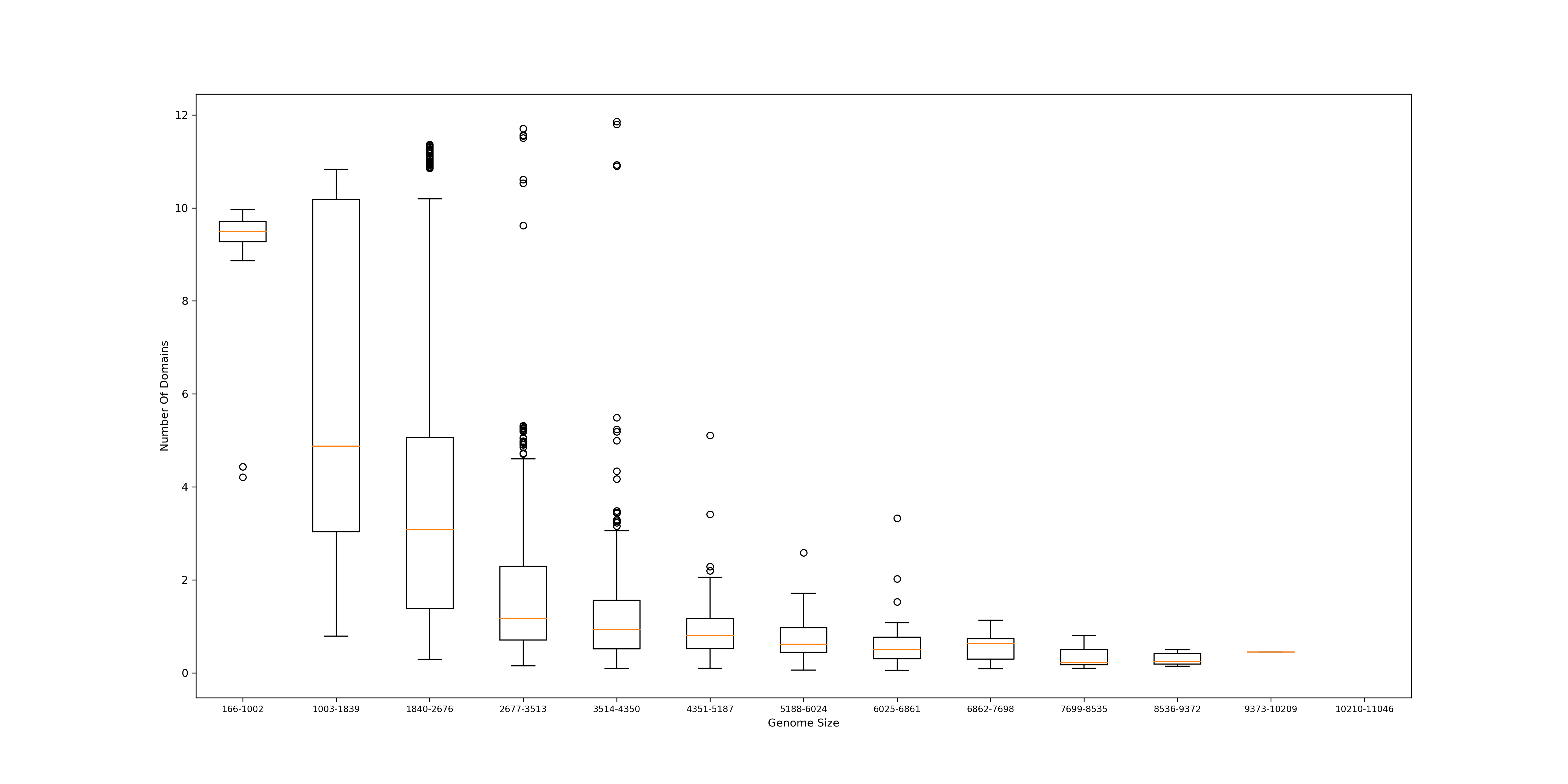

Supplement: S1 File — On the X-axis of each graph, genome size ranges are displayed in 13 windows, with a range of 836 ORFs each. On the Y-axis are the WDASs. The lines shown in the boxes are the median values. The whisker caps represent the minimum and maximum values. Superfamily IDs correspond to the names in Table 1. (ZIP) [file pone.0226604.s006.zip › Supplemnetary_material_S1/Figure_WSByIntervals_55781.png]

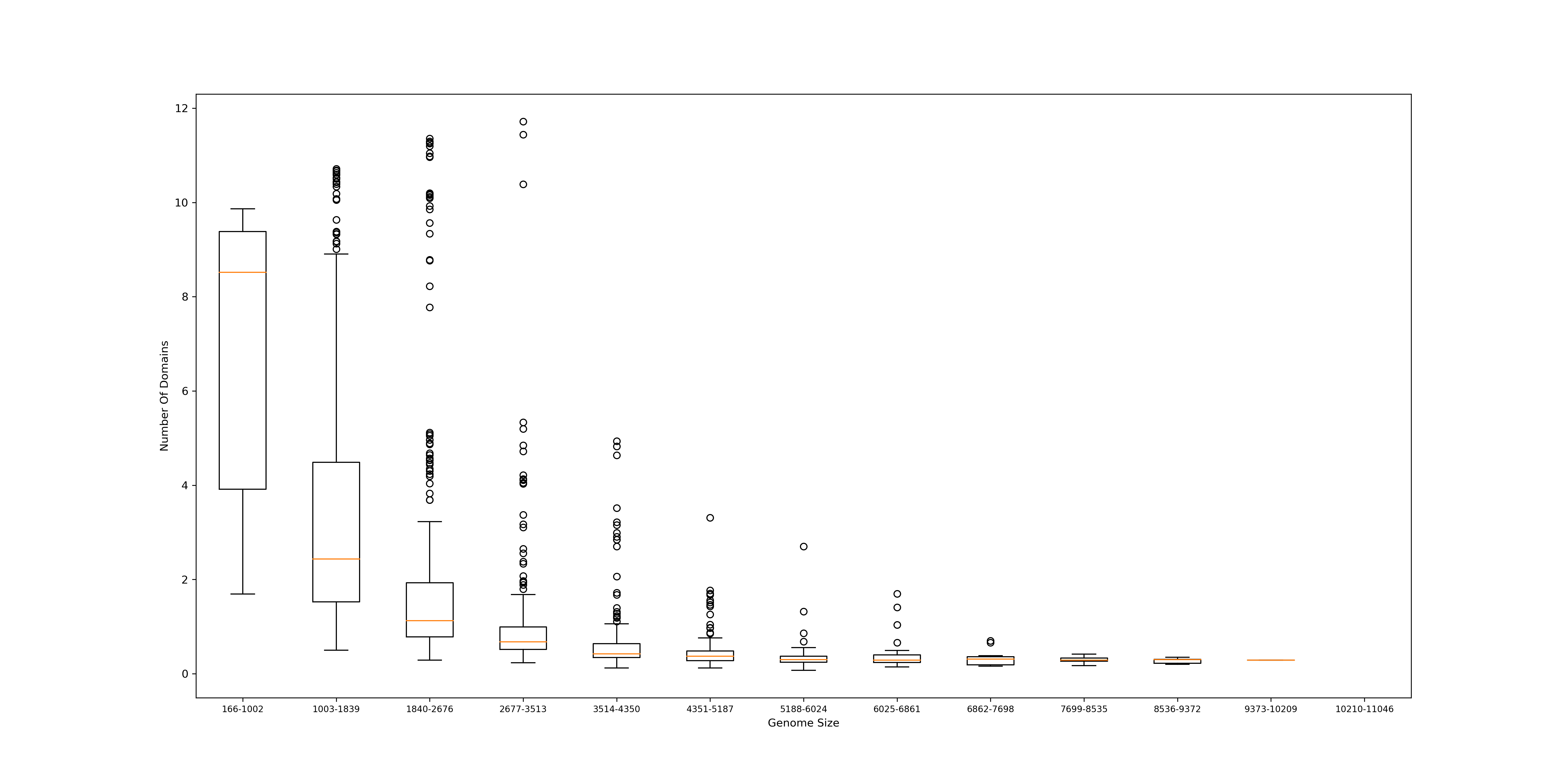

Supplement: S1 File — On the X-axis of each graph, genome size ranges are displayed in 13 windows, with a range of 836 ORFs each. On the Y-axis are the WDASs. The lines shown in the boxes are the median values. The whisker caps represent the minimum and maximum values. Superfamily IDs correspond to the names in Table 1. (ZIP) [file pone.0226604.s006.zip › Supplemnetary_material_S1/Figure_WSByIntervals_46689.png]

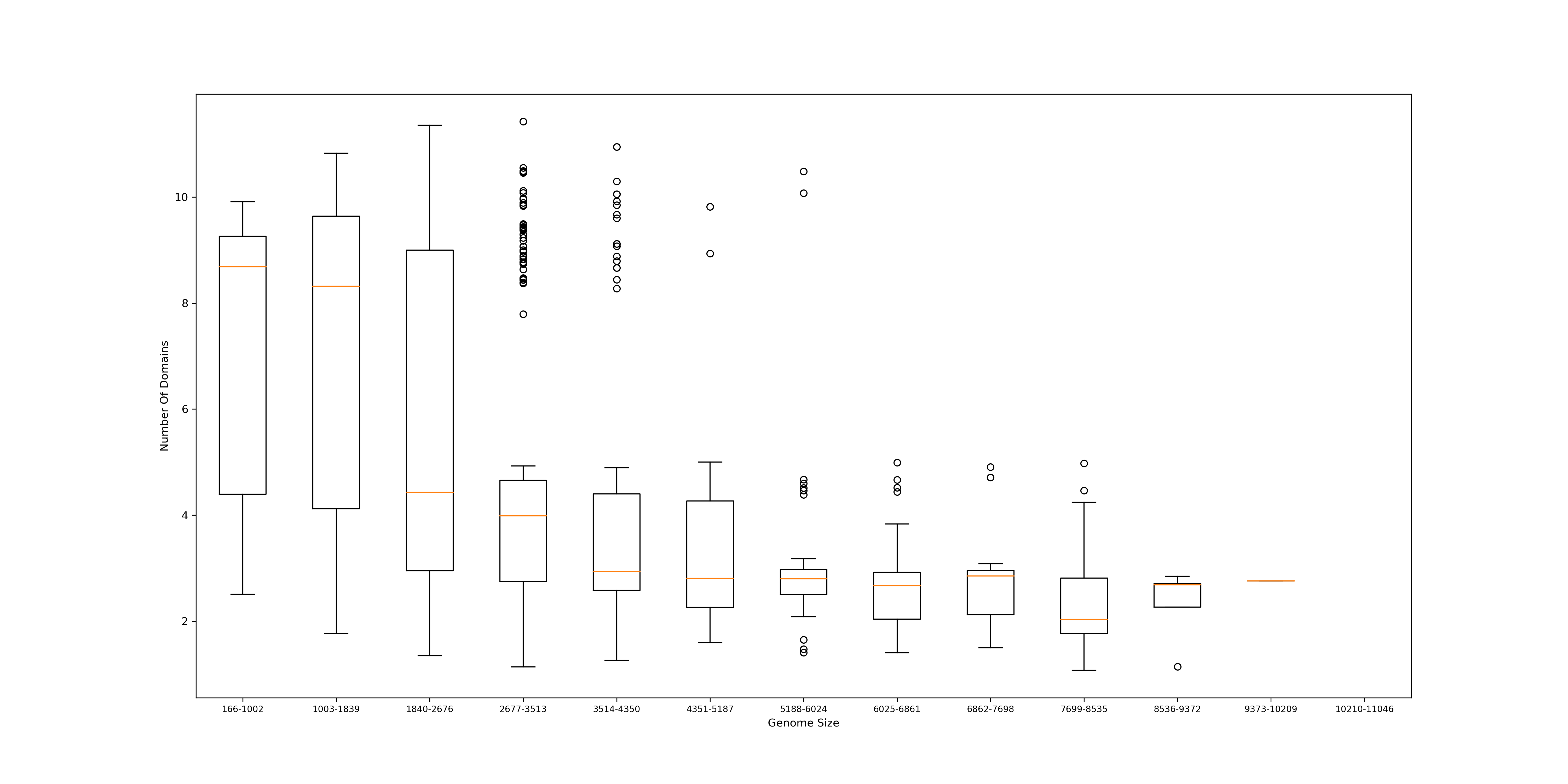

Supplement: S1 File — On the X-axis of each graph, genome size ranges are displayed in 13 windows, with a range of 836 ORFs each. On the Y-axis are the WDASs. The lines shown in the boxes are the median values. The whisker caps represent the minimum and maximum values. Superfamily IDs correspond to the names in Table 1. (ZIP) [file pone.0226604.s006.zip › Supplemnetary_material_S1/Figure_WSByIntervals_55811.png]

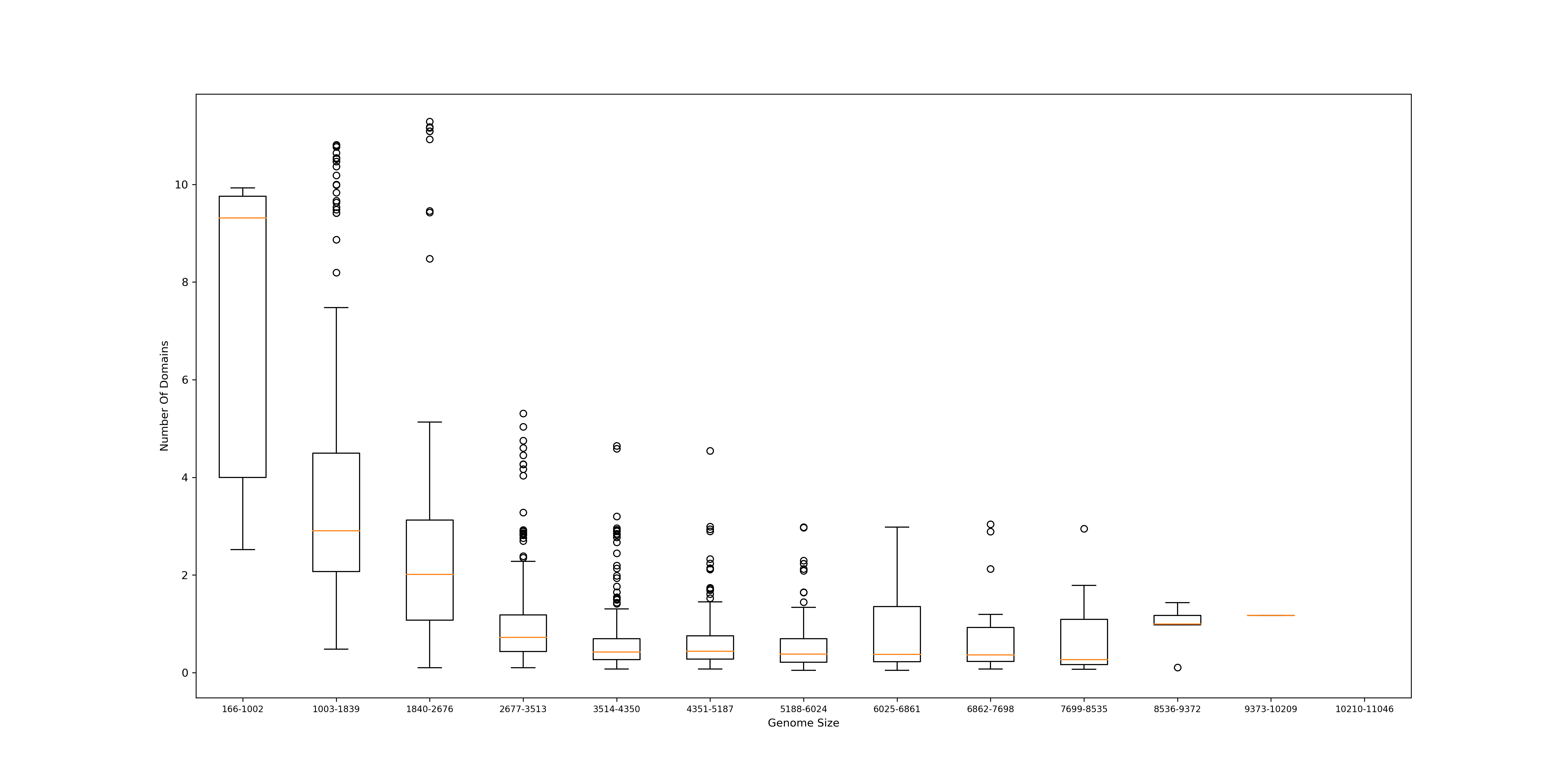

Supplement: S1 File — On the X-axis of each graph, genome size ranges are displayed in 13 windows, with a range of 836 ORFs each. On the Y-axis are the WDASs. The lines shown in the boxes are the median values. The whisker caps represent the minimum and maximum values. Superfamily IDs correspond to the names in Table 1. (ZIP) [file pone.0226604.s006.zip › Supplemnetary_material_S1/Figure_WSByIntervals_47384.png]
